# The global burden of osteoarthritis knee: a secondary data analysis of a population-based study

**DOI:** 10.1007/s10067-025-07347-6

**Published:** 2025-02-12

**Authors:** Jia-Le Ren, Junnan Yang, Wan Hu

**Affiliations:** 1 Department of Rheumatology & Immunology, The First Affiliated Hospital of Anhui Medical University, No.218, Ji-Xi Road, Hefei, 230022 China; 2 School of Public Health, BengBu Medical University, 2600 Donghai Avenue, Bengbu, 233030 Anhui China; 3 Department of Epidemiology and Biostatistics, School of Public Health, Anhui Medical University, 81 Meishan Road, Hefei, 230032 Anhui China

**Keywords:** Osteoarthritis, Knee, Disease burden, GBD

## Abstract

**Background:**

Osteoarthritis knee poses a substantial and pervasive global health challenge.

**Methods:**

The data was extracted from the Global Burden of Disease 2021 Study database. First, numbers and age-standardized rates (ASRs) of incidence, prevalence, and disability-adjusted life years (DALYs) of osteoarthritis knee were assessed globally and by sub-types in 2021. Subsequently, we employed a linear regression model to analyze the temporal trends from 1990 to 2021. To predict the future burden, we utilized the age-period-cohort model and the Bayesian age-period-cohort model. Furthermore, we conducted a sensitivity analysis using the Autoregressive Integrated Moving Average model and the Exponential Smoothing model.

**Results:**

In 2021, osteoarthritis knee accounted for 30.85 million incidence cases, 374.74 million prevalence cases, and 12.02 million DALYs cases globally, with ASRs of 353.67, 4294.27, and 137.59, respectively. Females and individuals over 50 years old were identified as high-risk populations, while higher socio-demographic index regions emerged as high-risk areas. From 1990 to 2021, incidence cases rose from 14.13 million to 30.85 million, prevalence cases from 159.80 million to 374.74 million, and DALYs cases from 5.15 million to 12.02 million, accompanied by increases in their respective ASRs. Projections using the APC model predict a continued increase in incidence, prevalence, and DALYs cases for both genders until 2046. Specifically, male incidence cases are projected to increase to 18.45 million and female incidence to 25.60 million. Similarly, male prevalence cases are projected to rise to 235.41 million and female prevalence to 365.97 million. Male DALYs cases are expected to increase to 7.52 million and female DALYs to 11.55 million. The BAPC models also indicate an upward trend in number of cases.

**Conclusion:**

In conclusion, osteoarthritis knee represents a formidable threat to global public health, necessitating the development of proactive and tailored strategic interventions that account for global-specific contexts.

**Table:** 

**Key Points** • *Females and individuals over 50 years old were identified as high-risk populations*. • *Higher socio-demographic index regions were identified as high-risk areas*. • *The disease burden attributable to osteoarthritis knee increased from 1990 to 2019*. • *The number of deaths and DALYs cases would still increase in the next 25 years*.

**Supplementary Information:**

The online version contains supplementary material available at 10.1007/s10067-025-07347-6.

## Introduction

Osteoarthritis knee, a chronic and degenerative joint disease [1, 2], stands as a formidable health challenge across the globe [3]. It is characterized by the gradual breakdown of cartilage in the knee joint, leading to pain, stiffness, and functional impairment, ultimately affecting an individual's quality of life and mobility [1, 4, 5]. The global prevalence of osteoarthritis knee has reached epidemic proportions, with estimates suggesting that it affects millions of people worldwide, imposing a significant burden on individuals, healthcare systems, and economies [6–8].

The period from 1990 to 2021 has witnessed profound changes in the global population demographics, lifestyles, and healthcare systems [9–11], all of which have had a profound impact on the prevalence and burden of osteoarthritis knee. The aging of the population, coupled with increasing sedentary behaviors and the rise in obesity rates, has fueled the growth of osteoarthritis knee [11–13]. Additionally, advances in diagnostic technologies and the introduction of novel therapeutic options have transformed the management of osteoarthritis knee [14–16], yet their impact on the overall burden remains to be fully elucidated.

Against this backdrop, a deeper understanding of the global burden of osteoarthritis knee, as reflected in the Global Burden of Disease (GBD) Study 1990–2021, is imperative. By examining the trends in the incidence, prevalence, and disability associated with osteoarthritis knee across different regions, age groups, and sexes, we can gain valuable insights into the evolving nature of this disease. Furthermore, by exploring the potential drivers of these trends, we can identify key areas for intervention and develop targeted strategies to mitigate the burden of osteoarthritis knee.

This study seeks to contribute to the growing body of knowledge on the global burden of osteoarthritis knee by analyzing the data from the GBD Study 1990–2021. We aim to provide a comprehensive overview of the current state of osteoarthritis knee, highlighting the disparities in its burden across different populations and time points. By sharing the lessons learned from the GBD Study, we hope to inspire further research, inform policy decisions, and ultimately, improve the lives of those affected by this debilitating condition.

## Methods

### Data sources and extraction

This study was a secondary data analysis of a population-based study named the GBD 2021 Study. The annual counts of cases and the corresponding age-standardized rates (ASRs) pertaining to osteoarthritis knee, both globally and by subtypes, were retrieved from the GBD 2021 Study database [17]. The GBD 2021 Study, which was carried out by an independent population health research organization based at the University of Washington School of Medicine, Institute for Health Metrics and Evaluation [17–19]. Initiated in 1990 and regularly updated, the GBD Study has emerged as a cornerstone in comprehending the global health scenario [18]. Through rigorous quantification of health losses attributed to diverse diseases, injuries, and risk factors, the GBD Study furnishes a robust framework for monitoring trends, identifying disparities, and informing policy interventions [17, 19]. Over the past three decades, the GBD Study's scope has expanded to encompass a wider array of health outcomes, including non-fatal outcomes like disability, thereby offering a holistic perspective on the burden of diseases [17, 19].

The GBD 2021 dataset encompassed data spanning 1990 to 2021 for 204 countries and territories, which were further categorized into 28 GBD super regions and 54 GBD regions based on geographical considerations [17]. Additionally, these countries and territories were grouped into five regions according to their socio-demographic index (SDI) values [20, 21].

For this study, we adopted the human development index (HDI), a metric introduced by the United Nations Development Programme (UNDP) in 1990 [22, 23]. The HDI is a composite indicator encompassing education, life expectancy, and gross national income, serving as a comprehensive measure of the economic and social development level of United Nations member states [22, 23]. Since its inception, the HDI has played a pivotal role in guiding developing countries in devising appropriate development strategies [22, 23]. Annually, the UNDP publishes the HDI for countries worldwide and utilizes it to assess the human development status of each country in the Human Development Report [24, 25].

### Statistical analysis

In this study, we initially reported the incidence, prevalence, and Disability-Adjusted Life Years (DALYs) attributed to osteoarthritis knee, accompanied by their respective ASRs, for the year 2021. This analysis encompassed a global perspective and was further stratified by various subtypes, encompassing age groups, sex, SDI regions, GBD regions, and individual countries.

Subsequently, we delved into investigating temporal trends in osteoarthritis knee burden, both globally and within specified subtypes, spanning from 1990 to 2021. To quantify the rate of change in this burden over the study period, we estimated the Estimated Annual Percentage Change (EAPC) utilizing a linear regression model. Based on the EAPC values obtained, we conducted a hierarchical cluster analysis to systematically categorize the 54 GBD regions into four distinct clusters: those exhibiting a significant increase, a minor increase, remaining stable or experiencing a minor decrease, and those with a significant decrease in osteoarthritis knee burden. To probe potential associations between EAPCs and socioeconomic factors, we examined the correlation between EAPCs and osteoarthritis knee-related ASRs in 1990, as well as HDI scores in 2021. Given the non-normal distribution of these variables, we adopted the Spearman correlation analysis, a non-parametric approach, to assess the strength and direction of these associations.

Furthermore, we employed two advanced time-series models to forecast the future burden of osteoarthritis knee. These models included the Age-Period-Cohort (APC) model under the maximum likelihood framework and the Bayesian APC (BAPC) model, which was integrated with nested Laplace approximations. To ensure the robustness of our predictions, we also conducted a sensitivity analysis utilizing Autoregressive Integrated Moving Average (ARIMA) and Exponential Smoothing (ES) models.

Statistical significance was set at 0.05 (two-tailed). All the analyses were performed (or conducted) using R software (version 4.0.2). The “tidyverse” package in R was used to construct linear regression models to calculate EAPC values, the “cluster” package was used for k-mean clustering analysis, the “nordpred” package was used to construct the APC model, the “BAPC” and “INLA” packages were used for the BAPC model, and the “forecast” package was used to construct the ARIMA model and ES model.

## Results

### The disease burden of osteoarthritis knee in 2021

In 2021, the number of incidence cases of osteoarthritis knee was 30.85 million [95% uncertainty interval (UI): 26.53–35.19] globally. The corresponding age-standardized incidence rate (ASIR) was 353.67 (95% UI: 304.56–402.5) per 100,000 population. The number of prevalence cases was 374.74 million (95% UI: 321.86–428.35) globally. The corresponding age-standardized prevalence rate (ASPR) was 4294.27 (95% UI: 3695.04–4910.76) per 100,000 population. Moreover, the number of osteoarthritis knee-related DALYs was 12.02 million (95% UI: 5.86–23.27) globally in 2021. The corresponding ASR of DALYs was 137.59 (95% UI: 67.08–266.87) per 100,000 population (Tables 1, 2, and 3).

**Table 1:** The number of incidence cases and the age-standardized incidence rate attributable to osteoarthritis knee in 1990 and 2021, and its trends from 1990 to 2021 globally

	Number of incidence cases (95% UI) in 1990	The age-standardized incidence rate/100000 (95% UI) in 1990	Number of incidence cases (95% UI) in 2021	The age-standardized incidence rate/100000 (95% UI) in 2021	EAPC (95% CI)
Global	14134284 (12150933–16079381)	330.26 (284.34–375.75)	30845891 (26534151–35188905)	353.67 (304.56–402.50)	0.28 (0.26–0.3)
Sex					
Female	8393838 (7253340–9518118)	384.14 (331.52–436.38)	18299871 (15784959–20836893)	410.51 (354.20–467.16)	0.29 (0.26–0.32)
Male	5740445 (4906389–6564723)	275.13 (236.09–313.58)	12546020 (10759562–14371725)	295.15 (253.69–336.09)	0.26 (0.25–0.28)
Age					
< 5 years	0 (0–0)	0 (0–0)	0 (0–0)	0 (0–0)	0 (0–0)
5–9 years	0 (0–0)	0 (0–0)	0 (0–0)	0 (0–0)	0 (0–0)
10–14 years	0 (0–0)	0 (0–0)	0 (0–0)	0 (0–0)	0 (0–0)
15–19 years	0 (0–0)	0 (0–0)	0 (0–0)	0 (0–0)	0 (0–0)
20–24 years	0 (0–0)	0 (0–0)	0 (0–0)	0 (0–0)	0 (0–0)
25–29 years	0 (0–0)	0 (0–0)	0 (0–0)	0 (0–0)	0 (0–0)
30–34 years	293483 (214102–393948)	76.15 (55.55–102.21)	498524 (363303–665109)	82.47 (60.10–110.03)	0.4 (0.29–0.50)
35–39 years	1082991 (784876–1448220)	307.45 (222.82–411.14)	1839845 (1337268–2449364)	328.04 (238.43–436.71)	0.42 (0.28–0.56)
40–44 years	1688097 (1379045–2035335)	589.25 (481.37–710.46)	3109642 (2528367–3749837)	621.62 (505.42–749.59)	0.41 (0.28–0.53)
45–49 years	2090077 (1464174–2853858)	900.14 (630.58–1229.08)	4616869 (3240418–6267812)	975.04 (684.35–1323.71)	0.42 (0.35–0.50)
50–54 years	2217245 (1678395–2819585)	1043.06 (789.57–1326.42)	5102428 (3861629–6505792)	1146.81 (867.93–1462.23)	0.35 (0.31–0.39)
55–59 years	1982053 (1434441–2754152)	1070.22 (774.54–1487.12)	4552167 (3295867–6263869)	1150.33 (832.86–1582.87)	0.24 (0.21–0.28)
60–64 years	1726190 (1254590–2289805)	1074.78 (781.14–1425.7)	3648066 (2647199–4852237)	1139.85 (827.13–1516.1)	0.17 (0.13–0.21)
65–69 years	1320519 (967603–1762094)	1068.3 (782.79–1425.53)	3046132 (2230050–4065887)	1104.3 (808.45–1473.99)	0.06 (0.01–0.10)
70–74 years	845839 (613899–1136654)	999.09 (725.13–1342.59)	2163205 (1567605–2904902)	1050.92 (761.57–1411.25)	0.06 (0–0.11)
75–79 years	544046 (387506–756848)	883.83 (629.52–1229.54)	1248506 (885822–1732941)	946.67 (671.67–1313.98)	0.17 (0.15–0.19)
80–84 years	250747 (167439–360507)	708.81 (473.31–1019.07)	681029 (454653–966341)	777.58 (519.11–1103.34)	0.29 (0.28–0.3)
85–89 years	74635 (52286–101998)	493.91 (346.01–674.99)	250460 (175802–341155)	547.79 (384.5–746.15)	0.35 (0.33–0.37)
90–94 years	15233 (9701–23181)	355.49 (226.39–540.95)	70627 (44941–107423)	394.8 (251.22–600.48)	0.4 (0.36–0.43)
95 + years	3130 (1738–4978)	307.39 (170.72–488.91)	18390 (10164–29240)	337.41 (186.49–536.47)	0.37 (0.33–0.4)
SDI region					
High-middle SDI	3321371 (2852523–3782455)	321.69 (277.21–366.51)	6758885 (5792871–7757737)	357.78 (307.34–407.91)	0.44 (0.39–0.49)
High SDI	3765186 (3281345–4286856)	363.52 (313.7–414.49)	6502877 (5683246–7463504)	386.58 (334.58–439.34)	0.14 (0.1–0.17)
Low-middle SDI	2125659 (1820455–2420457)	294.4 (252.81–334.62)	5255956 (4503893–5974804)	321.83 (276.69–366.11)	0.31 (0.3–0.32)
Low SDI	778637 (667577–888772)	288.29 (248.25–328.53)	1931578 (1655106–2209900)	306.06 (264.24–347.69)	0.2 (0.2–0.21)
Middle SDI	4130154 (3517288–4720592)	338.37 (291.05–386.6)	10373196 (8911389–11926381)	359.69 (310.37–410.52)	0.33 (0.28–0.38)
GBD region					
Advanced Health System	5290490 (4591745–6040694)	340.02 (293.52–387.26)	8588050 (7503168–9861402)	366.49 (316.98–416.65)	0.19 (0.16–0.21)
Africa	978776 (835178–1121887)	293.41 (251.87–336.58)	2609781 (2217455–2986929)	317.33 (272.01–363.93)	0.25 (0.24–0.26)
African Region	770639 (657569–883437)	294.47 (252.65–338.32)	2043403 (1738803–2339526)	315.99 (270.77–362.52)	0.23 (0.22–0.24)
America	2184521 (1900256–2477382)	357.2 (308.12–407.57)	4860589 (4189761–5564405)	384.31 (331.18–438.07)	0.14 (0.06–0.21)
Andean Latin America	80832 (69708–92097)	348.24 (300.96–396.58)	242602 (209062–279223)	386.9 (333.29–443.77)	0.37 (0.36–0.38)
Asia	7939107 (6769035–9070161)	335.97 (289.69–382.89)	18942723 (16329239–21698698)	356.44 (307.92–406.02)	0.31 (0.26–0.36)
Australasia	80753 (70372–92095)	359.26 (310.77–412.04)	177000 (153385–205240)	403.34 (348.15–463.45)	0.34 (0.32–0.37)
Basic Health System	5810661 (4952057–6655844)	340 (292.31–388.86)	14385251 (12313505–16548102)	364.82 (313.31–417.49)	0.38 (0.32–0.44)
Caribbean	94617 (81658–107550)	348.79 (300.57–396.78)	199696 (172770–230459)	374.83 (324.54–430.56)	0.27 (0.25–0.28)
Central Africa	98842 (84122–113344)	293.07 (251.11–336.43)	267720 (228735–304682)	304.94 (262.67–347.31)	0.1 (0.09–0.12)
Central Asia	111442 (96037–128400)	225.44 (194.93–257.32)	222031 (188796–255746)	239.29 (205.97–274.09)	0.2 (0.18–0.21)
Central Europe	392665 (336818–453197)	263.75 (226.58–302.68)	529220 (459378–611643)	282.04 (242.21–323.09)	0.23 (0.22–0.23)
Central Latin America	340356 (292287–386909)	354.35 (305.58–404.67)	1014222 (871109–1156490)	383.48 (330.54–435.99)	0.26 (0.25–0.27)
Central Sub-Saharan Africa	79094 (67356–90793)	286.39 (245.77–329.26)	217592 (185701–248119)	297.77 (256.63–338.31)	0.11 (0.09–0.12)
Commonwealth High Income	440063 (384625–503579)	325.2 (282.73–370.39)	780745 (679936–898203)	353.81 (306.49–401.55)	0.23 (0.2–0.26)
Commonwealth Low Income	292667 (252373–331056)	283.69 (244.96–321.78)	824613 (709856–939537)	305.04 (264.02–346.25)	0.26 (0.25–0.27)
Commonwealth Middle Income	2254365 (1924432–2566296)	297.66 (255.93–338.88)	5827317 (5007354–6622320)	325.42 (280.43–370.4)	0.3 (0.29–0.31)
East Asia	3787399 (3238108–4358778)	377.47 (324.32–433.77)	8800266 (7530686–10174644)	406.19 (348.6–466.86)	0.46 (0.37–0.56)
East Asia & Pacific—WB	5524468 (4722340–6339513)	361.22 (311.55–413.4)	12465448 (10707734–14343814)	380.69 (327.02–436.15)	0.33 (0.26–0.4)
Eastern Africa	232276 (198686–266043)	279.54 (240.81–319.66)	616482 (525894–707169)	301.07 (258.77–344.61)	0.26 (0.25–0.27)
Eastern Europe	769391 (659450–894233)	278.19 (239.66–320.43)	958135 (823739–1109492)	298.56 (256.44–342.29)	0.25 (0.24–0.27)
Eastern Mediterranean Region	584012 (499407–668266)	276.47 (238.24–316.99)	1799297 (1528476–2065641)	310.32 (266.83–355.24)	0.39 (0.38–0.39)
Eastern Sub-Saharan Africa	252480 (215755–289261)	281.41 (242.27–322.52)	652313 (555382–747784)	299.96 (257.48–343.27)	0.23 (0.21–0.24)
Europe	3003746 (2600797–3459175)	305.21 (263.34–348.02)	4385461 (3831738–5063293)	329.71 (285.38–375.66)	0.24 (0.23–0.26)
Europe & Central Asia—WB	3072952 (2660566–3537911)	302.57 (261.08–345.09)	4537480 (3960634–5234358)	324.93 (281.19–370.32)	0.22 (0.21–0.24)
European Region	3094795 (2679952–3563070)	302.64 (261.14–345.12)	4587590 (4003845–5291092)	325.07 (281.28–370.45)	0.23 (0.21–0.24)
High-income Asia Pacific	928187 (799581–1062050)	441.54 (382.22–504.23)	1482839 (1312650–1690590)	458.22 (397.65–522.59)	0.16 (0.12–0.2)
High-income North America	1160725 (1017358–1319031)	367.48 (317.93–419.67)	2103504 (1830759–2428582)	389.08 (335.14–444.02)	−0.06 (−0.22–0.11)
Latin America & Caribbean—WB	1037972 (897683–1177876)	349.51 (301.7–399.07)	2779608 (2393193–3170544)	382.64 (330.4–435.79)	0.3 (0.29–0.32)
Limited Health System	2817957 (2406950–3207016)	294.99 (253.56–335.28)	7357573 (6321588–8363822)	321.19 (276.79–365.68)	0.29 (0.28–0.3)
Middle East & North Africa—WB	423281 (364315–484814)	292.11 (252.22–333.67)	1394900 (1188014–1598453)	324.43 (278.52–371.4)	0.33 (0.32–0.34)
Minimal Health System	201899 (171747–230930)	285.53 (244.57–326.76)	491617 (417987–562175)	300.72 (258.26–342.82)	0.17 (0.16–0.17)
North Africa and Middle East	578152 (495303–662749)	292.26 (251.18–334.1)	1811718 (1543012–2081311)	325.34 (279.63–371.94)	0.35 (0.34–0.35)
North America	1160848 (1017463–1319173)	367.51 (317.95–419.7)	2103721 (1830950–2428834)	389.1 (335.15–444.04)	−0.06 (−0.22–0.11)
Northern Africa	212810 (183007–244143)	293.35 (253.7–335.43)	605433 (514727–698068)	327.06 (281.2–376.2)	0.33 (0.32–0.35)
Oceania	11950 (10219–13792)	312.73 (269.5–359.55)	33635 (28494–39060)	334.57 (286.05–387.71)	0.18 (0.16–0.21)
Region of the Americas	2184521 (1900256–2477382)	357.2 (308.12–407.57)	4860589 (4189761–5564405)	384.31 (331.18–438.07)	0.14 (0.06–0.21)
South-East Asia Region	2500639 (2129061–2844512)	290.52 (249.17–330.24)	6457558 (5549606–7347320)	318.3 (274.38–361.97)	0.32 (0.31–0.33)
South Asia	2074360 (1774240–2358850)	296.19 (255.45–336.72)	5405132 (4656883–6153076)	324.2 (280.14–369.04)	0.31 (0.29–0.32)
South Asia—WB	2127221 (1817442–2417567)	295.13 (254.35–335.46)	5521845 (4754916–6286338)	323.07 (279.09–367.73)	0.31 (0.29–0.32)
Southeast Asia	750827 (639280–860351)	247.53 (213.1–282.67)	2050674 (1744848–2371714)	274.07 (235.74–314.07)	0.37 (0.36–0.38)
Southern Africa	154462 (131846–176761)	301.89 (258.71–346.61)	377405 (322077–430122)	323.26 (277.85–370.77)	0.23 (0.23–0.24)
Southern Latin America	163441 (141110–187399)	348.5 (300.96–399.28)	315715 (274143–360911)	387.34 (336.02–441.16)	0.31 (0.27–0.34)
Southern Sub-Saharan Africa	96752 (82606–111033)	315.17 (269.79–363.44)	227947 (195149–260792)	338.1 (290.72–388.21)	0.24 (0.23–0.24)
Sub-Saharan Africa—WB	768935 (655794–881171)	293.56 (251.8–337.09)	2010284 (1711968–2301120)	314.75 (269.99–360.78)	0.23 (0.22–0.24)
Tropical Latin America	362189 (310489–413559)	346.45 (297.89–397.37)	1014593 (871428–1161138)	380.8 (327.9–434.95)	0.34 (0.32–0.35)
Western Africa	280386 (239167–321971)	301.46 (258.61–346.65)	742741 (630820–853543)	326.01 (280.35–375.05)	0.25 (0.2–0.29)
Western Europe	1708316 (1486092–1964832)	334.48 (289.7–381.98)	2555151 (2238318–2953418)	357.68 (311.46–406.98)	0.19 (0.17–0.21)
Western Pacific Region	4905588 (4195139–5629746)	377.45 (325.24–432.09)	10894115 (9363079–12519683)	397.77 (341.42–455.45)	0.35 (0.27–0.43)
Western Sub-Saharan Africa	310356 (264842–356491)	302.36 (259.52–347.66)	831905 (706430–953808)	326.72 (280.96–375.13)	0.24 (0.2–0.28)
World Bank High Income	4377972 (3815784–4990393)	356.38 (308–405.47)	7281768 (6375936–8356514)	380.63 (329.4–431.77)	0.14 (0.1–0.17)
World Bank Low Income	474980 (404411–544456)	291.25 (250.2–333.31)	1157018 (984007–1323264)	306.67 (263.09–350.03)	0.19 (0.18–0.2)
World Bank Lower Middle Income	3605272 (3086185–4100029)	284.68 (244.48–322.99)	9329454 (7997910–10641262)	310.88 (267.57–354.01)	0.3 (0.29–0.31)
World Bank Upper Middle Income	5662722 (4831771–6486043)	349.11 (299.89–399.64)	13054147 (11211036–14986772)	381.3 (327.38–436.42)	0.45 (0.38–0.51)
Country					
American Samoa	105 (90–121)	358.29 (307.17–411.51)	212 (183–245)	389.31 (336.72–448.09)	0.23 (0.16–0.29)
Antigua and Barbuda	170 (149–193)	349.59 (301.53–401.45)	431 (369–500)	375.78 (323.32–432.73)	0.23 (0.22–0.25)
Arab Republic of Egypt	101598 (86890–116817)	298.3 (257.81–344.66)	269387 (226860–312111)	331.78 (285.65–383.43)	0.29 (0.27–0.32)
Argentine Republic	112317 (96777–128791)	345.81 (298.79–396.14)	202138 (175236–230539)	383.85 (331.09–438.73)	0.3 (0.27–0.34)
Australia	67582 (58768–76997)	359.63 (310.78–412.57)	148591 (128797–171863)	404.82 (349.2–465.56)	0.35 (0.32–0.38)
Barbados	905 (788–1026)	367.12 (315.19–421.21)	1793 (1538–2091)	389.66 (335.28–447.18)	0.2 (0.19–0.21)
Belize	343 (298–388)	352.71 (305.15–400.64)	1381 (1190–1592)	390.91 (338.05–447.17)	0.32 (0.28–0.37)
Bermuda	252 (217–286)	383.55 (330.03–434.9)	445 (386–514)	401.17 (344.91–460.11)	0.14 (0.13–0.16)
Bolivarian Republic of Venezuela	39848 (34065–45350)	353 (305.3–403.53)	120295 (102819–136989)	378.51 (325.33–429.38)	0.23 (0.22–0.24)
Bosnia and Herzegovina	11681 (9831–13553)	253.94 (217.06–292.03)	14976 (12867–17359)	273.96 (234.64–315.52)	0.27 (0.24–0.29)
Brunei Darussalam	606 (522–688)	434.19 (378.46–496.57)	2159 (1866–2465)	468.07 (407.83–533.21)	0.23 (0.21–0.26)
Burkina Faso	14567 (12404–16666)	294.48 (252.52–336.81)	36539 (31105–42298)	319.48 (274.65–371.6)	0.26 (0.25–0.27)
Canada	66728 (58059–76170)	212.78 (184.78–244.68)	142784 (123124–164885)	242.01 (210.54–275.05)	0.23 (0.13–0.33)
Central African Republic	3980 (3388–4611)	279.21 (240.38–323.05)	8839 (7439–10255)	289.53 (249.51–335.13)	0.12 (0.11–0.13)
Commonwealth of Dominica	198 (172–226)	356.73 (307.94–408.67)	318 (273–370)	377.82 (327.15–433.11)	0.18 (0.16–0.2)
Commonwealth of the Bahamas	644 (556–740)	365.86 (315.29–420.77)	1777 (1503–2052)	388.62 (332.86–447.31)	0.2 (0.18–0.22)
Cook Islands	50 (42–57)	346.2 (296.82–397.14)	92 (78–107)	394.52 (335.65–451.29)	0.39 (0.35–0.43)
Czech Republic	34692 (29852–39891)	265.56 (227.18–306.26)	49899 (43491–57277)	282.63 (243.55–323.29)	0.2 (0.19–0.21)
Democratic People's Republic of Korea	70692 (59950–81929)	364.54 (312.97–417.9)	132067 (113167–153053)	378.9 (325.1–435.11)	0.15 (0.14–0.16)
Democratic Republic of Sao Tome and Principe	206 (177–238)	308.5 (264.96–355.14)	485 (415–557)	334.58 (286.17–383.72)	0.26 (0.25–0.26)
Democratic Republic of the Congo	54889 (46596–63245)	288.16 (247.73–331.16)	141872 (121356–162084)	293.84 (253.58–335.58)	0.03 (0.01–0.05)
Democratic Republic of Timor-Leste	996 (839–1154)	241.5 (206.52–277.57)	2365 (2011–2734)	255.62 (218.14–294.33)	0.2 (0.2–0.21)
Democratic Socialist Republic of Sri Lanka	32599 (27771–37578)	251.25 (213.72–288.95)	74277 (64182–85392)	276.31 (238.93–317.73)	0.35 (0.34–0.36)
Dominican Republic	14412 (12459–16398)	341.84 (295.45–389.88)	39798 (34175–46250)	377.3 (323.84–440)	0.35 (0.34–0.36)
Eastern Republic of Uruguay	12477 (10831–14376)	348.58 (302.8–399.49)	17665 (15404–20210)	388.86 (336.29–444.78)	0.32 (0.29–0.35)
Federal Democratic Republic of Ethiopia	67012 (56962–77311)	277.07 (238.67–316.55)	159439 (135970–183353)	294.68 (252.17–338.09)	0.22 (0.2–0.23)
Federal Democratic Republic of Nepal	32779 (28155–37137)	284.69 (246.22–322.6)	82910 (72417–94336)	319.76 (279.61–362.7)	0.41 (0.39–0.43)
Federal Republic of Germany	377450 (326328–436453)	335.54 (290.6–383.76)	518788 (453049–601412)	357.4 (310.48–407.04)	0.18 (0.16–0.19)
Federal Republic of Nigeria	154398 (132562–177990)	303.57 (261.99–349.88)	392935 (333643–451627)	328.28 (282.57–378.37)	0.25 (0.17–0.33)
Federal Republic of Somalia	9643 (8147–11147)	279.9 (240.07–323.46)	25303 (21447–29156)	289.11 (247.97–332.35)	0.13 (0.12–0.14)
Federated States of Micronesia	181 (155–206)	330.43 (283.09–379.43)	329 (277–383)	362.41 (310.29–417.85)	0.26 (0.21–0.31)
Federative Republic of Brazil	353720 (303116–404186)	346.47 (297.75–397.59)	990960 (850953–1134433)	381 (328.11–435.26)	0.34 (0.33–0.35)
French Republic	237055 (206503–272083)	327.17 (282.23–374.9)	366042 (317330–422304)	351.29 (305.13–401.98)	0.2 (0.18–0.21)
Gabonese Republic	1801 (1532–2091)	298.26 (254.48–344.14)	4336 (3720–4992)	333.07 (290.16–382.17)	0.34 (0.33–0.36)
Georgia	14542 (12524–17075)	232.6 (200.38–268.56)	12757 (10940–14968)	241.18 (206.02–279.95)	0.13 (0.12–0.14)
Grand Duchy of Luxembourg	1694 (1470–1943)	333.66 (289.06–383.24)	3394 (2991–3903)	353.59 (310.7–403.24)	0.15 (0.14–0.17)
Greenland	102 (88–117)	230.72 (199.7–264.46)	195 (168–231)	259.22 (226.22–296.58)	0.36 (0.34–0.38)
Grenada	212 (186–242)	343.28 (296.68–394.1)	454 (388–526)	371.76 (321.28–425.89)	0.25 (0.23–0.27)
Guam	344 (293–397)	342.98 (293.92–396.51)	749 (642–871)	381.7 (328.51–442.42)	0.35 (0.33–0.38)
Hashemite Kingdom of Jordan	5137 (4356–5858)	299.36 (258.15–339.06)	33489 (28613–38510)	335.18 (290.55–382.63)	0.39 (0.37–0.41)
Hellenic Republic	46996 (40857–54487)	333.97 (291.04–383.04)	62266 (54254–71220)	356.86 (309.41–407.92)	0.17 (0.14–0.2)
Hungary	37877 (32545–44157)	272.09 (234.56–314.58)	46060 (39846–53765)	285.94 (245.54–331.06)	0.16 (0.15–0.16)
Independent State of Papua New Guinea	7138 (6097–8260)	303.57 (260.45–348.99)	23184 (19568–26961)	324.49 (277.33–377.32)	0.18 (0.17–0.2)
Independent State of Samoa	328 (278–377)	343.67 (293.84–393.75)	600 (510–702)	366.61 (313.86–427.44)	0.16 (0.13–0.2)
Ireland	12533 (10873–14416)	332.84 (287.47–384.13)	25223 (22007–29036)	357.97 (311.27–409.67)	0.2 (0.19–0.22)
Islamic Republic of Afghanistan	19990 (16985–23406)	268.84 (230.88–309.03)	41067 (34271–47879)	294.91 (254.11–337.45)	0.38 (0.35–0.41)
Islamic Republic of Iran	86974 (74332–100448)	282.83 (243.4–324.87)	285543 (244647–326619)	312.39 (268.62–357.31)	0.32 (0.3–0.33)
Islamic Republic of Mauritania	3506 (3007–4026)	315.1 (270.77–362.96)	8729 (7434–10077)	342.68 (295.8–394.76)	0.24 (0.23–0.26)
Islamic Republic of Pakistan	159122 (133809–184427)	250.45 (211.9–291.24)	437654 (367264–505132)	282.24 (238.27–326.16)	0.46 (0.42–0.49)
Jamaica	5792 (5022–6587)	349.04 (299.58–399.91)	11594 (9987–13387)	377.94 (326.02–434.47)	0.29 (0.27–0.31)
Japan	740432 (636892–848733)	432.03 (373.15–493.42)	1005565 (887299–1143421)	441.94 (382.84–503.95)	0.07 (0.05–0.08)
Kingdom of Bahrain	851 (726–976)	305.89 (263.09–351.36)	5249 (4368–6078)	333.98 (287.08–380.46)	0.29 (0.28–0.29)
Kingdom of Belgium	44565 (38478–51315)	328.54 (282.48–378.48)	63987 (55538–73570)	352.59 (306.19–401.86)	0.18 (0.16–0.2)
Kingdom of Bhutan	917 (784–1049)	295.2 (256.62–335.7)	2242 (1954–2540)	326.53 (283.84–374.08)	0.35 (0.34–0.37)
Kingdom of Cambodia	13597 (11527–15671)	249.14 (212.51–285.96)	38624 (33063–44357)	266.76 (229.25–305.66)	0.25 (0.24–0.26)
Kingdom of Denmark	22161 (19186–25324)	319.51 (277.62–366.22)	31897 (27875–36767)	344 (299.02–391.96)	0.2 (0.16–0.23)
Kingdom of Eswatini	1154 (986–1340)	319.37 (274.58–370.15)	2463 (2115–2821)	343.78 (296.53–394.27)	0.18 (0.14–0.23)
Kingdom of Lesotho	2764 (2361–3211)	295.5 (252.96–344.05)	4074 (3521–4664)	323.02 (279.68–372.27)	0.29 (0.28–0.3)
Kingdom of Morocco	44946 (38648–51584)	284.74 (244.51–329.85)	121776 (103711–141056)	318.44 (272.04–367.01)	0.37 (0.36–0.37)
Kingdom of Norway	17919 (15722–20467)	324.99 (281.18–369.91)	28578 (24956–32980)	348.03 (303.04–397.69)	0.2 (0.19–0.22)
Kingdom of Saudi Arabia	23227 (19733–26608)	292.96 (252.01–333.13)	118181 (98498–138331)	340.16 (292.65–389.44)	0.48 (0.46–0.5)
Kingdom of Spain	164279 (142313–190084)	333.2 (287.55–384.57)	274790 (239368–316480)	355.54 (311.34–407.8)	0.16 (0.14–0.18)
Kingdom of Sweden	33469 (29005–39221)	272.38 (232.79–319.82)	47999 (41451–56247)	296.18 (252.23–346.27)	0.19 (0.1–0.29)
Kingdom of Thailand	115965 (98404–134134)	266.23 (226.95–305.78)	322176 (275913–376817)	309.76 (266.39–357)	0.54 (0.52–0.56)
Kingdom of the Netherlands	63111 (54668–72071)	344.33 (296.72–393.23)	103319 (89701–119147)	367.58 (317.34–418.82)	0.18 (0.17–0.19)
Kingdom of Tonga	210 (179–245)	341.15 (292.49–394.48)	320 (273–368)	373.23 (318.03–429.39)	0.22 (0.16–0.28)
Kyrgyz Republic	6808 (5858–7933)	221.13 (191.87–255.89)	13014 (11188–14968)	231.57 (199.58–266.61)	0.14 (0.13–0.15)
Lao People's Democratic Republic	5976 (5089–6896)	247.23 (212.25–284.83)	15647 (13248–18150)	269.08 (230.15–312.03)	0.33 (0.31–0.34)
Lebanese Republic	7013 (6001–8101)	290.87 (249.39–333.57)	19060 (16367–21887)	327.85 (279.89–380.52)	0.38 (0.33–0.43)
Malaysia	29980 (25583–34541)	261.49 (225.13–301.16)	91610 (78457–106272)	290.78 (250.65–337.31)	0.39 (0.38–0.4)
Mongolia	2495 (2136–2874)	218.32 (187.25–253.5)	6736 (5790–7796)	231.84 (200.31–266.61)	0.09 (0.01–0.16)
Montenegro	1751 (1499–2027)	269.45 (230.85–309.83)	2572 (2211–2999)	280.74 (241.89–325.06)	0.16 (0.15–0.18)
New Zealand	13171 (11494–15112)	357.26 (308.1–411.27)	28409 (24525–32809)	395.46 (342.58–452.77)	0.31 (0.28–0.33)
North Macedonia	5269 (4501–6201)	260.68 (223.39–304.77)	9099 (7788–10594)	275.71 (236.3–317.8)	0.2 (0.2–0.21)
Northern Mariana Islands	108 (91–126)	338.54 (292.18–387.16)	236 (201–278)	373.88 (321.08–430.24)	0.27 (0.21–0.32)
Palestine	2820 (2410–3231)	295.86 (254.59–340.32)	10367 (8850–11947)	315.79 (270.91–364.09)	0.18 (0.16–0.2)
People's Democratic Republic of Algeria	39966 (34217–45966)	288.95 (248.23–331.29)	136739 (116454–156716)	326.14 (277.77–375.71)	0.41 (0.4–0.42)
People's Republic of Bangladesh	157676 (135883–178672)	281.85 (243.79–318.73)	465724 (404057–526279)	306.77 (266.11–346.54)	0.31 (0.3–0.33)
People's Republic of China	3650857 (3122264–4200440)	377.93 (324.79–434.28)	8512397 (7279974–9840885)	406.42 (348.7–467.23)	0.47 (0.37–0.56)
Plurinational State of Bolivia	12671 (10970–14571)	339.77 (294.71–393.53)	38829 (33682–44575)	378.27 (329.74–438.3)	0.36 (0.35–0.37)
Portuguese Republic	42763 (36956–49397)	328.8 (286.14–378.04)	66043 (58141–76227)	356.7 (311.91–406.82)	0.2 (0.17–0.23)
Principality of Andorra	199 (172–232)	329.64 (283.88–385.94)	521 (449–604)	356.21 (308.03–408.4)	0.23 (0.21–0.25)
Principality of Monaco	181 (158–209)	346.28 (299.5–400.34)	260 (226–301)	366.8 (317.43–417.24)	0.15 (0.14–0.17)
Puerto Rico	13663 (11874–15666)	382.14 (331.17–438.91)	21696 (18926–24952)	413.31 (357.6–472.52)	0.29 (0.27–0.31)
Republic of Albania	5841 (5023–6691)	250.22 (216.44–287.34)	10669 (9191–12473)	271.28 (232.62–313.43)	0.29 (0.28–0.3)
Republic of Angola	14096 (11987–16348)	278.46 (239.35–321.06)	48488 (41396–55817)	303.14 (260.9–349.17)	0.29 (0.27–0.3)
Republic of Armenia	6470 (5563–7648)	220.31 (190.66–254.24)	9809 (8459–11455)	239.62 (209.11–276.64)	0.3 (0.3–0.31)
Republic of Austria	34076 (29563–38990)	332.33 (288.3–378.14)	51703 (44889–59759)	355.39 (308.57–407.86)	0.18 (0.17–0.19)
Republic of Azerbaijan	12041 (10164–13995)	223.76 (191.09–256.48)	28981 (24494–33705)	243.18 (209.77–280.09)	0.31 (0.28–0.34)
Republic of Belarus	35178 (30289–41125)	277.89 (239.63–319.39)	43766 (37720–51287)	297.84 (256.24–342.19)	0.25 (0.23–0.26)
Republic of Benin	6757 (5794–7737)	302.6 (259.19–345.95)	22034 (18694–25258)	333.07 (284.78–380.41)	0.31 (0.29–0.34)
Republic of Botswana	1967 (1690–2257)	297.52 (255.96–343.81)	6399 (5492–7336)	334.76 (289.67–386.2)	0.38 (0.36–0.4)
Republic of Bulgaria	32546 (27920–38042)	268.85 (232.4–308.34)	32938 (28790–38021)	279.87 (242.2–321.54)	0.12 (0.1–0.14)
Republic of Burundi	7390 (6366–8455)	278.92 (240.04–321.4)	18192 (15396–20934)	283.54 (243.39–328.64)	0.06 (0.05–0.06)
Republic of Cabo Verde	622 (542–725)	307.86 (264.48–358.26)	1702 (1435–1982)	336.02 (286.9–388.53)	0.28 (0.28–0.29)
Republic of Cameroon	17393 (14801–20020)	322.89 (277.77–372.71)	57492 (48938–66138)	344.74 (295.14–395.41)	0.19 (0.18–0.2)
Republic of Chad	8855 (7575–10255)	288.96 (247.13–335.69)	22448 (19184–25944)	302.5 (259.8–349.03)	0.13 (0.13–0.14)
Republic of Chile	38639 (33521–44445)	356.36 (309.54–409.76)	95895 (83094–111518)	394.63 (343.81–454.98)	0.29 (0.26–0.32)
Republic of Colombia	70664 (61360–80548)	343.74 (296.52–393.38)	207041 (179811–238409)	376.06 (326.06–431.58)	0.3 (0.28–0.31)
Republic of Costa Rica	6787 (5872–7723)	353.34 (306.21–404.11)	21166 (18367–24367)	384.54 (333.55–440.64)	0.27 (0.27–0.28)
Republic of Croatia	16600 (14204–19228)	263.62 (227.64–302.03)	19979 (17218–23146)	280.6 (241.43–322.44)	0.24 (0.23–0.25)
Republic of Cuba	35827 (31030–41025)	348.65 (301.68–401.09)	67270 (57860–77896)	379.4 (330.04–436.72)	0.31 (0.3–0.32)
Republic of Cyprus	2650 (2293–3040)	319.93 (277.09–365.96)	6708 (5888–7658)	347.41 (303.87–398.15)	0.25 (0.23–0.26)
Republic of C么te d'Ivoire	16563 (14082–19178)	304.63 (263.16–351.41)	50403 (42746–58562)	325.4 (279.86–377)	0.19 (0.18–0.21)
Republic of Djibouti	538 (456–618)	278.38 (238.86–320.55)	2792 (2360–3240)	305.45 (263.05–355.23)	0.33 (0.32–0.35)
Republic of Ecuador	21814 (18889–24751)	362.28 (314.16–414.57)	68020 (58665–77886)	398.74 (344.44–456.51)	0.36 (0.33–0.38)
Republic of El Salvador	11089 (9569–12635)	348.06 (299.85–398.25)	23114 (20070–26654)	384.55 (333.76–443.26)	0.33 (0.3–0.36)
Republic of Equatorial Guinea	653 (556–757)	281.98 (242.9–327.18)	2339 (1986–2679)	328.75 (282.88–379.49)	0.6 (0.56–0.64)
Republic of Estonia	5577 (4817–6459)	281.57 (243.97–325.76)	6330 (5476–7247)	303.49 (260.51–349.21)	0.28 (0.27–0.3)
Republic of Fiji	1626 (1384–1886)	331.6 (285.07–383.29)	3351 (2857–3902)	370.46 (318.85–427.34)	0.33 (0.29–0.36)
Republic of Finland	22072 (19332–25199)	334.07 (291–383)	32672 (28598–37499)	356.47 (311.02–409.69)	0.19 (0.17–0.2)
Republic of Ghana	23169 (19769–26938)	300.17 (258.05–346.15)	72773 (61958–83998)	337.69 (289.41–389.59)	0.37 (0.36–0.38)
Republic of Guatemala	13808 (11857–15777)	330.96 (286.41–380.32)	43808 (37757–50068)	363.33 (311.83–417.15)	0.3 (0.28–0.31)
Republic of Guinea	10718 (9220–12396)	297.29 (255.59–343.71)	21389 (18325–24510)	315.11 (270.45–360.97)	0.17 (0.16–0.18)
Republic of Guinea-Bissau	1417 (1197–1636)	298.26 (253.73–347.69)	3123 (2644–3608)	314.58 (270.12–362.29)	0.15 (0.14–0.16)
Republic of Guyana	1524 (1320–1737)	343.4 (297.54–394.62)	2700 (2307–3110)	372.81 (322.16–427.05)	0.27 (0.25–0.29)
Republic of Haiti	12204 (10542–13996)	321.15 (277.19–367.18)	31043 (26669–35514)	339.2 (294.21–386.43)	0.2 (0.19–0.21)
Republic of Honduras	7941 (6773–9129)	336.35 (288.67–388.78)	27065 (23336–30822)	367.99 (316.72–420.41)	0.29 (0.28–0.31)
Republic of Iceland	881 (769–1013)	336.87 (292.31–386.55)	1781 (1549–2038)	361.71 (314.16–412.49)	0.21 (0.2–0.22)
Republic of India	1723865 (1477512–1963448)	302.9 (261.25–344.42)	4416602 (3806875–5017935)	331.38 (286.04–376.48)	0.3 (0.28–0.31)
Republic of Indonesia	299769 (254846–345015)	247.63 (212.7–282.94)	807366 (686278–936248)	273.05 (234.69–312)	0.36 (0.35–0.37)
Republic of Iraq	27711 (23676–31513)	305.98 (261.54–350.96)	99904 (84429–115797)	323.86 (275.22–370.11)	0.2 (0.18–0.21)
Republic of Italy	265489 (230968–305715)	333.01 (288.6–379.73)	379595 (332840–437987)	351.76 (306.14–401.52)	0.13 (0.12–0.15)
Republic of Kazakhstan	31618 (27246–36362)	233.2 (201.32–268.54)	49374 (42028–56635)	249.14 (214.38–285.64)	0.21 (0.2–0.22)
Republic of Kenya	27982 (23900–32099)	287.81 (247.39–331.66)	91278 (77743–105051)	311.51 (267.23–358.61)	0.27 (0.24–0.29)
Republic of Kiribati	149 (127–171)	329.72 (284.92–379.58)	330 (280–380)	360.17 (307.5–414.32)	0.25 (0.2–0.3)
Republic of Korea	175124 (150561–200592)	480.61 (416.52–552.19)	433774 (378392–498229)	491.74 (427.19–560.51)	0.21 (0.1–0.32)
Republic of Latvia	9774 (8314–11375)	283.84 (242.68–325.97)	9390 (8084–10966)	302.49 (259.76–345.39)	0.23 (0.21–0.24)
Republic of Liberia	3907 (3382–4463)	306.96 (265.25–354.38)	10220 (8677–11946)	332.59 (285.96–382.71)	0.33 (0.29–0.36)
Republic of Lithuania	12365 (10650–14311)	281.59 (243.45–322.43)	13636 (11779–15990)	299.75 (258.41–345.15)	0.22 (0.21–0.24)
Republic of Madagascar	16334 (13891–18759)	277.21 (237.35–318.95)	44510 (37631–51409)	288.87 (247.56–333.28)	0.15 (0.14–0.17)
Republic of Malawi	13122 (11210–15086)	284.5 (244.85–324.23)	28840 (24494–33110)	304.18 (260.52–349.83)	0.25 (0.24–0.26)
Republic of Maldives	273 (228–317)	242.63 (206.95–278.22)	1370 (1159–1584)	271.95 (232.49–315.36)	0.46 (0.43–0.49)
Republic of Mali	13799 (11711–15932)	291.93 (251.12–336.73)	34432 (29545–39920)	311.58 (267.02–358.42)	0.21 (0.21–0.22)
Republic of Malta	1417 (1232–1631)	329.49 (285.31–380.29)	2630 (2309–3012)	354.38 (310.46–404.66)	0.18 (0.15–0.21)
Republic of Mauritius	2214 (1906–2545)	264.51 (228.41–305.68)	5198 (4431–6028)	292.51 (250.77–336.87)	0.36 (0.34–0.37)
Republic of Moldova	12497 (10698–14515)	272.38 (235.06–314.72)	16544 (14272–19234)	297.29 (256.34–342.44)	0.34 (0.3–0.37)
Republic of Mozambique	20448 (17455–23329)	280 (240.14–322.66)	43137 (36697–49372)	298.98 (256.13–346.19)	0.23 (0.23–0.24)
Republic of Namibia	2215 (1905–2545)	295.16 (254.62–338.98)	5396 (4600–6268)	317.84 (271.23–369.61)	0.22 (0.21–0.23)
Republic of Nauru	20 (17–24)	329.57 (284.12–380.75)	28 (24–32)	370.9 (319.45–426.67)	0.33 (0.31–0.35)
Republic of Nicaragua	6133 (5273–6960)	341.27 (294.12–388)	20722 (17775–23742)	373.74 (321.5–430.12)	0.3 (0.27–0.32)
Republic of Niue	7 (6–8)	343.63 (297.96–396.28)	8 (7–9)	382.24 (329.74–441.54)	0.33 (0.29–0.37)
Republic of Palau	39 (34–45)	345.07 (294.59–396.44)	101 (86–120)	376.41 (323.09–435.76)	0.25 (0.22–0.29)
Republic of Panama	5418 (4686–6160)	332.06 (286.92–379.71)	16487 (14266–18841)	372.48 (322.36–424.82)	0.35 (0.34–0.37)
Republic of Paraguay	8468 (7279–9612)	345.61 (296.47–393.69)	23633 (20363–26969)	371.56 (320.57–424.14)	0.25 (0.24–0.26)
Republic of Peru	46348 (39804–52965)	344.36 (297.75–395.18)	135754 (116593–156640)	383.76 (329.78–442.03)	0.38 (0.37–0.39)
Republic of Poland	113616 (97853–131066)	263.46 (227.16–303.92)	174134 (151398–201862)	284.62 (245.14–327.01)	0.25 (0.25–0.26)
Republic of Rwanda	9474 (8114–10868)	283.67 (243.68–325.71)	24192 (20590–27821)	301.71 (258.74–347.37)	0.22 (0.21–0.23)
Republic of San Marino	104 (91–120)	338.5 (294.88–386.54)	206 (179–238)	363.14 (313.94–414.45)	0.19 (0.17–0.21)
Republic of Senegal	11394 (9743–13116)	305.86 (261.6–354.95)	30251 (25636–34872)	324.94 (277.71–375.05)	0.17 (0.15–0.18)
Republic of Serbia	31477 (26716–36713)	264.01 (225.94–302.23)	39275 (33813–45400)	280.95 (240.51–322.92)	0.23 (0.22–0.24)
Republic of Seychelles	148 (128–169)	271.97 (235.02–313.05)	382 (324–442)	292.1 (250.08–336.64)	0.24 (0.23–0.26)
Republic of Sierra Leone	6699 (5770–7614)	295.66 (253.38–338.77)	14924 (12772–17111)	314.99 (271.78–361.27)	0.19 (0.18–0.2)
Republic of Singapore	12026 (10304–13776)	446.98 (385.51–511.34)	41341 (35907–47285)	467.22 (405.99–533.81)	0.09 (0.07–0.11)
Republic of Slovenia	6409 (5534–7439)	265.44 (228.63–306.55)	9986 (8633–11637)	282.27 (242.27–327.33)	0.22 (0.21–0.23)
Republic of South Africa	74306 (63379–85286)	320.58 (274.25–369.21)	181939 (156284–208194)	343.76 (295.83–394.13)	0.24 (0.24–0.25)
Republic of South Sudan	8078 (6939–9349)	280.88 (240.97–324.96)	15818 (13278–18281)	299.05 (256.5–342.94)	0.22 (0.21–0.23)
Republic of Sudan	28618 (24652–32623)	269.7 (231.77–307.97)	78318 (67224–90415)	310.39 (267.81–360.14)	0.47 (0.45–0.5)
Republic of Suriname	990 (849–1141)	350.6 (301.7–402.91)	2549 (2180–2957)	381.91 (328.92–438.35)	0.3 (0.29–0.31)
Republic of Tajikistan	6133 (5271–7027)	211 (181.5–241.1)	15836 (13484–18307)	218.48 (189.9–250.18)	0.11 (0.09–0.13)
Republic of the Congo	3677 (3112–4236)	294.94 (252.24–338.88)	11717 (9970–13588)	314.59 (271.69–363.49)	0.22 (0.21–0.22)
Republic of the Gambia	1303 (1107–1494)	299.05 (256.02–346.86)	3973 (3370–4554)	325.63 (278.32–375.12)	0.27 (0.25–0.28)
Republic of the Marshall Islands	63 (54–72)	311.22 (267.81–355.82)	163 (138–188)	345.16 (296.04–394.62)	0.31 (0.28–0.33)
Republic of the Niger	10376 (8821–11914)	290.3 (249.18–333.16)	31537 (26848–36392)	306.87 (264.61–354.24)	0.17 (0.16–0.18)
Republic of the Philippines	81177 (68633–93294)	222.37 (190.84–255.09)	242884 (205674–280637)	251.52 (216.08–288.24)	0.42 (0.39–0.45)
Republic of the Union of Myanmar	65107 (55500–74676)	246.19 (212.07–283.17)	150459 (128999–174064)	272.5 (234.6–313.41)	0.39 (0.37–0.4)
Republic of Trinidad and Tobago	3232 (2793–3666)	359.52 (308.69–410.83)	7315 (6338–8499)	386.59 (335.82–442.11)	0.29 (0.27–0.31)
Republic of Tunisia	16117 (13924–18517)	290.21 (250.92–331.91)	45562 (39223–52163)	321.5 (278.2–367.5)	0.34 (0.33–0.35)
Republic of Turkey	123161 (104949–142079)	309.08 (267.55–355.91)	339742 (290165–391937)	342.72 (294.38–393.33)	0.35 (0.33–0.37)
Republic of Uganda	21044 (17916–24075)	279.39 (240.24–320.89)	57144 (48496–65544)	300.15 (257.86–347.36)	0.26 (0.25–0.26)
Republic of Uzbekistan	26663 (22906–30751)	220.6 (190.52–252.82)	74265 (63347–86037)	237.73 (203.94–274.72)	0.25 (0.23–0.27)
Republic of Vanuatu	255 (216–293)	314.4 (266.57–361.08)	773 (655–899)	346.3 (298.14–399.67)	0.3 (0.29–0.32)
Republic of Yemen	15714 (13445–18094)	263.54 (226.65–302.44)	52993 (45210–60683)	287.92 (246.68–331.92)	0.31 (0.3–0.32)
Republic of Zambia	10043 (8566–11545)	288.03 (248.31–331.89)	28993 (24633–33446)	305.74 (261.9–352.6)	0.2 (0.18–0.22)
Republic of Zimbabwe	14346 (12302–16455)	297.96 (256.25–344.7)	27675 (23559–31925)	309.77 (265.12–358.16)	0.08 (0.06–0.11)
Romania	73125 (62201–86273)	260.31 (222.96–302.07)	87688 (75710–100657)	280.65 (241–321.27)	0.27 (0.26–0.27)
Russian Federation	499866 (427543–578018)	277.26 (238.61–319.18)	663536 (571706–769157)	300.35 (258.12–344.46)	0.29 (0.27–0.3)
Saint Kitts and Nevis	114 (99–130)	359.25 (307.04–414.37)	309 (263–358)	387.77 (334.65–441.85)	0.25 (0.24–0.26)
Saint Lucia	307 (267–351)	350.14 (303.26–400.98)	927 (796–1074)	380.05 (327.06–435.93)	0.25 (0.23–0.27)
Saint Vincent and the Grenadines	238 (207–274)	343.8 (296.04–395.82)	538 (461–627)	375.04 (322.48–431.65)	0.32 (0.3–0.33)
Slovak Republic	15502 (13435–17823)	266.14 (228.74–306.2)	24243 (20800–28253)	282.27 (241.52–328.41)	0.17 (0.16–0.19)
Socialist Republic of Viet Nam	101939 (87431–116993)	245.97 (210.17–281.79)	295456 (250554–341989)	263.31 (225.17–303.44)	0.26 (0.25–0.28)
Solomon Islands	535 (453–623)	308.69 (264.18–357.18)	1602 (1356–1890)	341.61 (292.13–391.5)	0.29 (0.25–0.34)
State of Eritrea	4294 (3622–4986)	273 (236.27–315.04)	10964 (9328–12626)	287.53 (246.84–329.86)	0.17 (0.16–0.18)
State of Israel	15259 (13306–17554)	331.99 (286.14–381.98)	38576 (33465–44150)	355.97 (309.05–408.55)	0.17 (0.15–0.2)
State of Kuwait	3049 (2585–3509)	308.26 (263.91–354.69)	17412 (14594–20128)	347.46 (299.8–397.15)	0.44 (0.42–0.45)
State of Libya	6677 (5670–7694)	302.97 (258.56–349.68)	23239 (19603–26846)	330.44 (283.41–378.05)	0.3 (0.28–0.31)
State of Qatar	814 (677–953)	309.82 (267.27–357.14)	8832 (7426–10302)	343.01 (294.2–396.91)	0.29 (0.27–0.31)
Sultanate of Oman	2665 (2277–3064)	276.03 (237.17–317.58)	11710 (9903–13473)	327.76 (282.29–379.71)	0.6 (0.58–0.62)
Swiss Confederation	29917 (25920–34432)	329.14 (282.45–380.2)	49808 (43142–57702)	346.06 (298.82–398.71)	0.13 (0.12–0.15)
Syrian Arab Republic	17878 (15307–20448)	292.6 (251.15–335.16)	50241 (42723–58153)	320.6 (274.64–364.04)	0.28 (0.26–0.3)
Taiwan (Province of China)	65849 (56320–76186)	371.4 (319.54–431.4)	155802 (133559–179740)	418.79 (361.81–477.52)	0.46 (0.44–0.49)
Togolese Republic	4696 (3985–5460)	298.99 (257.15–347.22)	16507 (14016–18943)	320.26 (275.08–367.1)	0.2 (0.19–0.22)
Tokelau	4 (4–5)	330.37 (284.48–382.44)	5 (5–6)	368.59 (316.91–422.22)	0.34 (0.31–0.37)
Turkmenistan	4671 (3987–5379)	223.75 (192.65–258.01)	11259 (9509–13163)	241.92 (207.8–277.27)	0.27 (0.26–0.28)
Tuvalu	25 (21–29)	329.8 (283.85–380.12)	40 (34–46)	358.25 (308.78–411.47)	0.23 (0.19–0.27)
Ukraine	194133 (165380–228725)	280.6 (240.54–326.1)	204934 (175903–236527)	292.95 (249.91–335.84)	0.16 (0.15–0.18)
Union of the Comoros	674 (577–779)	287.19 (248.74–332.29)	1770 (1511–2039)	303.72 (260.3–350.03)	0.19 (0.18–0.19)
United Arab Emirates	2910 (2436–3401)	292.99 (250.04–341.49)	41219 (34417–48957)	329.15 (283.49–377.72)	0.4 (0.39–0.41)
United Kingdom of Great Britain and Northern Ireland	270669 (236137–311172)	355.05 (308.14–405.62)	396116 (346846–456600)	383.77 (333.09–436.78)	0.26 (0.2–0.33)
United Mexican States	178669 (153269–204203)	363.55 (313.1–416.72)	534524 (458306–612961)	390.83 (336.89–447.09)	0.24 (0.23–0.24)
United Republic of Tanzania	36222 (30892–41616)	287.86 (246.91–331.04)	99374 (85167–115443)	308.44 (266.52–356.13)	0.24 (0.23–0.25)
United States of America	1093868 (959538–1243508)	384.67 (332.96–439.61)	1960492 (1707253–2261850)	406.51 (350.48–464.22)	−0.07 (−0.24–0.11)
United States Virgin Islands	384 (330–441)	378.48 (326.94–432.59)	600 (519–700)	405.65 (351.34–466.11)	0.24 (0.21–0.26)

**Table 2:** The number of prevalence cases and the age-standardized prevalence rate attributable to osteoarthritis knee in 1990 and 2021, and its trends from 1990 to 2021 globally

	Number of prevalence cases (95% UI) in 1990	The age-standardized prevalence rate/100000 (95% UI) in 1990	Number of prevalence cases (95% UI) in 2021	The age-standardized prevalence rate/100000 (95% UI) in 2021	EAPC (95% CI)
Global	159798909 (137277437–182882554)	3964.75 (3411.86–4536.4)	374738744 (321858982–428353220)	4294.27 (3695.04–4910.76)	0.33 (0.3–0.35)
Sex					
Female	98498538 (84692265–112495598)	4613.23 (3972.13–5267.02)	230189286 (198051498–262858482)	5029.51 (4331.89–5738.43)	0.36 (0.33–0.4)
Male	61300372 (52267191–70269784)	3224.33 (2771.36–3685.31)	144549459 (123542116–165818687)	3483.37 (2991.52–3989.67)	0.3 (0.27–0.32)
Age					
< 5 years	0 (0–0)	0 (0–0)	0 (0–0)	0 (0–0)	0 (0–0)
5–9 years	0 (0–0)	0 (0–0)	0 (0–0)	0 (0–0)	0 (0–0)
10–14 years	0 (0–0)	0 (0–0)	0 (0–0)	0 (0–0)	0 (0–0)
15–19 years	0 (0–0)	0 (0–0)	0 (0–0)	0 (0–0)	0 (0–0)
20–24 years	0 (0–0)	0 (0–0)	0 (0–0)	0 (0–0)	0 (0–0)
25–29 years	0 (0–0)	0 (0–0)	0 (0–0)	0 (0–0)	0 (0–0)
30–34 years	391331 (285246–526393)	101.53 (74.01–136.58)	664479 (483746–890862)	109.93 (80.03–147.38)	0.4 (0.29–0.5)
35–39 years	3437597 (2483463–4608307)	975.91 (705.04–1308.27)	5841634 (4231517–7801647)	1041.54 (754.46–1391)	0.42 (0.28–0.57)
40–44 years	8158515 (6352088–10457870)	2847.83 (2217.28–3650.45)	15196205 (11810709–19415585)	3037.72 (2360.96–3881.17)	0.46 (0.31–0.61)
45–49 years	13525081 (10953998–16262928)	5824.87 (4717.58–7003.98)	30059813 (24323836–36045160)	6348.37 (5136.98–7612.42)	0.49 (0.39–0.58)
50–54 years	19917034 (15455198–24946924)	9369.57 (7270.59–11735.78)	46619708 (36141664–58291310)	10478.15 (8123.13–13101.44)	0.47 (0.42–0.51)
55–59 years	23544190 (18973462–28153011)	12712.85 (10244.85–15201.41)	55621239 (45079916–66454322)	14055.42 (11391.64–16792.93)	0.38 (0.34–0.43)
60–64 years	24885705 (20354932–29857002)	15494.57 (12673.57–18589.84)	53626680 (43844245–64102640)	16755.83 (13699.28–20029.08)	0.35 (0.32–0.39)
65–69 years	22480858 (18685104–27196812)	18187 (15116.24–22002.21)	53790239 (44848663–64334842)	19500.37 (16258.81–23323.06)	0.28 (0.26–0.31)
70–74 years	17325613 (14578086–20641994)	20464.66 (17219.33–24381.9)	44819022 (37668998–53407317)	21773.8 (18300.2–25946.13)	0.2 (0.17–0.22)
75–79 years	13598101 (11524298–16146012)	22090.79 (18721.8–26230)	30826349 (26164394–36565377)	23373.76 (19838.88–27725.32)	0.14 (0.11–0.17)
80–84 years	8071703 (6853441–9564994)	22816.94 (19373.18–27038.14)	21264233 (18065994–25136547)	24278.93 (20627.27–28700.24)	0.16 (0.14–0.18)
85–89 years	3374043 (2857031–3970583)	22328.28 (18906.87–26275.98)	11092546 (9414234–13026388)	24260.96 (20590.26–28490.55)	0.24 (0.22–0.26)
90–94 years	895554 (765761–1051791)	20898.84 (17869.96–24544.8)	4130842 (3529799–4858914)	23091.04 (19731.26–27160.9)	0.34 (0.32–0.36)
95 + years	193585 (165117–228449)	19014.55 (16218.35–22439.04)	1185755 (1012252–1397534)	21755.74 (18572.39–25641.37)	0.45 (0.44–0.47)
SDI region					
High-middle SDI	37990614 (32600684–43592484)	3772.7 (3248.87–4327.23)	85016578 (72744239–97535184)	4295.79 (3685.79–4926.17)	0.53 (0.48–0.59)
High SDI	47279525 (41017926–53920968)	4364 (3780.18–4972.97)	90099016 (78371034–102834145)	4656.7 (4033.58–5313.55)	0.16 (0.12–0.2)
Low-middle SDI	21885269 (18774477–25081455)	3427.13 (2946.77–3915.09)	56770333 (48884057–64923405)	3790.26 (3258.77–4325.52)	0.35 (0.34–0.36)
Low SDI	7844041 (6696613–8986414)	3324.31 (2859.98–3803.29)	19146655 (16425264–21911203)	3545.62 (3048.24–4056.6)	0.22 (0.21–0.23)
Middle SDI	44648677 (37987635–51370949)	4102.09 (3527.55–4699.44)	123424845 (105700584–141777409)	4396.08 (3789.23–5031.25)	0.38 (0.32–0.45)
GBD region					
Advanced Health System	65305372 (56685566–74463171)	4037.25 (3500.74–4603)	116498390 (101184512–133060220)	4374.71 (3801.19–4986.65)	0.21 (0.19–0.24)
Africa	9906042 (8448704–11364822)	3379.85 (2907.05–3890.81)	25784850 (21955703–29645272)	3684.54 (3161.33–4225.44)	0.28 (0.27–0.29)
African Region	7808546 (6655092–8973974)	3390.83 (2915.15–3904.19)	19997319 (17003121–22980104)	3662.66 (3142.98–4208.47)	0.25 (0.24–0.27)
America	26057246 (22578537–29783769)	4302.29 (3725.27–4912.8)	60846505 (52655822–69445473)	4589.85 (3973.75–5245.26)	0.09 (−0.01–0.18)
Andean Latin America	853460 (738034–976452)	4070.11 (3516.08–4660.84)	2758218 (2370967–3163149)	4588.33 (3950.43–5244.03)	0.42 (0.41–0.43)
Asia	86769991 (73959355–99767449)	4095.48 (3518.19–4688.19)	229879993 (197005067–263567808)	4405.67 (3785.02–5041.46)	0.38 (0.32–0.44)
Australasia	993266 (867688–1128276)	4288.08 (3746.3–4863.21)	2389034 (2066114–2736476)	4808.67 (4147.42–5488.06)	0.36 (0.34–0.39)
Basic Health System	63686000 (54078323–73555290)	4115.15 (3530.66–4721.18)	174892775 (149017807–201139643)	4471.28 (3829.87–5127.6)	0.45 (0.37–0.52)
Caribbean	1066131 (923195–1224744)	4080.07 (3540.14–4677.22)	2403792 (2070228–2740275)	4454.36 (3840.02–5084.92)	0.32 (0.3–0.33)
Central Africa	993997 (845757–1144487)	3376.53 (2898.52–3877.76)	2547705 (2183211–2920147)	3532.22 (3041.31–4039.09)	0.11 (0.1–0.13)
Central Asia	1192276 (1022977–1370278)	2533.07 (2186.47–2911.04)	2301085 (1969099–2659417)	2700.22 (2336.33–3105.34)	0.21 (0.2–0.23)
Central Europe	4512504 (3866745–5163412)	3005.2 (2586.99–3442.37)	6774519 (5844666–7842619)	3230.75 (2788.51–3718.9)	0.25 (0.24–0.25)
Central Latin America	3520770 (3020725–4018304)	4114.19 (3551.89–4687.11)	11498876 (9894419–13149726)	4492.79 (3881.3–5124.51)	0.28 (0.28–0.29)
Central Sub-Saharan Africa	776748 (661806–895686)	3288.2 (2816.19–3766.21)	2053774 (1756352–2357159)	3433.3 (2947.25–3926.7)	0.11 (0.09–0.13)
Commonwealth High Income	5580556 (4877207–6402692)	3826.21 (3338.47–4377.09)	10349287 (8985379–11782277)	4123.24 (3584.32–4696.31)	0.23 (0.2–0.26)
Commonwealth Low Income	2966715 (2542635–3399435)	3271.22 (2807.32–3734.88)	8609249 (7382907–9834681)	3542.43 (3038.9–4042.92)	0.29 (0.27–0.3)
Commonwealth Middle Income	22885946 (19524557–26235340)	3461.54 (2975.35–3951.35)	62665533 (53801003–71495374)	3828.59 (3293.97–4371.41)	0.35 (0.34–0.36)
East Asia	42602441 (35969751–49229166)	4662.94 (3992.86–5359.08)	113375622 (96002567–130995916)	5016.78 (4267.39–5758.88)	0.49 (0.39–0.6)
East Asia & Pacific—WB	62265464 (53001110–71765518)	4465.08 (3839.3–5117.97)	160386360 (137071674–184229772)	4753.76 (4077.46–5454.48)	0.4 (0.32–0.48)
Eastern Africa	2301632 (1965217–2645093)	3192.03 (2749.02–3678.98)	5916261 (5046103–6792506)	3462.43 (2978.45–3964.89)	0.29 (0.27–0.3)
Eastern Europe	8853765 (7574166–10111455)	3154.42 (2713.05–3603.85)	11762775 (10059205–13517483)	3409.25 (2919.69–3923.46)	0.28 (0.27–0.3)
Eastern Mediterranean Region	5916541 (5047459–6811401)	3155.95 (2706.57–3626.83)	17691783 (15070494–20441626)	3591.13 (3060.07–4124.27)	0.43 (0.43–0.44)
Eastern Sub-Saharan Africa	2491439 (2121796–2866357)	3216.44 (2769.51–3707.07)	6218769 (5299896–7142986)	3446.18 (2959.9–3965.07)	0.24 (0.23–0.26)
Europe	36739138 (31816861–42027040)	3570.08 (3096.03–4062.39)	57600144 (49888102–65867506)	3841.37 (3333.19–4385.46)	0.25 (0.24–0.26)
Europe & Central Asia—WB	37471561 (32451003–42872267)	3540.01 (3071.19–4029.07)	59121160 (51212706–67607747)	3795.13 (3293.33–4333.63)	0.24 (0.22–0.25)
European Region	37727593 (32673796–43160155)	3540.64 (3071.57–4029.68)	59749799 (51750811–68336689)	3796.75 (3294.73–4335.34)	0.24 (0.22–0.25)
High-income Asia Pacific	11135071 (9603769–12692418)	5416.8 (4683–6163.06)	22540484 (19570221–25550915)	5573.73 (4841.29–6334.33)	0.15 (0.11–0.19)
High-income North America	15143603 (13138164–17316618)	4500.08 (3894.98–5121.91)	29008914 (25038463–33373205)	4709.03 (4074.67–5394.69)	−0.11 (−0.31–0.08)
Latin America & Caribbean—WB	11082268 (9563542–12672100)	4072.15 (3536.65–4644.46)	32164884 (27766666–36718837)	4500.99 (3896.47–5132.59)	0.34 (0.32–0.35)
Limited Health System	28619039 (24476781–32760680)	3431.3 (2950.99–3916.69)	78382206 (67365878–89475380)	3778.54 (3251.13–4311.13)	0.33 (0.33–0.34)
Middle East & North Africa—WB	4305661 (3687839–4951983)	3376.6 (2907.45–3868.02)	14065132 (12046586–16182926)	3783.43 (3241.27–4335.85)	0.36 (0.34–0.37)
Minimal Health System	2037715 (1737339–2340100)	3276.56 (2820.23–3756.05)	4684055 (3986260–5374273)	3478.52 (2989.62–3995.95)	0.19 (0.19–0.2)
North Africa and Middle East	5916998 (5065219–6807128)	3378.07 (2909.97–3880.84)	18591752 (15988433–21426733)	3810.43 (3275.1–4375)	0.39 (0.38–0.4)
North America	15145184 (13139498–17318472)	4500.31 (3895.18–5122.17)	29012433 (25041268–33377198)	4709.27 (4074.86–5394.98)	−0.11 (−0.31–0.08)
Northern Africa	2177595 (1860226–2512446)	3387.51 (2906.21–3877.01)	6320173 (5410856–7293074)	3821.4 (3265.42–4402.56)	0.37 (0.36–0.39)
Oceania	119873 (101127–139191)	3730.88 (3201.2–4301.16)	338675 (285507–391076)	4025.21 (3433.77–4602.96)	0.2 (0.17–0.23)
Region of the Americas	26057246 (22578537–29783769)	4302.29 (3725.27–4912.8)	60846505 (52655822–69445473)	4589.85 (3973.75–5245.26)	0.09 (−0.01–0.18)
South-East Asia Region	25429102 (21732706–29098768)	3396.47 (2924.04–3866.67)	71330946 (61310728–81386961)	3762.09 (3242.66–4286.08)	0.36 (0.35–0.37)
South Asia	21001741 (17861857–24088981)	3441.76 (2953.66–3924.8)	58791056 (50326960–67052871)	3818.04 (3282.09–4349.41)	0.36 (0.36–0.37)
South Asia—WB	21559373 (18341436–24718901)	3427.76 (2941.17–3909.8)	60069308 (51439151–68506107)	3804.22 (3271–4333.26)	0.37 (0.36–0.38)
Southeast Asia	7773368 (6600060–8940996)	2885.33 (2480.85–3300.1)	22706789 (19313885–26216287)	3238.01 (2773.46–3719.63)	0.42 (0.41–0.43)
Southern Africa	1564495 (1330614–1795714)	3477.62 (2987.27–3996.79)	3772316 (3216170–4322527)	3750.45 (3225.44–4307.24)	0.26 (0.25–0.26)
Southern Latin America	1920099 (1659204–2191582)	4128.09 (3574.76–4711.31)	3942908 (3422500–4496778)	4617.51 (4009.6–5266.68)	0.35 (0.32–0.39)
Southern Sub-Saharan Africa	1002298 (855477–1155712)	3626.52 (3109.81–4181.9)	2363886 (2022846–2722791)	3913.26 (3363.99–4509.41)	0.26 (0.25–0.26)
Sub-Saharan Africa—WB	7761104 (6614068–8915079)	3379.4 (2905.97–3891.06)	19530521 (16612066–22448880)	3645.47 (3128.98–4187.47)	0.25 (0.23–0.26)
Tropical Latin America	3760894 (3229593–4307856)	4003.22 (3445.06–4588.46)	11648775 (10018943–13328778)	4455.51 (3838.66–5095.51)	0.38 (0.36–0.39)
Western Africa	2868322 (2451542–3292142)	3484.12 (2985.36–4005.96)	7228394 (6132099–8314156)	3789.78 (3239.68–4363.81)	0.27 (0.22–0.32)
Western Europe	21982300 (19056229–25147170)	3934.19 (3410.31–4494.01)	35172347 (30617674–40161660)	4169.47 (3611.88–4769.26)	0.18 (0.16–0.2)
Western Pacific Region	55773332 (47489852–64205795)	4673.75 (4019.36–5358.71)	142323912 (121633628–163550647)	4959.19 (4250.18–5692.17)	0.4 (0.31–0.49)
Western Sub-Saharan Africa	3179864 (2716760–3652589)	3496.16 (2995.24–4022.03)	8096695 (6882245–9292419)	3801.12 (3250.23–4369.9)	0.26 (0.22–0.31)
World Bank High Income	54952187 (47692143–62729497)	4251.48 (3683.62–4849.06)	100699307 (87605316–114913055)	4554.77 (3955.92–5196.78)	0.16 (0.12–0.2)
World Bank Low Income	4841171 (4127146–5572339)	3388.57 (2913.95–3885.69)	11455280 (9773118–13163554)	3601.53 (3089.57–4139.13)	0.21 (0.2–0.22)
World Bank Lower Middle Income	37021020 (31722382–42375711)	3294.63 (2829.19–3766.85)	99884805 (85796610–114230162)	3644.2 (3130.5–4153.28)	0.35 (0.34–0.36)
World Bank Upper Middle Income	62833052 (53481894–72386852)	4188.92 (3597.07–4804.69)	162416619 (138645122–186589720)	4639.72 (3974.84–5320.45)	0.52 (0.44–0.59)
Country					
American Samoa	1082 (911–1250)	4394.39 (3743.9–5036.7)	2492 (2123–2884)	4794.96 (4115.6–5519.1)	0.23 (0.15–0.3)
Antigua and Barbuda	2086 (1804–2393)	4087.85 (3521.38–4684.26)	4929 (4228–5633)	4425.15 (3806.19–5058.23)	0.25 (0.25–0.26)
Arab Republic of Egypt	999604 (848502–1165571)	3457.09 (2971.6–3969.69)	2702515 (2292126–3154778)	3883.05 (3322.01–4491.44)	0.32 (0.29–0.35)
Argentine Republic	1329777 (1144882–1523632)	4094.38 (3531.14–4675.36)	2506329 (2162070–2852391)	4569.29 (3940.95–5225.46)	0.35 (0.31–0.39)
Australia	831099 (721746–942383)	4301.83 (3760–4860.33)	2016533 (1745326–2312719)	4838.82 (4179.84–5526.62)	0.37 (0.34–0.39)
Barbados	11767 (10019–13582)	4329.32 (3701.18–4980.22)	23281 (19855–26498)	4620.98 (3946.01–5269.45)	0.21 (0.2–0.23)
Belize	3834 (3295–4392)	4128.19 (3532.85–4739.46)	14567 (12525–16855)	4621.04 (3999.59–5297.33)	0.35 (0.29–0.4)
Bermuda	2893 (2484–3293)	4572.76 (3936.94–5200.28)	6120 (5223–7046)	4798.09 (4091.41–5509.6)	0.15 (0.14–0.16)
Bolivarian Republic of Venezuela	411280 (353058–471535)	4130.66 (3543.38–4739.14)	1382427 (1185082–1587522)	4467.86 (3847.08–5127.32)	0.26 (0.25–0.27)
Bosnia and Herzegovina	122969 (104446–142468)	2878.62 (2454.01–3322.66)	188335 (161913–216493)	3128.74 (2693.56–3580.86)	0.29 (0.26–0.32)
Brunei Darussalam	5804 (5033–6577)	5364.25 (4677.82–6069.33)	22212 (19106–25249)	5824.76 (5024.72–6623.85)	0.27 (0.26–0.29)
Burkina Faso	151875 (129801–173958)	3394.55 (2920.73–3893.13)	364727 (310444–421219)	3723.39 (3164.93–4270.1)	0.29 (0.29–0.3)
Canada	763567 (665048–872710)	2389.67 (2079.91–2727.73)	1817661 (1572491–2082024)	2722.94 (2357.99–3111.62)	0.26 (0.17–0.35)
Central African Republic	38317 (32303–44293)	3199.62 (2730.82–3666.4)	80806 (68444–93300)	3324.43 (2842.53–3830.04)	0.13 (0.12–0.14)
Commonwealth of Dominica	2416 (2076–2768)	4196.95 (3615.72–4815.81)	3821 (3272–4371)	4480.28 (3850.37–5117.2)	0.2 (0.17–0.22)
Commonwealth of the Bahamas	6802 (5837–7837)	4317.12 (3712.6–4970.55)	19801 (16847–22654)	4609.41 (3940.04–5254.46)	0.21 (0.19–0.23)
Cook Islands	554 (469–639)	4198.12 (3598.36–4814.78)	1244 (1061–1434)	4844.99 (4139.89–5580.52)	0.43 (0.38–0.47)
Czech Republic	411217 (349506–475727)	3034.57 (2583.68–3487.64)	648726 (562837–742368)	3247.81 (2808.98–3716.97)	0.22 (0.21–0.23)
Democratic People's Republic of Korea	799391 (679386–927274)	4555.29 (3917.5–5249.59)	1631136 (1388699–1880963)	4751.33 (4072.88–5461.25)	0.15 (0.14–0.17)
Democratic Republic of Sao Tome and Principe	2343 (1994–2705)	3593.16 (3077.25–4145.25)	4777 (4076–5501)	3932.58 (3369.85–4520.61)	0.29 (0.29–0.3)
Democratic Republic of the Congo	542601 (461159–627481)	3305.79 (2828.21–3807.15)	1344398 (1147927–1542546)	3382.47 (2897.15–3861.86)	0.03 (0–0.05)
Democratic Republic of Timor-Leste	9096 (7699–10548)	2808.1 (2410.67–3230.36)	26093 (22373–30144)	2972.71 (2557.3–3426.43)	0.21 (0.2–0.23)
Democratic Socialist Republic of Sri Lanka	336455 (286663–388295)	2930.89 (2507.46–3385.08)	897956 (765420–1025712)	3262.98 (2799.04–3714.06)	0.39 (0.38–0.4)
Dominican Republic	150851 (130194–173912)	3966.5 (3421.96–4552.88)	450799 (388215–516222)	4447.93 (3835.78–5095.94)	0.41 (0.39–0.42)
Eastern Republic of Uruguay	158091 (136165–179919)	4133.63 (3568.7–4712.42)	238369 (208096–274000)	4646.43 (4035.17–5333.8)	0.37 (0.34–0.4)
Federal Democratic Republic of Ethiopia	646084 (549386–747051)	3145.96 (2715.19–3634.65)	1528816 (1310890–1762268)	3355.66 (2881.74–3875.43)	0.23 (0.21–0.25)
Federal Democratic Republic of Nepal	331909 (283442–376808)	3316.5 (2847.56–3760.78)	912049 (783876–1044685)	3762.47 (3255.34–4301.22)	0.45 (0.42–0.47)
Federal Republic of Germany	4897596 (4248140–5602423)	3988.98 (3461.41–4566.2)	7294669 (6310536–8362362)	4185.74 (3634.76–4827.33)	0.15 (0.14–0.16)
Federal Republic of Nigeria	1598941 (1363956–1833070)	3506.89 (3006.89–4024.57)	3796910 (3214277–4359135)	3807.39 (3253.69–4369.2)	0.26 (0.17–0.35)
Federal Republic of Somalia	83803 (71238–96684)	3206.89 (2738.49–3716.77)	222003 (188308–255289)	3319.84 (2850.49–3818)	0.14 (0.13–0.15)
Federated States of Micronesia	1962 (1682–2255)	3993.07 (3452.23–4590.43)	3622 (3047–4189)	4425.29 (3769.33–5052.05)	0.28 (0.22–0.34)
Federative Republic of Brazil	3669907 (3151434–4202859)	4002.44 (3443.05–4588.75)	11386850 (9787425–13035647)	4456.95 (3837.48–5098.63)	0.38 (0.36–0.39)
French Republic	3047416 (2644900–3479039)	3843.85 (3331.7–4356.53)	5118522 (4460457–5923102)	4100.74 (3547.57–4689.32)	0.2 (0.19–0.22)
Gabonese Republic	19897 (16867–22919)	3457.5 (2948.04–3982.4)	44078 (37661–51154)	3905.88 (3372.92–4537.44)	0.37 (0.36–0.39)
Georgia	165307 (140471–191612)	2624.23 (2241.46–3023.06)	157520 (134139–183640)	2722.08 (2322.61–3165.66)	0.13 (0.12–0.15)
Grand Duchy of Luxembourg	21172 (18269–24438)	3944.75 (3414.39–4540.97)	42307 (36949–48000)	4136.34 (3612.23–4691.6)	0.15 (0.13–0.16)
Greenland	965 (825–1111)	2645.9 (2294.84–3060.27)	2146 (1818–2486)	2947.19 (2534.73–3400.08)	0.35 (0.32–0.38)
Grenada	2717 (2341–3122)	4007.18 (3436.34–4594.68)	5211 (4441–6001)	4396.1 (3773.14–5053.19)	0.3 (0.27–0.33)
Guam	3526 (2985–4083)	4173.95 (3561.64–4824.89)	9901 (8429–11455)	4696.5 (4007.64–5400.01)	0.39 (0.37–0.42)
Hashemite Kingdom of Jordan	49993 (43025–57617)	3479.83 (2986.01–4001.22)	332662 (283508–385073)	3956.28 (3378.63–4517.59)	0.44 (0.42–0.46)
Hellenic Republic	585694 (506602–669450)	3905.01 (3403.26–4454.55)	880644 (757764–1003033)	4167.01 (3580.41–4744.11)	0.19 (0.16–0.22)
Hungary	453680 (389284–518086)	3117.21 (2672.31–3574.57)	600646 (515082–696103)	3290.05 (2822.44–3807.84)	0.17 (0.16–0.18)
Independent State of Papua New Guinea	71082 (60102–83051)	3593.31 (3080.04–4153.91)	223924 (188761–259235)	3845.92 (3289.1–4416.57)	0.18 (0.15–0.2)
Independent State of Samoa	3688 (3098–4233)	4174.39 (3554.97–4780.1)	6827 (5763–7893)	4479 (3815.11–5165.2)	0.17 (0.13–0.22)
Ireland	156088 (134007–179705)	3909.91 (3367.27–4517.75)	317602 (275005–361519)	4171.56 (3618.17–4738.47)	0.21 (0.19–0.22)
Islamic Republic of Afghanistan	218392 (185106–252498)	3054.97 (2604.28–3512.71)	367487 (311043–426796)	3399.78 (2909.72–3904.96)	0.44 (0.4–0.48)
Islamic Republic of Iran	880006 (743025–1011517)	3227.86 (2770.04–3703.38)	2940263 (2520062–3368168)	3595.94 (3094.19–4140.59)	0.34 (0.33–0.35)
Islamic Republic of Mauritania	37455 (31975–42960)	3681.67 (3148.52–4228.43)	91359 (78215–105768)	4025.79 (3469.67–4648.61)	0.26 (0.24–0.27)
Islamic Republic of Pakistan	1640110 (1366597–1932085)	2821.45 (2349.77–3324.24)	4250535 (3535463–4966744)	3213.7 (2693.11–3748.38)	0.5 (0.45–0.54)
Jamaica	70156 (60503–79975)	4074.34 (3520.67–4626.21)	137720 (118808–158584)	4454.13 (3842.91–5127.03)	0.32 (0.29–0.34)
Japan	9097297 (7864578–10385046)	5281.71 (4574.98–6014.74)	16173245 (14066813–18316284)	5331.09 (4598.71–6051.31)	0.04 (0.02–0.06)
Kingdom of Bahrain	7290 (6128–8460)	3597.7 (3077.58–4169.88)	47506 (39539–54790)	3982.96 (3410.03–4546.62)	0.33 (0.32–0.34)
Kingdom of Belgium	574390 (494091–661544)	3872.12 (3351.77–4458.21)	873012 (765528–1006978)	4115.76 (3575.6–4732.95)	0.18 (0.16–0.19)
Kingdom of Bhutan	9088 (7710–10397)	3478.23 (2982.99–3989.31)	24402 (20903–27857)	3880.5 (3327.27–4423.54)	0.38 (0.37–0.4)
Kingdom of Cambodia	138530 (117410–160560)	2900.88 (2486.87–3361.62)	415435 (352913–480035)	3138.79 (2687.24–3602.34)	0.28 (0.27–0.29)
Kingdom of Denmark	286183 (248975–326949)	3723.45 (3231.38–4262.53)	433950 (376400–501332)	3987.37 (3449.18–4546.17)	0.2 (0.17–0.24)
Kingdom of Eswatini	11263 (9538–13118)	3723.31 (3158.77–4292.29)	24040 (20368–27558)	4033.25 (3425.81–4640.02)	0.2 (0.15–0.25)
Kingdom of Lesotho	29321 (25144–33836)	3399.01 (2906.22–3921.06)	41892 (35678–48001)	3750.94 (3233.81–4282.48)	0.32 (0.31–0.33)
Kingdom of Morocco	474891 (404637–543312)	3274.9 (2802.47–3732.28)	1335410 (1140249–1551251)	3713.8 (3186.98–4316.77)	0.41 (0.41–0.42)
Kingdom of Norway	238862 (206682–273108)	3775.32 (3274.65–4328.48)	373658 (324182–430815)	4010.67 (3478.87–4616.94)	0.2 (0.19–0.21)
Kingdom of Saudi Arabia	215917 (182941–250091)	3414.2 (2894.75–3944.19)	987955 (828285–1149038)	4023.43 (3436.35–4620.42)	0.52 (0.5–0.55)
Kingdom of Spain	2103321 (1811409–2433974)	3940.09 (3386.92–4551.32)	3736142 (3252518–4271864)	4181.2 (3624.45–4811.83)	0.17 (0.15–0.19)
Kingdom of Sweden	429758 (367199–506534)	3053.54 (2588.45–3582.26)	638763 (534402–753804)	3312.3 (2766.78–3874.95)	0.2 (0.1–0.3)
Kingdom of Thailand	1209104 (1018955–1397805)	3150.75 (2693.63–3612.62)	4101374 (3501606–4731345)	3720.96 (3189.55–4297.32)	0.59 (0.58–0.61)
Kingdom of the Netherlands	792773 (690006–903653)	4084.32 (3540.59–4662.41)	1408067 (1216956–1604086)	4311.29 (3722.66–4889.26)	0.17 (0.16–0.18)
Kingdom of Tonga	2384 (2019–2773)	4107.59 (3498.54–4753.39)	3762 (3209–4318)	4557.91 (3901.75–5221.75)	0.26 (0.18–0.33)
Kyrgyz Republic	73666 (63111–84837)	2478.85 (2124.46–2839.34)	131156 (111755–150331)	2596.13 (2232.44–2957.17)	0.14 (0.13–0.16)
Lao People's Democratic Republic	62479 (53298–72328)	2883.96 (2473.46–3315.61)	160168 (135102–185302)	3162.03 (2704.98–3639.18)	0.34 (0.33–0.36)
Lebanese Republic	75840 (64454–88592)	3363.65 (2879.78–3912.16)	224446 (193463–257843)	3837.62 (3289.82–4415.64)	0.42 (0.36–0.47)
Malaysia	305669 (259783–353648)	3080.82 (2631.03–3564.78)	1029003 (879590–1191162)	3461.55 (2979.9–4005.92)	0.42 (0.41–0.43)
Mongolia	25690 (21820–29436)	2439.88 (2087.52–2792.23)	64605 (54980–74465)	2600.41 (2239.64–2958.12)	0.09 (0–0.17)
Montenegro	19530 (16701–22530)	3077.25 (2641.01–3540.13)	31260 (26512–35974)	3218.33 (2735.58–3727.31)	0.17 (0.16–0.19)
New Zealand	162168 (140535–186972)	4217.34 (3656.32–4871.82)	372501 (320467–427080)	4650.96 (3996.64–5313.53)	0.32 (0.3–0.34)
North Macedonia	56816 (48316–64965)	2959.68 (2530.05–3406.64)	106211 (90264–122095)	3154.97 (2699.56–3630.34)	0.23 (0.22–0.23)
Northern Mariana Islands	895 (754–1042)	4143.3 (3566.19–4744.05)	2728 (2294–3164)	4584.79 (3944.31–5259.22)	0.27 (0.21–0.33)
Palestine	30128 (25618–34815)	3423.01 (2919.05–3960.81)	102885 (88018–118574)	3685.71 (3182.02–4239.86)	0.21 (0.19–0.23)
People's Democratic Republic of Algeria	423263 (359168–491821)	3327.58 (2843.92–3847.89)	1447429 (1231558–1671316)	3811.37 (3254.92–4380.45)	0.46 (0.45–0.47)
People's Republic of Bangladesh	1608381 (1367349–1839584)	3272.66 (2812.54–3741.38)	5140114 (4372193–5862993)	3582.95 (3067.62–4069.09)	0.33 (0.31–0.35)
People's Republic of China	41044009 (34636657–47406915)	4667.29 (3996.06–5359.85)	109575472 (92723351–126639049)	5016.52 (4265.22–5758.38)	0.5 (0.39–0.61)
Plurinational State of Bolivia	130682 (111962–149840)	3959.6 (3414.71–4534.59)	423951 (360458–487416)	4473.4 (3834.08–5124.94)	0.4 (0.39–0.41)
Portuguese Republic	532111 (457985–605045)	3857.32 (3357.03–4379.17)	926560 (803853–1063052)	4178.17 (3628.09–4802.03)	0.22 (0.19–0.25)
Principality of Andorra	2245 (1936–2594)	3844.45 (3315.52–4443.59)	6349 (5503–7248)	4155.42 (3604.99–4740.56)	0.26 (0.24–0.28)
Principality of Monaco	2600 (2256–3030)	4093.75 (3532.49–4757.69)	3741 (3214–4304)	4300.56 (3702.01–4898.03)	0.15 (0.14–0.16)
Puerto Rico	163281 (140856–188940)	4522.24 (3903.55–5228.2)	316244 (272839–362469)	4957.79 (4258.87–5636.66)	0.33 (0.31–0.36)
Republic of Albania	60340 (51813–68996)	2836.81 (2441.91–3228.51)	132531 (113298–153196)	3086.21 (2645.23–3555.66)	0.31 (0.3–0.31)
Republic of Angola	131942 (111407–153171)	3185.91 (2727.69–3659.36)	453310 (382775–524462)	3498.42 (2993.53–3990.41)	0.31 (0.3–0.33)
Republic of Armenia	68518 (58314–78562)	2463.37 (2103.7–2820.74)	115968 (100557–133873)	2698.2 (2345.63–3106.37)	0.33 (0.32–0.34)
Republic of Austria	445917 (388835–511161)	3930.56 (3422.97–4498.89)	695927 (603795–795871)	4158.81 (3598.54–4757.78)	0.17 (0.16–0.17)
Republic of Azerbaijan	127027 (108248–145409)	2509.29 (2143.68–2856.54)	299059 (252802–345606)	2743.78 (2352.31–3175.04)	0.33 (0.3–0.36)
Republic of Belarus	413994 (355113–474109)	3185.62 (2738.3–3657.52)	541760 (462469–627494)	3433.67 (2941.39–3964.55)	0.27 (0.25–0.29)
Republic of Benin	70618 (59924–81702)	3499.85 (2974.17–4036.08)	216260 (182858–250330)	3906.34 (3333.79–4504.34)	0.36 (0.33–0.39)
Republic of Botswana	19845 (17007–22822)	3423.94 (2945.33–3923.76)	62286 (53272–71430)	3911.53 (3359.74–4485.45)	0.43 (0.41–0.45)
Republic of Bulgaria	383783 (325745–440869)	3071.08 (2628.8–3528.5)	432007 (369658–503657)	3211.35 (2748.52–3723.95)	0.13 (0.12–0.15)
Republic of Burundi	75209 (64211–86182)	3190.83 (2734.12–3659.71)	170394 (144398–197028)	3240.87 (2766.07–3732.76)	0.05 (0.04–0.06)
Republic of Cabo Verde	7804 (6681–9042)	3563.58 (3056.62–4129.19)	18400 (15508–21328)	3965.99 (3358.58–4574.49)	0.35 (0.34–0.36)
Republic of Cameroon	177268 (150249–204928)	3781.2 (3215.34–4364.02)	555674 (474232–637988)	4066.17 (3487.26–4668.74)	0.21 (0.2–0.22)
Republic of Chad	94371 (80813–108902)	3317.42 (2831.87–3828.17)	216396 (184901–251753)	3484.52 (2964.82–4041.53)	0.14 (0.14–0.15)
Republic of Chile	432139 (372442–499516)	4232.2 (3674.19–4889.58)	1197992 (1040854–1370000)	4714.37 (4096.4–5375.03)	0.33 (0.29–0.36)
Republic of Colombia	727430 (624241–835622)	3999.73 (3452.66–4596.7)	2456831 (2128652–2827233)	4431.24 (3846.08–5099.92)	0.33 (0.32–0.34)
Republic of Costa Rica	72936 (62727–83855)	4125.05 (3551.51–4732.83)	251057 (215967–290299)	4542.72 (3916.42–5249.56)	0.31 (0.3–0.32)
Republic of Croatia	187930 (161636–216927)	3012.74 (2599.33–3468.96)	265456 (228061–305841)	3219.92 (2754.19–3694.81)	0.26 (0.24–0.27)
Republic of Cuba	415811 (358678–476870)	4064.82 (3496.46–4659.26)	860421 (737457–986448)	4484.75 (3839.78–5121.43)	0.35 (0.34–0.37)
Republic of Cyprus	30822 (26705–35058)	3718.02 (3228.54–4234.67)	81855 (70941–94417)	4024.52 (3478.54–4627.78)	0.27 (0.25–0.29)
Republic of C么te d'Ivoire	153955 (131252–178607)	3537.6 (3033.48–4082.15)	471219 (400510–544117)	3809.3 (3269.11–4418.26)	0.22 (0.2–0.23)
Republic of Djibouti	4798 (4049–5560)	3190.35 (2728.24–3637.24)	25514 (21550–29498)	3528.87 (3023.13–4071.53)	0.37 (0.35–0.38)
Republic of Ecuador	231156 (200312–263664)	4258.87 (3688.22–4856.53)	788971 (677373–901237)	4753.34 (4079.78–5423.74)	0.41 (0.39–0.44)
Republic of El Salvador	121168 (103491–138779)	4049.15 (3482.57–4631.44)	275821 (240206–314658)	4528.27 (3928.7–5166.45)	0.37 (0.33–0.4)
Republic of Equatorial Guinea	6511 (5510–7588)	3228.6 (2764.52–3734.87)	21470 (18195–24755)	3849.83 (3292.97–4445.22)	0.68 (0.64–0.72)
Republic of Estonia	66220 (56940–77124)	3234.22 (2784.18–3754.32)	85919 (74036–98759)	3515.13 (2992.55–4030.27)	0.32 (0.3–0.33)
Republic of Fiji	15877 (13463–18425)	3988.89 (3409.05–4622.44)	37918 (32061–44138)	4554.43 (3901.75–5281.44)	0.38 (0.34–0.42)
Republic of Finland	276093 (238397–315027)	3956.76 (3413.89–4490.47)	469620 (405078–545172)	4177.96 (3620.74–4828.33)	0.18 (0.16–0.19)
Republic of Ghana	228914 (193866–266163)	3480.1 (2980.07–4018.7)	717795 (608288–827958)	3971.54 (3381.31–4554.93)	0.42 (0.41–0.43)
Republic of Guatemala	138417 (117395–159527)	3819.63 (3270.99–4379.74)	477914 (411558–545062)	4239.1 (3649.81–4845.24)	0.33 (0.32–0.35)
Republic of Guinea	115448 (98317–133364)	3428.51 (2922.32–3944.21)	215046 (183953–247996)	3648.64 (3142.27–4175.64)	0.18 (0.17–0.19)
Republic of Guinea-Bissau	14078 (11919–16275)	3439.33 (2918.81–3995.45)	28800 (24420–33128)	3653.92 (3115.86–4212.35)	0.17 (0.16–0.18)
Republic of Guyana	15584 (13398–17864)	3998.53 (3428.16–4566.43)	29569 (25431–33923)	4390.4 (3773.35–5000.62)	0.3 (0.28–0.32)
Republic of Haiti	123331 (105167–140803)	3701.53 (3171.88–4239.19)	301617 (256108–347558)	3913.76 (3348.87–4499.51)	0.2 (0.2–0.21)
Republic of Honduras	81897 (69522–93542)	3897.21 (3308.68–4425.53)	285498 (245698–327845)	4310.1 (3699.5–4944.41)	0.33 (0.3–0.35)
Republic of Iceland	10900 (9505–12597)	3956.39 (3416.3–4548.45)	23114 (20024–26528)	4229.86 (3666.61–4826.45)	0.22 (0.21–0.23)
Republic of India	17412254 (14805069–19971633)	3538.82 (3043.58–4028.26)	48463957 (41607887–55215872)	3911.42 (3368.5–4448.98)	0.35 (0.34–0.36)
Republic of Indonesia	3019331 (2558413–3482089)	2872.4 (2464.84–3286.94)	8455534 (7153574–9778458)	3186.42 (2737.63–3663.66)	0.38 (0.36–0.39)
Republic of Iraq	288596 (246516–331137)	3571.54 (3054.85–4073.3)	992738 (846877–1149354)	3803.13 (3226.04–4377.09)	0.22 (0.2–0.24)
Republic of Italy	3361885 (2925969–3864633)	3875.64 (3373.53–4435.14)	5275983 (4606861–6017508)	4061.26 (3515.73–4618.4)	0.13 (0.12–0.15)
Republic of Kazakhstan	335773 (286944–388021)	2633.54 (2252.16–3050.59)	524726 (445801–602127)	2829.07 (2426.61–3239.31)	0.22 (0.21–0.24)
Republic of Kenya	277936 (236953–318257)	3279.31 (2804.39–3775.64)	876838 (744710–1006917)	3569.94 (3058.54–4110.65)	0.28 (0.26–0.31)
Republic of Kiribati	1544 (1309–1786)	3980.87 (3416.91–4587.81)	3480 (2939–4001)	4389.98 (3768.05–5002.06)	0.27 (0.21–0.33)
Republic of Korea	1903781 (1628477–2205126)	6160.49 (5327.87–7052.88)	5835363 (5072731–6672902)	6201.62 (5389.51–7093.43)	0.2 (0.07–0.33)
Republic of Latvia	116754 (100005–136052)	3262.48 (2798.23–3783.17)	127599 (109617–146829)	3502.9 (3027.96–4013.9)	0.25 (0.24–0.26)
Republic of Liberia	41505 (35542–47709)	3563.1 (3048.1–4092.98)	92742 (78346–107508)	3901.49 (3335.12–4544.29)	0.37 (0.33–0.41)
Republic of Lithuania	145934 (125952–166505)	3229.52 (2796.17–3674.17)	184271 (158593–213836)	3462.18 (2963.32–4000.86)	0.25 (0.23–0.27)
Republic of Madagascar	164987 (141132–191791)	3165.22 (2723.11–3662.69)	411396 (344172–475845)	3307 (2818.24–3800.32)	0.17 (0.16–0.18)
Republic of Malawi	130113 (110363–150091)	3265.16 (2787.72–3761.93)	276585 (234559–317633)	3517.8 (2993.71–4046.61)	0.27 (0.26–0.29)
Republic of Maldives	2784 (2337–3267)	2792.59 (2383.92–3243.97)	12808 (10811–14913)	3243.86 (2775.43–3750.72)	0.58 (0.55–0.62)
Republic of Mali	139968 (118141–162045)	3359.76 (2871.21–3856.83)	340281 (288934–390685)	3609.45 (3102.06–4120.09)	0.24 (0.23–0.24)
Republic of Malta	16495 (14314–18708)	3852.25 (3351.51–4359.87)	36704 (32099–42341)	4140.39 (3614.23–4756.12)	0.21 (0.17–0.25)
Republic of Mauritius	23628 (20026–27150)	3132.58 (2678.79–3587.9)	65395 (56034–75597)	3494.9 (3012.26–4024.03)	0.38 (0.37–0.4)
Republic of Moldova	138357 (117722–159612)	3108.29 (2661.41–3573.16)	201154 (172093–233714)	3426.84 (2939.9–3949.97)	0.37 (0.34–0.41)
Republic of Mozambique	201668 (171171–231774)	3203.3 (2740.03–3688.16)	411261 (347402–472241)	3444.43 (2930.11–3973.76)	0.26 (0.25–0.27)
Republic of Namibia	22687 (19337–26266)	3394.59 (2916.9–3910.94)	53863 (46328–62343)	3679.63 (3168.58–4214.31)	0.24 (0.23–0.26)
Republic of Nauru	199 (168–229)	3940.32 (3377.82–4529.85)	286 (242–331)	4546.58 (3873.76–5250.08)	0.41 (0.38–0.43)
Republic of Nicaragua	62666 (53894–71621)	3968.23 (3413.06–4543.17)	222878 (190363–254152)	4396.99 (3772.45–5016.02)	0.33 (0.31–0.36)
Republic of Niue	90 (77–103)	4188.29 (3585.03–4838.67)	104 (89–119)	4720.19 (4062.7–5402.23)	0.37 (0.33–0.41)
Republic of Palau	423 (361–487)	4188.61 (3590.68–4797.4)	1190 (997–1372)	4641.84 (3959.09–5315.6)	0.28 (0.24–0.33)
Republic of Panama	58083 (50058–66127)	3840.54 (3316.35–4381.42)	193692 (166733–220672)	4376.1 (3772.28–4986)	0.4 (0.39–0.41)
Republic of Paraguay	90987 (77404–103800)	4038.13 (3448.24–4602.39)	261925 (225199–300001)	4386.47 (3780–5030.18)	0.27 (0.27–0.28)
Republic of Peru	491622 (422625–565510)	4016.65 (3462.25–4641.56)	1545296 (1319661–1780663)	4540.07 (3880.48–5217.4)	0.42 (0.41–0.43)
Republic of Poland	1301420 (1114310–1502244)	2983.22 (2561.68–3434.04)	2204604 (1895877–2552373)	3243.33 (2778.91–3732.74)	0.28 (0.27–0.28)
Republic of Rwanda	94495 (80665–108099)	3255.48 (2794.73–3718.5)	235781 (200526–270812)	3480.18 (2976.96–3987.41)	0.24 (0.23–0.26)
Republic of San Marino	1344 (1160–1557)	3983.58 (3423.38–4573.39)	2826 (2452–3227)	4224.91 (3624.56–4835.89)	0.18 (0.16–0.2)
Republic of Senegal	117902 (99921–136625)	3544.71 (3032.63–4108.31)	310501 (262844–358435)	3792.46 (3213.76–4355.86)	0.18 (0.17–0.19)
Republic of Serbia	350644 (297685–401102)	3009.87 (2581.77–3436.16)	503533 (432137–583590)	3224.1 (2767.64–3727.05)	0.26 (0.25–0.27)
Republic of Seychelles	1791 (1532–2059)	3244.9 (2768.71–3727.15)	4398 (3707–5069)	3509.68 (2993.61–4022.97)	0.25 (0.24–0.27)
Republic of Sierra Leone	70651 (60694–80839)	3413.85 (2927.59–3912.54)	146761 (125017–168931)	3661.3 (3122.7–4203.61)	0.21 (0.2–0.22)
Republic of Singapore	128188 (109955–146159)	5559.36 (4791.6–6298.06)	509663 (438044–580344)	5810.72 (5004.32–6619.23)	0.13 (0.11–0.15)
Republic of Slovenia	74593 (64009–85641)	3033.02 (2611.61–3475.54)	131975 (114507–152187)	3242.46 (2793.14–3734.55)	0.24 (0.22–0.25)
Republic of South Africa	774248 (658540–891776)	3685.35 (3152.19–4255.43)	1917337 (1640743–2211862)	3971.89 (3413.32–4567.72)	0.26 (0.26–0.27)
Republic of South Sudan	83561 (71077–96648)	3217.86 (2748.54–3710.36)	147378 (124240–170165)	3433.35 (2917.58–3949.03)	0.23 (0.22–0.24)
Republic of Sudan	292018 (250113–336125)	3075.36 (2630.42–3533.51)	758628 (647046–878301)	3591.14 (3065.99–4121.58)	0.53 (0.5–0.55)
Republic of Suriname	10740 (9261–12315)	4089.85 (3536.02–4681.38)	29546 (25322–33817)	4523.48 (3885.29–5157.56)	0.35 (0.34–0.36)
Republic of Tajikistan	64397 (55427–73905)	2349.15 (2031.07–2697.74)	151585 (129545–173088)	2425.49 (2101.17–2773.29)	0.1 (0.08–0.12)
Republic of the Congo	37480 (31769–43178)	3408.12 (2908.81–3910.41)	109711 (92786–126668)	3655.4 (3143.98–4173.09)	0.23 (0.23–0.23)
Republic of the Gambia	12712 (10742–14701)	3468.34 (2937.94–4009.01)	39169 (33424–45070)	3806.01 (3241.59–4367.38)	0.29 (0.28–0.3)
Republic of the Marshall Islands	627 (535–724)	3729.59 (3200.37–4300.07)	1645 (1399–1887)	4146.65 (3550.51–4724.54)	0.29 (0.27–0.32)
Republic of the Niger	98866 (84284–114015)	3346.16 (2880–3864.36)	311987 (265585–363171)	3542.89 (3041.4–4119.44)	0.18 (0.17–0.19)
Republic of the Philippines	807456 (684598–931822)	2558.32 (2191.01–2945)	2560788 (2167721–2960350)	2924.64 (2506.01–3372.69)	0.45 (0.41–0.48)
Republic of the Union of Myanmar	691310 (587687–802417)	2874.45 (2466.43–3303.11)	1665523 (1409884–1932984)	3210.72 (2736.45–3689.26)	0.41 (0.39–0.43)
Republic of Trinidad and Tobago	35869 (30887–41681)	4225.65 (3631.05–4902.09)	89459 (76903–103166)	4589.04 (3951.12–5280.51)	0.32 (0.3–0.34)
Republic of Tunisia	173096 (146930–199767)	3350.37 (2864.68–3860.78)	516592 (444113–594564)	3757.41 (3239.13–4299.18)	0.38 (0.37–0.39)
Republic of Turkey	1302667 (1111261–1500768)	3616.91 (3108.74–4185.48)	3933251 (3382509–4552904)	4066.07 (3515.09–4690.46)	0.39 (0.37–0.41)
Republic of Uganda	212429 (182240–245211)	3199.56 (2755.77–3682)	546939 (467171–631007)	3459.97 (2962.97–3971.14)	0.28 (0.27–0.29)
Republic of Uzbekistan	283780 (242051–326935)	2469.29 (2114.65–2843.79)	741559 (629539–859047)	2673.49 (2306.68–3116.56)	0.27 (0.25–0.28)
Republic of Vanuatu	2533 (2142–2926)	3741.42 (3186.53–4293.62)	7991 (6728–9257)	4171.74 (3566.38–4792.92)	0.34 (0.32–0.35)
Republic of Yemen	153476 (130282–177993)	3013.69 (2573.39–3485.51)	501476 (428203–577144)	3301.67 (2826.77–3793.51)	0.33 (0.31–0.34)
Republic of Zambia	98475 (83748–113144)	3303.37 (2809.17–3804.77)	267275 (227279–309997)	3534.91 (3047.13–4080.59)	0.23 (0.21–0.25)
Republic of Zimbabwe	144933 (124368–166808)	3432.78 (2951.07–3931.44)	264469 (224604–304783)	3569.41 (3056.52–4110.59)	0.08 (0.06–0.1)
Romania	837338 (712366–966691)	2960.41 (2536.16–3398.26)	1130574 (971118–1302491)	3219.66 (2772.22–3724.14)	0.3 (0.29–0.31)
Russian Federation	5693406 (4865290–6531356)	3138 (2695.01–3602.6)	8092104 (6934167–9299270)	3428.99 (2949.71–3939.79)	0.32 (0.31–0.33)
Saint Kitts and Nevis	1497 (1295–1724)	4228.46 (3614.57–4832.65)	3447 (2920–3977)	4618.37 (3958.03–5318.13)	0.28 (0.27–0.3)
Saint Lucia	3554 (3047–4087)	4095.87 (3500.68–4726.59)	11015 (9383–12614)	4491.77 (3830.98–5135.22)	0.28 (0.26–0.31)
Saint Vincent and the Grenadines	2808 (2390–3226)	4013.87 (3421.05–4615.59)	6430 (5479–7357)	4414.21 (3776.78–5031.68)	0.34 (0.33–0.36)
Slovak Republic	180083 (154628–208829)	3039.12 (2611.79–3511.42)	300055 (255138–349169)	3245.2 (2760.55–3758.97)	0.19 (0.18–0.21)
Socialist Republic of Viet Nam	1154490 (988840–1322473)	2872.27 (2466.33–3287.59)	3280643 (2784356–3782298)	3098.78 (2649.06–3558.56)	0.29 (0.27–0.31)
Solomon Islands	5432 (4579–6317)	3645.58 (3116.34–4199.5)	15814 (13333–18402)	4098.69 (3516.91–4709.91)	0.33 (0.28–0.38)
State of Eritrea	38289 (32156–44386)	3122.88 (2677.63–3600.21)	100071 (85520–115107)	3297.18 (2851.83–3793.18)	0.18 (0.16–0.19)
State of Israel	183686 (159563–211016)	3862.31 (3349.75–4427.67)	487869 (423741–557538)	4156.01 (3595.59–4754.83)	0.21 (0.18–0.24)
State of Kuwait	25658 (21586–29713)	3629.92 (3098.88–4194.06)	152120 (127811–177115)	4103.51 (3499.68–4741.94)	0.46 (0.44–0.48)
State of Libya	69287 (59210–80370)	3540.68 (3024.61–4071.73)	226868 (191953–260963)	3883.93 (3318.01–4443.16)	0.31 (0.3–0.33)
State of Qatar	5840 (4819–6841)	3671.08 (3118.6–4257.86)	64293 (53657–74410)	4116.78 (3529.89–4751.62)	0.34 (0.31–0.36)
Sultanate of Oman	23778 (20222–27661)	3201.73 (2748.75–3676.21)	97626 (83255–112954)	3896.83 (3357.37–4450.96)	0.68 (0.66–0.7)
Swiss Confederation	384283 (330922–438909)	3870.53 (3325.93–4414.23)	666652 (576401–771112)	4026.29 (3474.53–4665.92)	0.13 (0.12–0.14)
Syrian Arab Republic	183305 (156684–211533)	3378.1 (2912.62–3887.75)	539745 (456008–625242)	3728.93 (3179.23–4297.81)	0.3 (0.28–0.33)
Taiwan (Province of China)	759041 (644296–879006)	4575.66 (3911–5280.57)	2169014 (1859175–2462678)	5263.61 (4510.48–5989.03)	0.53 (0.5–0.56)
Togolese Republic	45085 (38405–51860)	3450.78 (2961.82–3982.21)	157798 (133712–181501)	3740.77 (3218.5–4314.53)	0.24 (0.23–0.26)
Tokelau	52 (44–60)	3946.29 (3368.13–4530.32)	66 (57–76)	4500.31 (3852.28–5158.46)	0.4 (0.37–0.44)
Turkmenistan	48118 (40817–55574)	2511.52 (2134.11–2896.62)	114907 (97831–131377)	2732.86 (2349.02–3125.16)	0.29 (0.28–0.31)
Tuvalu	275 (232–315)	3948.23 (3378.26–4519.12)	473 (404–544)	4380.84 (3762.6–5011.29)	0.29 (0.25–0.33)
Ukraine	2279101 (1944838–2630430)	3180.29 (2718.32–3660.01)	2529968 (2160023–2923027)	3331.62 (2843.68–3849.75)	0.18 (0.16–0.19)
Union of the Comoros	6772 (5758–7875)	3296.43 (2822.19–3789.54)	18135 (15508–20730)	3509.56 (3018.15–4002.97)	0.21 (0.2–0.21)
United Arab Emirates	20717 (17267–24217)	3450.79 (2950.47–3957.2)	302515 (250446–355876)	3885.45 (3316.69–4445.81)	0.41 (0.39–0.43)
United Kingdom of Great Britain and Northern Ireland	3582601 (3116475–4119177)	4174.32 (3627.99–4778.45)	5346842 (4647728–6099658)	4480.33 (3893.78–5098.98)	0.27 (0.2–0.34)
United Mexican States	1846895 (1582223–2109593)	4210.92 (3626.19–4839.1)	5952758 (5103285–6835348)	4561.81 (3932.39–5223.76)	0.26 (0.25–0.27)
United Republic of Tanzania	371041 (315003–428986)	3309.6 (2822.88–3829.44)	974971 (825614–1125651)	3568.24 (3045.74–4092.26)	0.26 (0.25–0.27)
United States of America	14378724 (12464702–16442866)	4724.28 (4082.75–5379.27)	27188653 (23424909–31332329)	4949.15 (4275.55–5677.95)	−0.12 (−0.32–0.08)
United States Virgin Islands	4033 (3444–4683)	4485.92 (3846.08–5172.98)	8448 (7205–9728)	4854.26 (4165.75–5513.4)	0.26 (0.24–0.29)

**Table 3:** The number of DALYs cases and the age-standardized DALYs rate attributable to osteoarthritis knee in 1990 and 2021, and its trends from 1990 to 2021 globally

	Number of DALYs cases (95% UI) in 1990	The age-standardized DALYs rate/100000 (95% UI) in 1990	Number of DALYs cases (95% UI) in 2021	The age-standardized DALYs rate/100000 (95% UI) in 2021	EAPC (95% CI)
Global	5145339 (2507481–9953258)	127.14 (62.17–246.99)	12019070 (5858108–23267858)	137.59 (67.08–266.87)	0.33 (0.3–0.36)
Sex					
Female	3155786 (1543268–6114836)	147.59 (72.23–286.21)	7344351 (3585648–14249004)	160.61 (78.3–311.53)	0.36 (0.33–0.4)
Male	1989553 (966778–3838422)	103.89 (50.66–202.11)	4674719 (2271426–9018855)	112.3 (54.71–217.62)	0.31 (0.28–0.33)
Age					
< 5 years	0 (0–0)	0 (0–0)	0 (0–0)	0 (0–0)	0 (0–0)
5–9 years	0 (0–0)	0 (0–0)	0 (0–0)	0 (0–0)	0 (0–0)
10–14 years	0 (0–0)	0 (0–0)	0 (0–0)	0 (0–0)	0 (0–0)
15–19 years	0 (0–0)	0 (0–0)	0 (0–0)	0 (0–0)	0 (0–0)
20–24 years	0 (0–0)	0 (0–0)	0 (0–0)	0 (0–0)	0 (0–0)
25–29 years	0 (0–0)	0 (0–0)	0 (0–0)	0 (0–0)	0 (0–0)
30–34 years	13418 (5981–28967)	3.48 (1.55–7.52)	22762 (10285–48341)	3.77 (1.7–8)	0.39 (0.29–0.5)
35–39 years	115707 (53473–244354)	32.85 (15.18–69.37)	196770 (92137–414118)	35.08 (16.43–73.84)	0.43 (0.29–0.57)
40–44 years	273110 (130079–572483)	95.33 (45.41–199.83)	508638 (242654–1060242)	101.68 (48.51–211.94)	0.47 (0.32–0.62)
45–49 years	449553 (213071–879903)	193.61 (91.76–378.95)	999525 (471177–1959034)	211.09 (99.51–413.73)	0.49 (0.39–0.59)
50–54 years	657495 (304248–1323761)	309.31 (143.13–622.74)	1539134 (709506–3079501)	345.93 (159.47–692.14)	0.47 (0.43–0.51)
55–59 years	770407 (361595–1477016)	415.99 (195.25–797.52)	1818906 (854416–3485634)	459.64 (215.91–880.82)	0.38 (0.34–0.43)
60–64 years	805154 (383780–1551197)	501.31 (238.95–965.82)	1732661 (825739–3327842)	541.38 (258–1039.79)	0.35 (0.31–0.39)
65–69 years	717874 (343737–1407970)	580.76 (278.08–1139.05)	1714937 (823039–3354471)	621.71 (298.37–1216.08)	0.28 (0.26–0.31)
70–74 years	544835 (263996–1083001)	643.55 (311.83–1279.22)	1407034 (682030–2808605)	683.56 (331.34–1364.47)	0.19 (0.17–0.22)
75–79 years	420765 (203795–867850)	683.55 (331.07–1409.87)	951390 (459300–1961106)	721.38 (348.26–1486.99)	0.14 (0.11–0.17)
80–84 years	245168 (119284–498036)	693.04 (337.19–1407.84)	644625 (312180–1305882)	736.02 (356.44–1491.02)	0.16 (0.14–0.18)
85–89 years	100320 (49271–201462)	663.88 (326.06–1333.21)	329199 (162446–654622)	720 (355.29–1431.75)	0.24 (0.22–0.26)
90–94 years	26038 (12907–52530)	607.64 (301.19–1225.84)	119876 (60010–242154)	670.1 (335.45–1353.62)	0.34 (0.32–0.35)
95 + years	5496 (2740–11036)	539.85 (269.16–1083.98)	33612 (16822–67894)	616.7 (308.65–1245.7)	0.45 (0.43–0.46)
SDI region					
High-middle SDI	1224964 (596148–2368347)	121.29 (59.1–235.64)	2737095 (1331220–5281509)	138.4 (67.13–267.45)	0.55 (0.49–0.61)
High SDI	1515496 (742820–2979335)	140.18 (68.63–274.58)	2864761 (1411755–5666715)	149.14 (73.09–292.89)	0.15 (0.12–0.19)
Low-middle SDI	701057 (340832–1342497)	108.81 (53.29–210.14)	1815224 (884403–3477190)	120.44 (59.02–231.75)	0.36 (0.35–0.37)
Low SDI	250983 (122046–481344)	105.33 (51.63–203.7)	614610 (299059–1167797)	112.74 (55.22–216.52)	0.24 (0.23–0.25)
Middle SDI	1448003 (701826–2778682)	132.02 (64.04–255.23)	3978378 (1922896–7662185)	141.17 (68.46–273.3)	0.38 (0.32–0.45)
GBD region					
Advanced Health System	2090089 (1025572–4101402)	129.38 (63.47–253.5)	3702137 (1824100–7319249)	139.94 (68.63–274.23)	0.21 (0.19–0.24)
Africa	318689 (154785–612657)	107.86 (52.75–209.27)	830083 (402895–1587917)	117.53 (57.69–227.68)	0.28 (0.27–0.29)
African Region	250798 (121907–481661)	108.04 (52.93–209.62)	644060 (312758–1226241)	116.88 (57.42–225.85)	0.27 (0.25–0.28)
America	832700 (409849–1630226)	137.48 (67.65–268.45)	1928303 (948414–3774285)	145.74 (71.62–284.95)	0.07 (−0.02–0.16)
Andean Latin America	27640 (13612–53615)	131.2 (64.79–255.6)	88874 (43242–172157)	147.55 (72.02–286.28)	0.42 (0.4–0.43)
Asia	2810178 (1358426–5394056)	131.63 (63.84–254.54)	7408395 (3581943–14270418)	141.58 (68.61–273.78)	0.39 (0.33–0.45)
Australasia	31751 (15676–63068)	137.26 (67.72–271.84)	76128 (37728–153473)	154.18 (75.68–309.09)	0.36 (0.33–0.38)
Basic Health System	2069659 (999739–3968940)	132.83 (64.21–256.13)	5652298 (2733372–10872184)	144.07 (69.67–278.48)	0.45 (0.37–0.52)
Caribbean	34448 (16791–66909)	131.6 (64.27–255.98)	77063 (38028–151478)	142.84 (70.48–281.03)	0.3 (0.29–0.32)
Central Africa	31842 (15521–60994)	107.2 (52.24–207.64)	82143 (39688–158077)	112.64 (54.59–219.65)	0.14 (0.12–0.15)
Central Asia	38487 (18666–73649)	81.49 (39.7–157.58)	74350 (36555–143465)	86.71 (42.6–169.71)	0.21 (0.2–0.23)
Central Europe	143854 (70890–278718)	95.69 (47.26–186)	215177 (106271–422092)	103.21 (50.75–200.27)	0.27 (0.26–0.27)
Central Latin America	113158 (55122–217637)	131.42 (64.27–254.9)	368356 (179925–711864)	143.61 (70.3–278.58)	0.29 (0.27–0.3)
Central Sub-Saharan Africa	24860 (12133–47848)	104.1 (50.94–202.82)	66124 (32089–127548)	109.23 (53.33–213.55)	0.14 (0.12–0.16)
Commonwealth High Income	178828 (88340–355047)	123.03 (60.66–242.41)	330316 (163533–655862)	132.44 (65.3–260.75)	0.23 (0.2–0.26)
Commonwealth Low Income	95386 (46620–184814)	104.41 (51.28–203.29)	277019 (135620–531875)	113.29 (55.56–218.62)	0.3 (0.29–0.31)
Commonwealth Middle Income	730365 (355032–1400840)	109.31 (53.52–211.15)	1997040 (973579–3825779)	121.17 (59.4–232.96)	0.37 (0.36–0.38)
East Asia	1390648 (668816–2682918)	151.13 (72.88–291.29)	3677623 (1773767–7079261)	162.47 (78.29–314.12)	0.5 (0.38–0.61)
East Asia & Pacific—WB	2027563 (977572–3895686)	144.43 (69.71–279.18)	5192685 (2501864–10028708)	153.78 (74.08–297.88)	0.4 (0.32–0.49)
Eastern Africa	74006 (35979–142345)	101.69 (49.86–198.07)	190904 (93143–363044)	110.63 (54.41–213.45)	0.31 (0.29–0.32)
Eastern Europe	281591 (138411–548173)	100.24 (49.43–195.97)	372649 (183304–722199)	108.31 (53.24–209.35)	0.29 (0.28–0.31)
Eastern Mediterranean Region	190685 (92973–365522)	100.92 (49.32–195.11)	568055 (277028–1093765)	114.13 (55.77–221.25)	0.42 (0.41–0.43)
Eastern Sub-Saharan Africa	80099 (38879–153647)	102.41 (50.13–199.17)	200595 (97952–381074)	110.05 (54.14–212.29)	0.27 (0.25–0.28)
Europe	1173264 (576644–2305248)	114.16 (56.12–223.75)	1832284 (902596–3611281)	122.95 (60.32–239.87)	0.26 (0.24–0.27)
Europe & Central Asia—WB	1196887 (588014–2350406)	113.2 (55.63–221.83)	1881525 (926633–3706045)	121.46 (59.59–237.06)	0.24 (0.23–0.26)
European Region	1205135 (592040–2366907)	113.23 (55.64–221.91)	1901698 (936600–3746240)	121.52 (59.63–237.21)	0.24 (0.23–0.26)
High-income Asia Pacific	360521 (174917–701559)	174.98 (85.11–341.43)	722000 (353789–1446533)	180.61 (88.02–356.06)	0.16 (0.12–0.2)
High-income North America	482468 (237787–957742)	143.91 (70.69–283.92)	911621 (449630–1807879)	148.92 (73.19–294.55)	−0.15 (−0.34–0.05)
Latin America & Caribbean—WB	355671 (174510–685718)	130.09 (64.12–252.45)	1027085 (503808–1990657)	143.55 (70.5–278.86)	0.33 (0.32–0.35)
Limited Health System	915396 (444562–1753898)	108.64 (53.2–210.04)	2504815 (1220511–4789221)	119.89 (58.79–230.53)	0.35 (0.34–0.36)
Middle East & North Africa—WB	139054 (67490–268430)	108.18 (52.9–210.75)	451552 (219436–872764)	120.39 (59.03–234.86)	0.34 (0.33–0.35)
Minimal Health System	65359 (31771–125240)	104.19 (50.86–201.81)	150819 (72869–287875)	110.82 (54.09–213.82)	0.21 (0.2–0.21)
North Africa and Middle East	191120 (92640–367986)	108.26 (52.86–210.57)	596794 (290214–1148221)	121.33 (59.3–235.41)	0.37 (0.37–0.38)
North America	482519 (237811–957845)	143.92 (70.7–283.93)	911733 (449685–1808113)	148.92 (73.2–294.57)	−0.15 (−0.34–0.05)
Northern Africa	70476 (34081–137490)	108.8 (52.9–213.78)	202960 (98564–396697)	121.74 (59.48–240.16)	0.35 (0.34–0.36)
Oceania	3889 (1841–7536)	119.61 (57.24–233.97)	10965 (5248–21272)	128.75 (62.39–250.96)	0.2 (0.17–0.23)
Region of the Americas	832700 (409849–1630226)	137.48 (67.65–268.45)	1928303 (948414–3774285)	145.74 (71.62–284.95)	0.07 (−0.02–0.16)
South-East Asia Region	814447 (395148–1562292)	107.59 (52.64–208.29)	2280261 (1110983–4358582)	119.55 (58.51–229.75)	0.38 (0.37–0.39)
South Asia	669709 (326141–1287959)	108.55 (53.23–210.18)	1872013 (913685–3586458)	120.81 (59.17–232.43)	0.38 (0.37–0.39)
South Asia—WB	687691 (334887–1322033)	108.15 (53.04–209.33)	1912798 (933513–3664794)	120.38 (58.98–231.58)	0.38 (0.37–0.39)
Southeast Asia	252388 (122330–486666)	92.87 (45.45–179.67)	736837 (356755–1411054)	104.35 (50.89–201.33)	0.43 (0.41–0.44)
Southern Africa	50304 (24439–96488)	111 (54.37–215.38)	120438 (58860–230394)	118.78 (58.47–229.87)	0.24 (0.23–0.24)
Southern Latin America	61860 (30519–121117)	132.85 (65.49–260.41)	126220 (61981–249769)	148.13 (72.64–291.9)	0.34 (0.31–0.38)
Southern Sub-Saharan Africa	32212 (15730–62021)	115.9 (56.92–225.01)	75140 (36629–144659)	123.64 (60.67–240.41)	0.23 (0.22–0.23)
Sub-Saharan Africa—WB	249267 (121232–478045)	107.66 (52.74–208.86)	629253 (305468–1196971)	116.35 (57.1–224.54)	0.26 (0.25–0.28)
Tropical Latin America	119829 (58969–229561)	126.74 (62.67–245)	369382 (181038–712660)	141.07 (69.25–272.84)	0.38 (0.37–0.39)
Western Africa	92061 (44827–176554)	111.01 (54.37–214.95)	233638 (113044–443571)	121.35 (59.48–233.18)	0.29 (0.24–0.34)
Western Europe	702719 (344993–1392177)	126.28 (61.88–248.06)	1119462 (552620–2232285)	133.93 (65.73–263.33)	0.19 (0.17–0.21)
Western Pacific Region	1816181 (875788–3487897)	151.24 (72.98–292.21)	4606345 (2221967–8914311)	160.51 (77.32–311.3)	0.41 (0.32–0.5)
Western Sub-Saharan Africa	102089 (49668–195744)	111.44 (54.56–215.6)	261698 (126482–497450)	121.72 (59.6–234.01)	0.28 (0.24–0.33)
World Bank High Income	1719294 (843445–3385757)	136.47 (66.87–267.77)	3142021 (1549719–6236746)	145.82 (71.54–286.73)	0.16 (0.12–0.19)
World Bank Low Income	167688 (81174–319216)	108.3 (52.74–209.01)	402730 (194781–766019)	115.26 (56.42–221.78)	0.22 (0.21–0.23)
World Bank Lower Middle Income	1147227 (557872–2200641)	104.64 (51.28–202.51)	3081747 (1501878–5891933)	115.87 (56.81–223)	0.36 (0.35–0.37)
World Bank Upper Middle Income	2114439 (1021775–4052241)	135.08 (65.3–260.57)	5407216 (2615955–10416624)	149.54 (72.23–289.09)	0.52 (0.44–0.6)
Country					
American Samoa	35 (17–69)	141.42 (68.6–277.45)	80 (38–159)	152.3 (73.31–301.89)	0.19 (0.12–0.26)
Antigua and Barbuda	67 (32–132)	131.91 (63.78–256.72)	158 (78–311)	141.53 (69.6–279.91)	0.23 (0.22–0.24)
Arab Republic of Egypt	32464 (15559–63981)	111.09 (53.66–220.43)	87257 (42389–173548)	124 (60.23–249.48)	0.3 (0.28–0.33)
Argentine Republic	42867 (21102–83641)	131.87 (64.81–257.55)	80377 (39368–158658)	146.84 (71.81–289.41)	0.34 (0.3–0.38)
Australia	26596 (13086–52885)	137.84 (67.8–273.27)	64246 (32026–129852)	155.17 (76.55–312.08)	0.36 (0.34–0.39)
Barbados	379 (182–746)	140.14 (67.51–274.13)	745 (359–1459)	148.31 (71.51–289.94)	0.19 (0.18–0.21)
Belize	124 (61–240)	133.72 (65.68–258.22)	471 (230–917)	148.58 (72.5–292.35)	0.33 (0.27–0.38)
Bermuda	94 (46–184)	148.18 (72.65–291.34)	196 (95–391)	154.81 (75.32–307.35)	0.14 (0.13–0.15)
Bolivarian Republic of Venezuela	13309 (6484–26195)	132.91 (64.96–262.94)	44474 (21700–85825)	143.37 (69.93–277.15)	0.26 (0.25–0.27)
Bosnia and Herzegovina	3955 (1928–7750)	92 (45.14–180.9)	5971 (2935–11572)	99.6 (48.77–192.94)	0.29 (0.26–0.32)
Brunei Darussalam	189 (92–376)	172.54 (83.33–345.57)	721 (348–1382)	186.75 (91.37–362.74)	0.27 (0.26–0.29)
Burkina Faso	4888 (2355–9516)	108.49 (52.35–212.5)	11823 (5731–22510)	119.79 (58.26–230.29)	0.33 (0.32–0.34)
Canada	24628 (12065–48831)	77.19 (37.83–152.5)	58109 (28731–114079)	87.62 (43.44–170.24)	0.25 (0.16–0.34)
Central African Republic	1223 (603–2410)	100.89 (49.35–198.89)	2595 (1269–4953)	105.18 (51.7–204.41)	0.15 (0.14–0.15)
Commonwealth of Dominica	78 (38–149)	135.42 (66.32–256.41)	123 (59–236)	143.71 (69.89–277.48)	0.18 (0.15–0.21)
Commonwealth of the Bahamas	221 (107–428)	139.64 (67.87–269.85)	639 (314–1229)	148.02 (73.07–288)	0.19 (0.17–0.21)
Cook Islands	18 (9–35)	135.27 (65.62–263.93)	40 (19–77)	154.67 (75.91–304.3)	0.4 (0.36–0.45)
Czech Republic	13040 (6447–25248)	96.38 (47.48–186.09)	20512 (9962–40962)	103.48 (50.37–204.47)	0.23 (0.22–0.24)
Democratic People's Republic of Korea	26231 (12587–50986)	148.28 (71.75–285.53)	53199 (25426–102393)	154.48 (74.25–298.56)	0.16 (0.14–0.17)
Democratic Republic of Sao Tome and Principe	76 (37–152)	115.88 (56.6–231.87)	155 (75–303)	126.51 (61.91–248.59)	0.29 (0.28–0.29)
Democratic Republic of the Congo	17329 (8438–33566)	104.44 (51.04–204.1)	43277 (20996–84125)	107.62 (52.24–211.9)	0.06 (0.03–0.09)
Democratic Republic of Timor-Leste	297 (144–585)	90.19 (44.19–178.21)	841 (406–1634)	95.32 (46.14–185.94)	0.22 (0.2–0.24)
Democratic Socialist Republic of Sri Lanka	10915 (5295–21158)	94.33 (45.88–182.54)	28744 (14158–55010)	104.27 (51.49–200.02)	0.37 (0.36–0.38)
Dominican Republic	4900 (2391–9565)	128.27 (62.62–249.87)	14500 (7069–28749)	142.81 (69.86–283.56)	0.39 (0.37–0.4)
Eastern Republic of Uruguay	5086 (2502–10044)	133.26 (65.37–263.38)	7602 (3788–14814)	149.07 (73.92–289.97)	0.35 (0.32–0.39)
Federal Democratic Republic of Ethiopia	20700 (10087–40194)	99.51 (49.09–195.81)	49168 (24094–93721)	106.97 (52.87–205.47)	0.27 (0.25–0.29)
Federal Democratic Republic of Nepal	10624 (5170–20977)	105.06 (51.38–208.44)	29103 (14182–56926)	119.49 (58.27–234.68)	0.46 (0.43–0.48)
Federal Republic of Germany	156565 (77308–311069)	127.99 (63.16–252.34)	231584 (114594–458428)	134.22 (66.06–262.59)	0.15 (0.14–0.16)
Federal Republic of Nigeria	51197 (24886–98348)	111.53 (54.51–216.17)	122615 (59424–233522)	121.75 (59.65–234.6)	0.29 (0.2–0.38)
Federal Republic of Somalia	2724 (1321–5311)	102.64 (50.57–205.02)	7193 (3493–13865)	105.93 (51.62–208.03)	0.14 (0.13–0.15)
Federated States of Micronesia	63 (30–122)	128.31 (61.81–247.46)	117 (56–224)	141.4 (67.91–267.78)	0.27 (0.21–0.33)
Federative Republic of Brazil	116896 (57537–223889)	126.66 (62.65–244.8)	360985 (176855–696257)	141.08 (69.23–272.85)	0.38 (0.37–0.4)
French Republic	97176 (47476–191946)	123.22 (60.04–240.88)	162584 (79891–327792)	131.62 (64.49–260.94)	0.2 (0.19–0.22)
Gabonese Republic	638 (313–1249)	110.35 (54.12–216.44)	1415 (689–2761)	124.28 (60.69–244.7)	0.37 (0.35–0.39)
Georgia	5339 (2608–10222)	84.64 (41.16–161.76)	5020 (2480–9878)	87.14 (42.82–169.53)	0.11 (0.1–0.12)
Grand Duchy of Luxembourg	679 (337–1377)	126.72 (62.78–257.06)	1359 (674–2689)	133.32 (65.96–263.44)	0.16 (0.14–0.17)
Greenland	31 (15–60)	84.55 (41.86–167.5)	69 (33–134)	94.62 (45.73–182.76)	0.37 (0.34–0.4)
Grenada	87 (43–170)	129.16 (63.41–249.82)	167 (81–333)	140.54 (68.28–281.36)	0.27 (0.24–0.3)
Guam	116 (55–227)	135.64 (65.82–265.53)	320 (157–624)	152.14 (74.43–296.92)	0.38 (0.36–0.41)
Hashemite Kingdom of Jordan	1620 (784–3082)	111.52 (54.28–215.82)	10702 (5239–20354)	125.81 (61.49–243.6)	0.42 (0.4–0.44)
Hellenic Republic	18833 (9078–37247)	125.79 (60.91–247.97)	28081 (13784–56505)	134.25 (65.69–267.61)	0.19 (0.16–0.22)
Hungary	14369 (7077–28081)	98.84 (48.48–193.09)	19055 (9423–36956)	105.11 (51.7–203.86)	0.21 (0.2–0.22)
Independent State of Papua New Guinea	2303 (1086–4470)	114.97 (54.57–226.46)	7264 (3474–14185)	123.06 (59.44–240.74)	0.18 (0.16–0.2)
Independent State of Samoa	119 (58–227)	134.29 (65–254.58)	219 (106–426)	143.09 (69.75–277.26)	0.15 (0.11–0.2)
Ireland	5014 (2457–10022)	125.9 (61.73–250.72)	10195 (5030–20164)	134.37 (66.12–264.26)	0.21 (0.2–0.22)
Islamic Republic of Afghanistan	6976 (3404–13566)	96.96 (47.77–190.35)	11622 (5532–22727)	105.83 (51.96–207.18)	0.38 (0.34–0.42)
Islamic Republic of Iran	28360 (13831–54075)	103.01 (50.59–198.93)	94081 (46049–179997)	114.33 (56.35–220.61)	0.33 (0.32–0.35)
Islamic Republic of Mauritania	1211 (589–2364)	118.4 (57.85–231.98)	2963 (1417–5710)	129.86 (62.52–251.13)	0.27 (0.26–0.29)
Islamic Republic of Pakistan	52639 (25729–102844)	89.99 (44.24–175.78)	136538 (65620–267834)	102.1 (49.84–199.58)	0.49 (0.44–0.53)
Jamaica	2268 (1099–4466)	132.05 (63.74–259.51)	4433 (2160–8863)	143.46 (69.88–286.97)	0.3 (0.28–0.33)
Japan	294380 (143191–572927)	170.73 (83.19–332.69)	516730 (252520–1038663)	172.94 (84.12–338.87)	0.05 (0.03–0.07)
Kingdom of Bahrain	237 (114–476)	114.99 (55.66–230.23)	1537 (739–2958)	126.03 (61.59–245.21)	0.3 (0.29–0.31)
Kingdom of Belgium	18380 (8925–36700)	124.42 (60.17–247.59)	27736 (13568–55345)	131.97 (64.77–261.92)	0.17 (0.16–0.18)
Kingdom of Bhutan	295 (141–567)	111.65 (54.48–215.24)	788 (387–1565)	124.77 (61.28–246.23)	0.39 (0.38–0.41)
Kingdom of Cambodia	4482 (2151–8704)	92.91 (44.91–179.13)	13447 (6479–26600)	100.75 (48.88–200.81)	0.29 (0.28–0.3)
Kingdom of Denmark	9137 (4518–18175)	119.5 (58.85–234.23)	13922 (6826–28168)	128.83 (62.84–261.27)	0.23 (0.2–0.27)
Kingdom of Eswatini	366 (177–705)	119.76 (58.51–231.04)	765 (380–1510)	127.3 (63.27–252.58)	0.14 (0.08–0.19)
Kingdom of Lesotho	947 (473–1811)	109.22 (54.81–210.34)	1327 (650–2590)	118.07 (58.19–233.04)	0.25 (0.24–0.26)
Kingdom of Morocco	15303 (7421–29900)	105.07 (51.24–204.98)	42500 (20497–82095)	117.61 (56.81–228.84)	0.38 (0.37–0.38)
Kingdom of Norway	7579 (3725–15067)	120.73 (59.02–235.92)	11935 (5873–23859)	129.02 (63.15–255.23)	0.23 (0.22–0.25)
Kingdom of Saudi Arabia	6951 (3314–13195)	108.42 (52.19–208.21)	31919 (15257–62521)	127.17 (62.02–250.77)	0.52 (0.49–0.54)
Kingdom of Spain	67480 (32731–134997)	126.8 (61.43–252.33)	119290 (58113–239202)	134.64 (65.43–268.95)	0.17 (0.15–0.19)
Kingdom of Sweden	13756 (6680–27910)	98.45 (47.81–198.19)	20366 (9790–40597)	106.73 (51.7–214.8)	0.2 (0.11–0.3)
Kingdom of Thailand	39261 (19076–76952)	101.46 (50.05–200.06)	132566 (63764–257908)	120.3 (57.88–234.65)	0.61 (0.59–0.63)
Kingdom of the Netherlands	25516 (12402–50214)	131.87 (63.9–257.97)	45063 (21860–92076)	138.94 (67.19–282.57)	0.16 (0.15–0.18)
Kingdom of Tonga	77 (37–154)	132.73 (64.24–265.25)	121 (59–241)	146.33 (71.63–291.56)	0.24 (0.16–0.31)
Kyrgyz Republic	2375 (1140–4601)	79.69 (38.5–155.89)	4261 (2026–8187)	83.75 (40.22–161.24)	0.16 (0.15–0.18)
Lao People's Democratic Republic	2033 (979–3936)	93.09 (45.38–180.88)	5223 (2514–10115)	102.16 (49.49–197.59)	0.36 (0.34–0.37)
Lebanese Republic	2439 (1199–4631)	107.47 (52.73–205.41)	7086 (3490–13692)	121.42 (59.85–233.55)	0.39 (0.33–0.44)
Malaysia	9917 (4789–19174)	99.19 (48.16–190.3)	33272 (16079–65244)	111.34 (53.97–217.89)	0.42 (0.41–0.44)
Mongolia	829 (402–1595)	78.35 (38.19–151.98)	2092 (1020–3953)	83.38 (40.98–162.01)	0.09 (0–0.17)
Montenegro	628 (309–1211)	98.77 (48.46–191.43)	999 (491–1957)	103 (50.62–201.4)	0.17 (0.15–0.19)
New Zealand	5155 (2544–10185)	134.3 (66.21–264.63)	11881 (5739–23580)	149.04 (71.52–294.32)	0.33 (0.31–0.36)
North Macedonia	1826 (891–3578)	94.7 (46.48–186.89)	3393 (1658–6626)	100.67 (49.45–196.81)	0.23 (0.22–0.23)
Northern Mariana Islands	30 (14–58)	134.16 (65.08–265.08)	89 (43–173)	147.76 (71.27–287.35)	0.26 (0.2–0.32)
Palestine	970 (472–1853)	109.7 (53.42–211.32)	3296 (1589–6374)	116.88 (57.52–226.16)	0.18 (0.16–0.2)
People's Democratic Republic of Algeria	13658 (6638–26825)	106.71 (52.1–209.37)	46474 (22727–90485)	121.61 (59.51–238.67)	0.44 (0.43–0.46)
People's Republic of Bangladesh	51711 (25008–101213)	104.5 (50.94–204.91)	165130 (80996–320139)	114.67 (56.31–222.46)	0.34 (0.32–0.36)
People's Republic of China	1339566 (644356–2583887)	151.24 (72.96–291.47)	3554153 (1715777–6842994)	162.44 (78.35–314.13)	0.5 (0.39–0.62)
Plurinational State of Bolivia	4218 (2051–8362)	126.93 (62.35–251.08)	13606 (6634–26815)	142.87 (70.17–281.93)	0.4 (0.38–0.41)
Portuguese Republic	17039 (8321–33912)	123.68 (60.43–245.07)	29502 (14316–59595)	134.19 (65.21–267.76)	0.23 (0.2–0.25)
Principality of Andorra	73 (36–144)	124.16 (60.92–245.86)	204 (99–406)	133.69 (64.59–266.35)	0.25 (0.22–0.27)
Principality of Monaco	84 (41–167)	132.69 (65.63–264.31)	120 (59–238)	139.02 (68.03–272.43)	0.14 (0.13–0.15)
Puerto Rico	5266 (2559–10274)	145.89 (70.96–283.53)	10051 (4946–19810)	158.99 (77.74–309.27)	0.32 (0.29–0.34)
Republic of Albania	1951 (944–3787)	91.22 (44.58–178.69)	4253 (2042–8595)	99.3 (47.97–200.61)	0.32 (0.31–0.33)
Republic of Angola	4258 (2049–8136)	101.5 (49.6–197.92)	14606 (7070–27998)	111.34 (54.45–215.46)	0.32 (0.3–0.34)
Republic of Armenia	2213 (1081–4257)	79.14 (38.58–153.17)	3722 (1843–7312)	86.74 (42.89–170.72)	0.33 (0.32–0.35)
Republic of Austria	14228 (6962–28344)	126.03 (61.55–249.93)	22227 (10965–44212)	133.91 (65.83–264.35)	0.18 (0.17–0.19)
Republic of Azerbaijan	4124 (2003–8076)	81.11 (39.88–160.53)	9708 (4742–18495)	88.42 (43.31–170.71)	0.33 (0.3–0.36)
Republic of Belarus	13245 (6472–26018)	101.92 (49.76–200.77)	17264 (8318–34019)	109.69 (52.83–216.39)	0.28 (0.25–0.3)
Republic of Benin	2259 (1080–4411)	111.44 (53.53–217.93)	6984 (3408–13593)	125.07 (61.82–244.36)	0.38 (0.35–0.4)
Republic of Botswana	643 (307–1239)	109.93 (52.91–214.52)	1992 (975–3940)	123.98 (60.59–248.1)	0.4 (0.38–0.41)
Republic of Bulgaria	12290 (5937–23729)	98.29 (47.48–189.85)	13728 (6750–26930)	102.7 (50.41–198.79)	0.14 (0.12–0.16)
Republic of Burundi	2427 (1190–4619)	102.31 (50.09–195.85)	5531 (2654–10555)	104.18 (50.29–200.07)	0.07 (0.07–0.08)
Republic of Cabo Verde	251 (123–489)	115.08 (56.15–222.38)	594 (289–1151)	127.43 (61.88–247.86)	0.33 (0.32–0.34)
Republic of Cameroon	5707 (2765–10896)	120.72 (58.75–231.11)	17951 (8569–34980)	130.11 (62.58–254.99)	0.22 (0.21–0.24)
Republic of Chad	3031 (1466–5912)	106.11 (51.56–207.61)	6988 (3409–13443)	111.48 (54.86–216.46)	0.15 (0.14–0.15)
Republic of Chile	13904 (6722–27550)	135.77 (65.85–269.09)	38234 (18693–77073)	150.69 (73.39–303.93)	0.32 (0.29–0.36)
Republic of Colombia	23527 (11385–45058)	128.55 (61.96–247.24)	79043 (38116–153867)	142.58 (68.71–277.9)	0.34 (0.33–0.35)
Republic of Costa Rica	2357 (1142–4651)	132.93 (64.61–262.42)	8041 (3923–15769)	145.48 (70.95–286.03)	0.3 (0.29–0.31)
Republic of Croatia	6023 (2958–11892)	96.25 (47.15–190.95)	8419 (4105–16399)	102.96 (50.42–198.34)	0.26 (0.25–0.28)
Republic of Cuba	13453 (6513–26695)	131.47 (63.65–260.31)	27640 (13579–55736)	144.45 (70.86–292.05)	0.34 (0.33–0.36)
Republic of Cyprus	992 (488–1947)	119.45 (59.04–234.05)	2630 (1307–5124)	129.59 (64.08–251.31)	0.28 (0.26–0.3)
Republic of C么te d'Ivoire	4938 (2382–9735)	111.88 (54.46–222.52)	15273 (7401–30189)	122.09 (59.48–241.55)	0.25 (0.24–0.27)
Republic of Djibouti	157 (75–304)	102.75 (49.99–197.28)	832 (404–1615)	113.56 (55.49–223.9)	0.37 (0.36–0.39)
Republic of Ecuador	7494 (3696–14624)	137.45 (67.85–268.19)	25363 (12259–48621)	152.58 (73.79–292.4)	0.4 (0.37–0.42)
Republic of El Salvador	3903 (1905–7522)	130.08 (63.64–250.97)	8819 (4302–17539)	145.01 (70.69–288.1)	0.36 (0.32–0.39)
Republic of Equatorial Guinea	207 (99–402)	101.7 (49.27–199.52)	689 (335–1341)	122.09 (60.4–237.39)	0.71 (0.67–0.76)
Republic of Estonia	2113 (1020–4122)	103.27 (50.1–201.72)	2727 (1323–5331)	112.49 (54.32–218.33)	0.33 (0.31–0.35)
Republic of Fiji	516 (248–1000)	127.98 (62.29–248.13)	1217 (596–2330)	144.93 (71.49–278.4)	0.36 (0.32–0.4)
Republic of Finland	8807 (4384–17434)	126.59 (62.86–249.24)	14890 (7217–29729)	133.99 (64.33–264.62)	0.19 (0.18–0.2)
Republic of Ghana	7406 (3558–14249)	111.67 (54.02–215.93)	23295 (11262–44778)	127.8 (62.24–247.63)	0.43 (0.42–0.44)
Republic of Guatemala	4462 (2135–8842)	122.05 (59.02–241.98)	15231 (7434–30404)	134.63 (65.58–267.88)	0.31 (0.3–0.33)
Republic of Guinea	3717 (1840–7210)	109.9 (54.44–212.81)	6950 (3385–13372)	117.14 (57.38–226.26)	0.19 (0.18–0.2)
Republic of Guinea-Bissau	453 (222–870)	109.81 (54.47–211.5)	932 (451–1817)	116.95 (57.15–229.79)	0.18 (0.17–0.19)
Republic of Guyana	498 (242–998)	127.02 (61.7–256.03)	939 (460–1852)	138.72 (67.86–274.75)	0.29 (0.27–0.32)
Republic of Haiti	3961 (1934–7670)	118.05 (57.89–229.61)	9660 (4750–18354)	124.28 (61.81–239.32)	0.2 (0.19–0.21)
Republic of Honduras	2650 (1300–5101)	125.32 (61.38–240.79)	9160 (4487–17958)	137.46 (67.67–270.02)	0.31 (0.28–0.33)
Republic of Iceland	351 (172–694)	127.75 (62.62–250.99)	744 (364–1467)	136.89 (67.2–268.07)	0.24 (0.23–0.24)
Republic of India	554440 (269183–1064069)	111.3 (54.46–215.39)	1540454 (751878–2951122)	123.57 (60.56–237.59)	0.37 (0.36–0.38)
Republic of Indonesia	98165 (47713–190495)	92.39 (45–178.39)	275146 (132684–526765)	102.61 (49.97–197.86)	0.39 (0.37–0.4)
Republic of Iraq	9253 (4558–17759)	114 (56.03–219.58)	31602 (15422–62091)	119.84 (58.95–234.94)	0.18 (0.16–0.2)
Republic of Italy	107085 (52915–211524)	123.85 (61.14–243.49)	167636 (82461–335989)	130.44 (63.53–257.36)	0.16 (0.14–0.17)
Republic of Kazakhstan	10801 (5193–20651)	84.36 (40.71–162.94)	16847 (8206–33048)	90.33 (44.02–179.02)	0.22 (0.21–0.24)
Republic of Kenya	8940 (4362–17118)	104.62 (51.39–202.65)	28266 (13707–54094)	113.89 (55.72–220.76)	0.3 (0.27–0.33)
Republic of Kiribati	50 (24–96)	126.9 (62.27–244)	112 (55–216)	139.85 (68.27–269.4)	0.27 (0.21–0.33)
Republic of Korea	61765 (29630–120272)	197.92 (96.25–386.35)	187910 (91977–373887)	199.93 (97.42–398.67)	0.21 (0.08–0.34)
Republic of Latvia	3717 (1826–7249)	103.97 (51.11–202.53)	4041 (2014–8014)	111.88 (55.63–219.71)	0.28 (0.26–0.29)
Republic of Liberia	1318 (634–2527)	112.6 (54.35–216.04)	2963 (1449–5771)	123.29 (60.5–241.95)	0.38 (0.34–0.42)
Republic of Lithuania	4657 (2299–9237)	103.12 (50.89–204.98)	5839 (2848–11480)	110.56 (53.62–215.33)	0.26 (0.24–0.27)
Republic of Madagascar	5323 (2571–10251)	101.35 (49.45–197.46)	13393 (6533–25563)	106.28 (51.99–207.17)	0.19 (0.18–0.2)
Republic of Malawi	4195 (2016–8074)	104.29 (50.67–200.63)	8934 (4421–17114)	112.56 (55.4–217.43)	0.3 (0.28–0.32)
Republic of Maldives	91 (44–177)	90.31 (43.86–173.83)	419 (200–815)	104.98 (50.84–202.31)	0.59 (0.56–0.63)
Republic of Mali	4503 (2209–8692)	107.14 (52.75–204.88)	10970 (5293–20862)	115.31 (56.18–220.97)	0.25 (0.25–0.26)
Republic of Malta	533 (266–1054)	124.43 (62.24–246.46)	1174 (577–2331)	133.51 (64.92–266.47)	0.21 (0.17–0.25)
Republic of Mauritius	763 (369–1481)	100.54 (48.73–195.43)	2090 (1036–4020)	111.59 (55.51–214.8)	0.36 (0.35–0.38)
Republic of Moldova	4434 (2171–8657)	99.32 (48.9–195.9)	6424 (3145–12500)	109.69 (53.78–213.18)	0.39 (0.35–0.43)
Republic of Mozambique	6460 (3093–12550)	101.58 (49.22–199.02)	13149 (6444–25044)	108.99 (53.6–209.25)	0.27 (0.26–0.29)
Republic of Namibia	736 (353–1451)	109.26 (52.82–216.56)	1732 (842–3385)	117.45 (57.52–231.29)	0.22 (0.21–0.23)
Republic of Nauru	6 (3–12)	126.88 (62.16–242.36)	9 (4–18)	144.83 (70.35–281.7)	0.38 (0.35–0.4)
Republic of Nicaragua	2024 (990–3869)	127.34 (62.35–245.13)	7141 (3503–13903)	140.24 (69–274.93)	0.32 (0.29–0.34)
Republic of Niue	3 (1–6)	134.82 (64.71–265.88)	3 (2–6)	150.68 (72.51–287.76)	0.35 (0.3–0.39)
Republic of Palau	14 (7–27)	134.79 (65.18–262.44)	38 (19–74)	147.83 (72.21–287.35)	0.25 (0.21–0.3)
Republic of Panama	1879 (905–3661)	123.81 (59.76–242.05)	6223 (3041–12053)	140.61 (68.7–272.54)	0.39 (0.38–0.4)
Republic of Paraguay	2933 (1432–5722)	129.69 (63.52–253.77)	8398 (4150–16404)	140.18 (69.46–274.98)	0.26 (0.25–0.27)
Republic of Peru	15928 (7860–30926)	129.6 (64.08–252.92)	49906 (24296–97319)	146.4 (71.47–285.33)	0.43 (0.41–0.44)
Republic of Poland	41229 (20378–80488)	94.48 (46.75–184.66)	69832 (34359–136543)	103.31 (50.64–200.25)	0.3 (0.3–0.31)
Republic of Rwanda	3055 (1473–5922)	104.29 (50.67–203.61)	7626 (3725–14853)	111.54 (55.03–214.75)	0.26 (0.24–0.28)
Republic of San Marino	43 (21–85)	128.59 (63.16–252.68)	90 (45–182)	136.2 (67.19–271.18)	0.18 (0.16–0.2)
Republic of Senegal	3798 (1835–7336)	113.44 (55.29–219.71)	10001 (4860–19126)	121.3 (58.77–232.07)	0.18 (0.17–0.19)
Republic of Serbia	11294 (5516–21504)	96.52 (47.19–186.17)	16014 (7794–31639)	103.12 (50.21–203.02)	0.25 (0.24–0.27)
Republic of Seychelles	58 (28–114)	105.2 (51.47–205.85)	142 (68–276)	112.46 (54.22–219.88)	0.22 (0.21–0.24)
Republic of Sierra Leone	2266 (1093–4415)	109.01 (52.82–213.95)	4735 (2272–9169)	117.2 (56.65–228.2)	0.23 (0.22–0.24)
Republic of Singapore	4187 (2003–8083)	180.34 (86.99–351.05)	16639 (7934–32771)	189.49 (90.34–372.91)	0.14 (0.13–0.16)
Republic of Slovenia	2369 (1149–4716)	96.37 (46.75–192.03)	4179 (2072–8307)	103.56 (51.21–202.39)	0.25 (0.24–0.26)
Republic of South Africa	24820 (12118–47792)	117.57 (57.7–228.06)	60816 (29661–117281)	125.33 (61.47–243.88)	0.23 (0.22–0.24)
Republic of South Sudan	2660 (1284–5079)	101.7 (49.36–195.19)	4728 (2321–9021)	108.57 (53.63–210.57)	0.24 (0.23–0.25)
Republic of Sudan	9404 (4557–18194)	98.4 (47.9–191.72)	24475 (12017–47106)	114.81 (56.48–221.5)	0.53 (0.5–0.56)
Republic of Suriname	348 (169–675)	131.82 (63.96–258.14)	944 (466–1810)	144.22 (71.03–278.43)	0.32 (0.31–0.33)
Republic of Tajikistan	2087 (994–4133)	75.86 (36.2–151.75)	4935 (2417–9778)	78.21 (38.74–156.27)	0.1 (0.08–0.12)
Republic of the Congo	1204 (586–2304)	108.56 (52.87–209.68)	3541 (1714–6933)	116.57 (56.92–230.51)	0.24 (0.23–0.24)
Republic of the Gambia	412 (201–785)	111.38 (55.06–214.32)	1263 (605–2394)	121.78 (58.9–232.44)	0.28 (0.27–0.29)
Republic of the Marshall Islands	20 (10–38)	119.57 (58.23–225.98)	53 (25–104)	131.8 (63.07–260.89)	0.27 (0.24–0.29)
Republic of the Niger	3199 (1549–6242)	107.11 (52.02–208.68)	10114 (4890–19032)	113.83 (55.26–216.92)	0.2 (0.19–0.2)
Republic of the Philippines	26112 (12706–50350)	81.92 (40.04–158.07)	82832 (40462–158988)	93.83 (45.87–179.59)	0.46 (0.42–0.49)
Republic of the Union of Myanmar	22417 (10830–43769)	92.39 (44.91–179.51)	53872 (26738–102576)	103.12 (51.32–197.63)	0.42 (0.4–0.44)
Republic of Trinidad and Tobago	1155 (566–2241)	135.7 (66.65–262.31)	2853 (1394–5679)	146.43 (71.6–292.18)	0.31 (0.28–0.33)
Republic of Tunisia	5599 (2748–10882)	107.73 (52.96–211.4)	16486 (7940–31816)	119.54 (57.56–232.17)	0.36 (0.35–0.37)
Republic of Turkey	42192 (20395–81566)	116.38 (56.6–227.36)	126271 (61284–245051)	130.19 (63.25–252.87)	0.37 (0.35–0.39)
Republic of Uganda	6824 (3321–13180)	102.01 (49.71–200.01)	17661 (8570–34096)	110.69 (54.32–216.01)	0.31 (0.29–0.32)
Republic of Uzbekistan	9159 (4465–17938)	79.51 (38.62–156.43)	24032 (11727–46756)	85.96 (42.01–169.34)	0.26 (0.25–0.28)
Republic of Vanuatu	83 (40–162)	120.78 (59.04–236.49)	260 (126–502)	134.2 (65.81–261.1)	0.33 (0.31–0.35)
Republic of Yemen	4937 (2412–9637)	96.03 (47.21–188.3)	16134 (7868–31295)	105.24 (51.68–203.52)	0.33 (0.31–0.34)
Republic of Zambia	3179 (1523–6160)	105.64 (50.92–208.6)	8609 (4213–16474)	112.62 (55.14–218.85)	0.23 (0.2–0.25)
Republic of Zimbabwe	4701 (2286–9092)	110.49 (54.14–215.59)	8507 (4101–16584)	113.71 (55.7–220.1)	0.06 (0.03–0.08)
Romania	26849 (13167–52774)	94.77 (46.67–187.23)	36137 (17727–71550)	103.48 (50.64–202.78)	0.32 (0.31–0.33)
Russian Federation	180978 (88673–350851)	99.62 (49–194.07)	256203 (126319–498011)	108.83 (53.62–211.13)	0.33 (0.31–0.35)
Saint Kitts and Nevis	48 (23–93)	135.65 (66.11–261.08)	111 (54–216)	147.7 (72.28–291.17)	0.27 (0.26–0.29)
Saint Lucia	114 (56–224)	131.05 (64.84–257.19)	352 (171–694)	143.48 (69.78–283.36)	0.28 (0.26–0.3)
Saint Vincent and the Grenadines	90 (44–178)	129.15 (62.66–253.02)	206 (100–403)	141.03 (68.78–277.31)	0.32 (0.31–0.34)
Slovak Republic	5730 (2815–11121)	96.74 (47.49–187.92)	9556 (4709–18597)	103.69 (50.99–200.43)	0.21 (0.19–0.22)
Socialist Republic of Viet Nam	37511 (18251–72616)	93 (45.39–181.17)	107216 (51571–209203)	100.6 (49–196.41)	0.3 (0.28–0.31)
Solomon Islands	177 (85–339)	117.49 (57.54–227.46)	513 (247–1001)	131.46 (63.51–255.93)	0.32 (0.27–0.37)
State of Eritrea	1243 (588–2460)	99.67 (47.89–193.72)	3228 (1551–6295)	104.94 (50.63–207.61)	0.17 (0.15–0.19)
State of Israel	5920 (2878–11919)	124.69 (60.39–249.91)	15676 (7704–31340)	134.15 (65.8–266.51)	0.21 (0.18–0.23)
State of Kuwait	839 (409–1662)	116.81 (56.51–231.36)	4922 (2387–9623)	130.5 (64.01–254.71)	0.42 (0.4–0.44)
State of Libya	2242 (1092–4295)	113.83 (55.98–218.17)	7281 (3550–14073)	123.25 (60.64–238.55)	0.27 (0.26–0.29)
State of Qatar	192 (92–383)	117.44 (57.49–223.99)	2109 (1012–4122)	130.29 (64.19–247.63)	0.31 (0.29–0.33)
Sultanate of Oman	769 (370–1511)	102.02 (49.85–199.97)	3173 (1537–6083)	124.08 (61.38–237.17)	0.68 (0.66–0.7)
Swiss Confederation	12251 (5979–24388)	124.04 (60.65–244.74)	21202 (10378–42190)	129.08 (62.8–254.19)	0.14 (0.13–0.15)
Syrian Arab Republic	5932 (2875–11318)	108.53 (53.24–209.47)	17350 (8395–32825)	119 (57.84–227.04)	0.29 (0.26–0.31)
Taiwan (Province of China)	24851 (11783–48933)	148.97 (70.96–294.47)	70271 (33639–136870)	170.98 (81.66–333.52)	0.52 (0.49–0.55)
Togolese Republic	1457 (706–2805)	110.53 (53.52–211.83)	5125 (2442–9961)	120.19 (57.73–233.64)	0.26 (0.24–0.27)
Tokelau	2 (1–3)	127.14 (62.02–249.92)	2 (1–4)	143.77 (70.83–276.03)	0.38 (0.35–0.42)
Turkmenistan	1560 (759–3037)	80.99 (39.57–157.96)	3733 (1820–7344)	88.18 (43.22–175.04)	0.3 (0.28–0.31)
Tuvalu	9 (4–17)	127.3 (61.5–247.05)	15 (7–30)	140.5 (68.27–278.79)	0.29 (0.25–0.33)
Ukraine	72447 (35302–142316)	101.08 (49.27–199.33)	80151 (39438–154260)	105.89 (52.12–202.91)	0.19 (0.17–0.21)
Union of the Comoros	219 (107–425)	105.7 (51.83–205.55)	586 (283–1108)	112.46 (54.91–215.07)	0.22 (0.21–0.22)
United Arab Emirates	680 (325–1324)	110.22 (53.78–213.6)	9961 (4799–19487)	123.92 (60.68–242.4)	0.41 (0.39–0.43)
United Kingdom of Great Britain and Northern Ireland	114621 (56460–228189)	134.22 (65.93–264.35)	170266 (84003–337772)	143.71 (70.54–281.89)	0.27 (0.2–0.34)
United Mexican States	59048 (28894–114045)	133.77 (65.76–260.27)	190226 (93127–365853)	145.33 (71.36–280.98)	0.26 (0.25–0.28)
United Republic of Tanzania	11934 (5752–22969)	105.63 (51.31–204.52)	31518 (15103–60497)	114.37 (55.06–222.36)	0.29 (0.28–0.3)
United States of America	457798 (225498–909620)	151.02 (74.13–298.17)	853428 (420640–1694293)	156.32 (76.78–309.62)	−0.15 (−0.35–0.05)
United States Virgin Islands	131 (63–255)	144.74 (70.62–282.31)	269 (132–522)	155.25 (76.17–299.19)	0.23 (0.21–0.26)

Gender-specific analysis revealed that in 2021, the number of incidence, prevalence, and DALYs counts, along with their respective ASRs, were higher in females compared to males (Figure S1, Tables 1, 2, and 3). An age-stratified analysis of incidence, prevalence, and DALYs in 2021 is presented in Figure S1. For the the number of cases and corresponding ASRs, there was an initial increase with age, peaking in around older years old, followed by a decrease (Figure S1, Tables 1, 2, and 3).

**Fig. 1:**
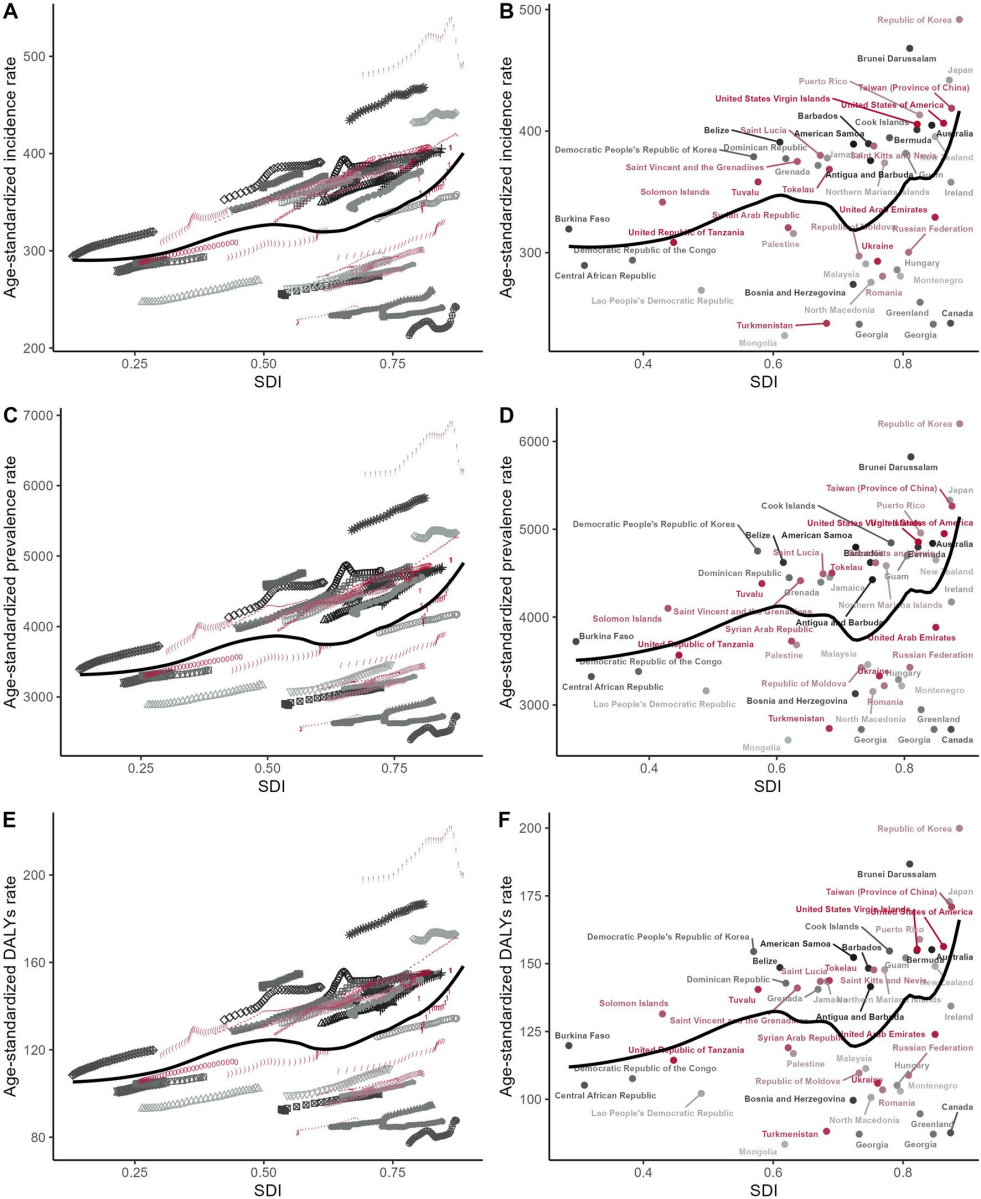
Age-standardized rates of incidence, prevalence, and DALYs attributable to osteoarthritis knee across countries and territories by socio-demographic index for both sexes, 1990–2019. The black line was an adaptive association fitted with adaptive Loess regression based on all data points. Abbreviations: DALYs, disability-adjusted-life-years

At the SDI region level, the high SDI region had the most ASIR at 386.58 (95% UI: 334.58–439.34), ASPR at 4656.7 (95% UI: 4033.58–5313.55), and the age-standardized DALYs rate (ASDAR) at 149.14 (95% UI: 73.09–292.89). For the number of incidence, prevalence, and DALYs cases, the disease burden was highest in the middle SDI regions (Figure S1, Tables 1, 2, and 3). Moreover, the region with the lowest disease burden was low SDI regions. The regular pattern of different SDI levels and disease burden was stable across countries and territories. From Fig. 1, the ASRs for incidence, prevalence, and DALYs showed an “J” relationship with SDI, where ASRs increase with SDI (Fig. 1).

Across the 54 GBD regions, High-income Asia Pacific ranked the top one in osteoarthritis knee-related ASIR (458.22, 95% UI: 397.65–522.59), ASPR (5573.73, 95% UI: 4841.29–6334.33), and ASDAR (180.61, 95% UI: 88.02–356.06), followed by East Asia. Meanwhile, Central Asia ranked the bottom one (ASIR: 239.29, 95% UI: 205.97–274.09; ASPR: 239.29, 95% UI: 205.97–274.09; ASDAR: 86.71, 95% UI: 42.6–169.71). For the number of cases, the top GBD region was Asia (incidence: 18.94 million, 95% UI: 16.33–21.70; prevalence: 229.88 million, 95% UI: 197.01–263.57; DALYs: 7.41 million, 95% UI: 3.58–14.27), followed by Basic Health System and World Bank Upper Middle Income. The bottom was Oceania for incidence, prevalence, and DALYs (incidence: 33635, 95% UI: 28494–39060; prevalence: 338675, 95% UI: 285507–391076; DALYs: 10965, 95% UI: 5248–21272) (Figure S1, Tables 1, 2, and 3).

**Fig. 2:**
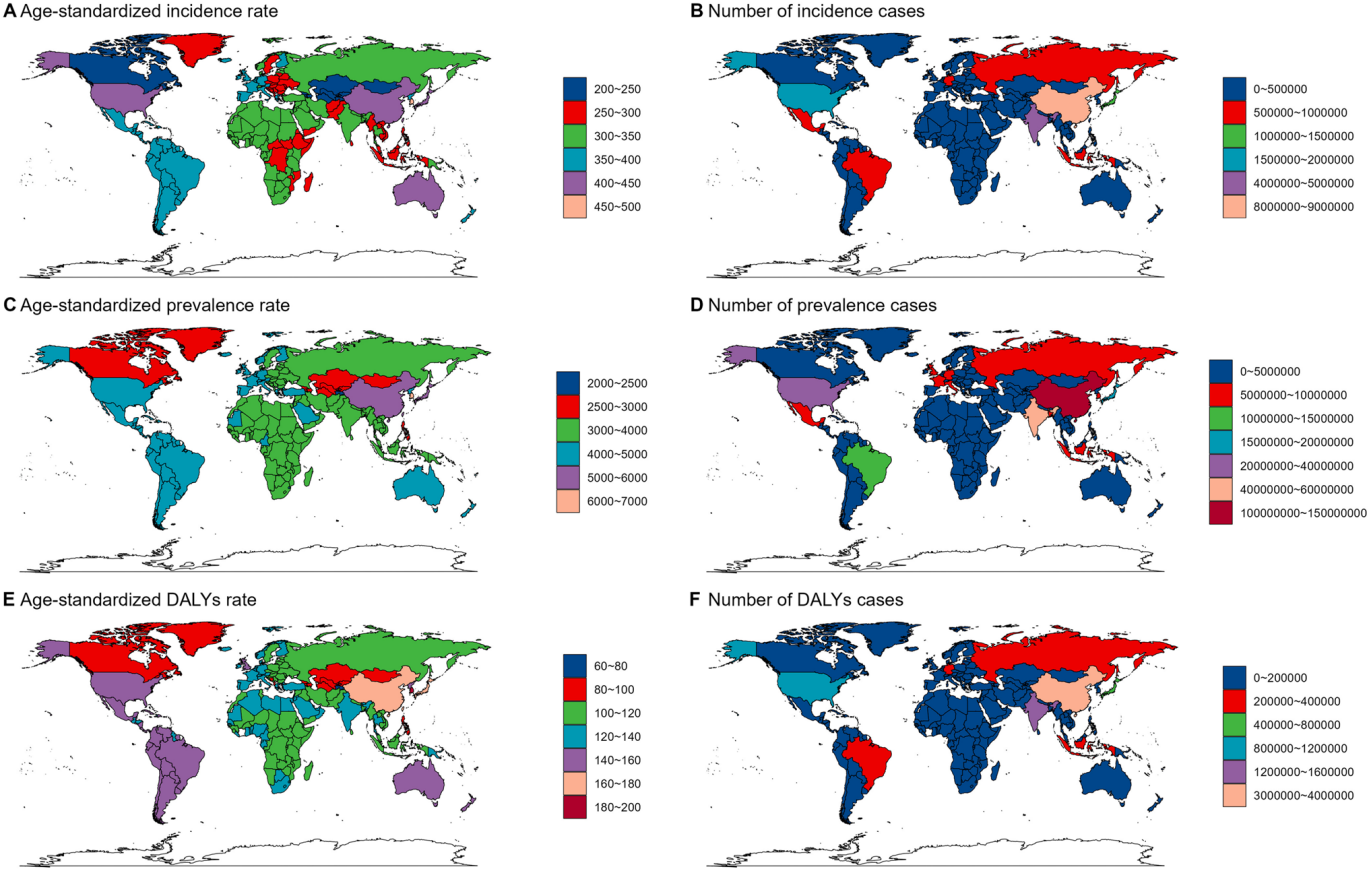
Numbers and age-standardized rates of osteoarthritis knee-related incidence, prevalence, and DALYs across countries and territories in 2021. Abbreviations: DALYs, disability-adjusted life years

The disease burden of osteoarthritis knee varied considerably across the world. For number of cases, the top number of incidence cases, prevalence cases, and DALYs cases were observed in People's Republic of China (incidence: 8.51 million, 95% UI: 7.28–9.84; prevalence: 109.58 million, 95% UI: 92.72–126.64; DALYs: 3.55 million, 95% UI: 1.72–6.84) in 2021, followed by Republic of India and United States of America. Moreover, the bottom number of cases were observed in Tokelau (incidence: 5, 95% UI: 5–6; prevalence: 66, 95% UI: 57–76; DALYs: 2, 95% UI: 1–4), followed by Republic of Niue and Republic of Nauru. As for the ASRs, the highest ASIR, ASPR, and ASDAR was observed in Republic of Korea (ASIR: 491.74, 95% UI: 427.19–560.51; ASPR: 6201.62, 95% UI: 5389.51–7093.43; ASDAR: 199.93, 95% UI: 97.42–398.67). And the lowest was observed in Republic of Tajikistan (ASIR: 218.48, 95% UI: 189.9–250.18; ASPR: 2425.49, 95% UI: 2101.17–2773.29; ASDAR: 78.21, 95% UI: 38.74–156.27) (Fig. 2, Tables 1, 2, and 3).

### Temporal trend for osteoarthritis knee-related disease burden from 1990 to 2021

**Fig. 3:**
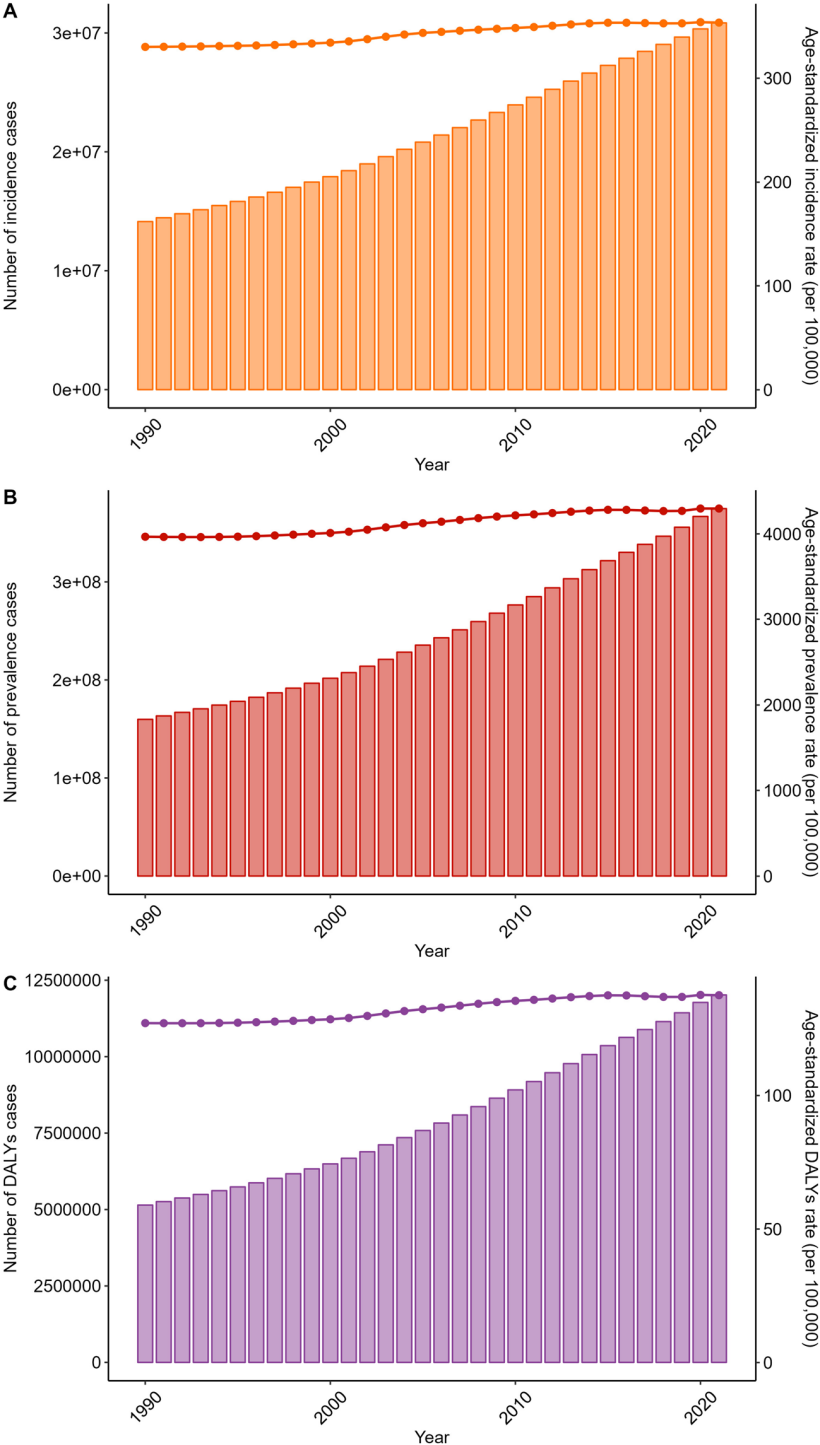
Trends in the numbers and age-standardized rates of osteoarthritis knee-related incidence, prevalence, and DALYs globally from 1990 to 2021. Abbreviations: DALYs, disability-adjusted-life-years

Globally, the number of cases of osteoarthritis knee has showed a increasing trend from 1990 to 2021. The corresponding ASRs demonstrated the same trend. The number of incidence cases increased from 14.13 million (95% UI: 12.15–16.08) to 30.85 million (95% UI: 26.53–35.19), and the ASIR increased from 330.26 (95% UI: 284.34–375.75) to 353.67 (95% UI: 304.56–402.5). The number of prevalence cases increased from 159.80 million (95% UI: 137.28–182.88) to 374.74 million (95% UI: 321.86–428.35), and the ASPR increased from 3964.75 (95% UI: 3411.86–4536.4) to 4294.27 (95% UI: 3695.04–4910.76). And the number of DALYs cases increased from 5.15 million (95% UI: 2.51–9.95) to 12.02 million (95% UI: 5.86–23.27), while the ASR of DALYs increased from 127.14 (95% UI: 62.17–246.99) to 137.59 (95% UI: 67.08–266.87) (Fig. 3, Tables 1, 2, and 3).

The trends observed in both sexes were found to be congruent with those of the overall population, suggesting a uniformity in the disease dynamics across this demographic subgroup (Figure S2, Tables 1, 2, and 3). The disease burden for most age groups also revealed same trend (Figure S2, Tables 1, 2, and 3).

At the SDI regional level, our analysis reveals a pattern in the trends of osteoarthritis knee indicators. For the ASRs and number of cases, all SDI regions showed the same trend as the overall population (Figure S2, Tables 1, 2, and 3).

**Fig. 4:**
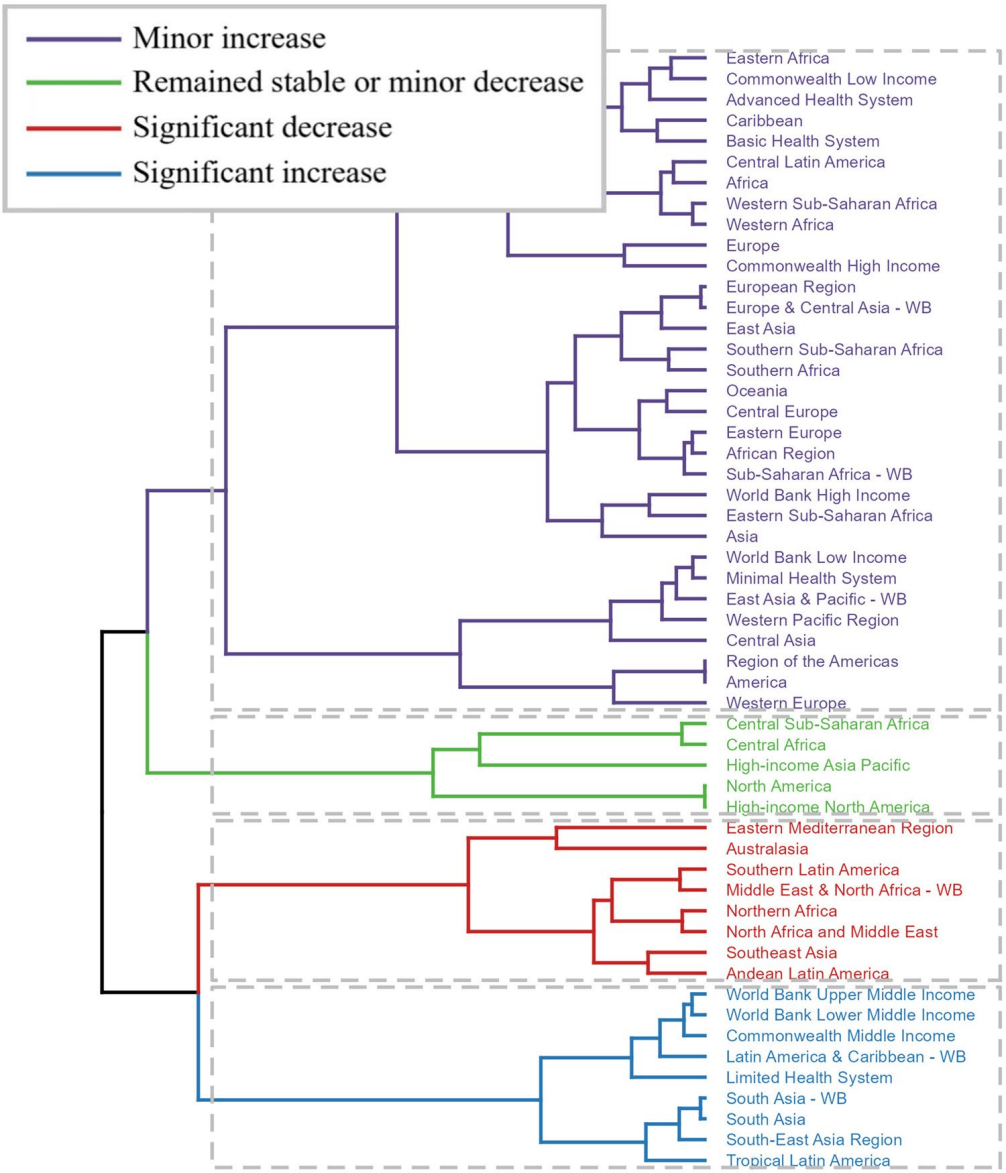
Results of cluster analysis based on the EAPC values of the osteoarthritis knee-related age-standardized rates for incidence, prevalence, and DALYs from 1990 to 2021. Abbreviations: EAPC, estimated annual percentage change; DALYs, disability-adjusted-life-years

To further elucidate the variability in osteoarthritis knee burden across GBD regions, we conducted a hierarchical clustering analysis. The results, presented in Fig. 4, reveal distinct clusters of regions sharing similar patterns of disease burden change. Notably, significant increases in ASRs were observed in regions spanning World Bank Upper Middle Income, World Bank Lower Middle Income, Commonwealth Middle Income, Latin America & Caribbean -WB, Limited Health System, South Asia -WB, South Asia, South-East Asia Region, and Tropical Latin America. In contrast, regions experiencing significant decreases in ASRs included Eastern Mediterranean Region, Australasia, Southern Latin America, Middle East & North Africa-WB, Northern Africa, North Africa and Middle East, Southeast Asia, and Andean Latin America (Fig. 4).

Across countries and and territories, the ASRs all showed a increasing trend except for United States of America. The most pronounced increase in ASIR and ASPR from 1990 to 2021 was observed in Republic of Equatorial Guinea [ASIR: EAPC = 0.6, 95% confidence interval (CI): 0.56–0.64; ASPR: EAPC = 0.68, 95% CI: 0.64–0.72] and Sultanate of Oman (ASIR: EAPC = 0.6, 95% CI: 0.58–0.62; ASPR: EAPC = 0.68, 95% CI: 0.66–0.70), while the most pronounced increase was only observed in Republic of Equatorial Guinea for ASDAR (EAPC = 0.71, 95% CI: 0.67–0.76) (Figure S3, Tables 1, 2, and 3).

### The influential factors for EAPC

The results demonstrate a discernible correlation between EAPCs and ASRs of osteoarthritis knee in 1990, as well as HDIs in 2021, respectively (Figure S4). Specifically, the ASRs recorded in 1990 for osteoarthritis knee reflect the baseline disease burden, whereas the HDIs in 2021 serve as indicators of healthcare accessibility and a surrogate measure for the health system's maturity within each country. The positive association was observed between EAPCs and ASRs across multiple health outcomes, including incidence (P = 0.56 ρ = 0.04), prevalence (P = 0.29 ρ = 0.07), and DALYs (P = 0.79 ρ = 0.02), though the association was not significant. Furthermore, the link between EAPCs for incidence, prevalence, and DALYs and HDIs was also reach statistical correlation. However, the correlation was negative for incidence (P = 0.68 ρ = −0.09), prevalence (P = 0.47 ρ = −0.15), and DALYs (P = 0.65 ρ = −0.10) of osteoarthritis knee with the respective HDIs (Figure S4).

### The predicted results from 2022 to 2046

The APC model showed that the number of incidence cases for males increased from 12.94 million to 18.45 million during the next 25 years, and it increased from 18.80 million to 25.60 million for females. The number of prevalence cases for males increased from 149.08 million in 2021 to 235.41 million in 2046, it increased from 236.39 million to 365.97 million for females. The number of DALYs cases increased from 4.82 million to 7.52 million for males, and it increased from 7.55 million to 11.55 million for females. The projected outcomes from the BAPC models also indicate an upward trend in the number of cases for both genders, spanning from 2022 to 2046. For males, the number of incidence cases increased from 16.45 million to 19.52 million, the number of prevalence cases increased from 200.10 million to 240.94 million, and the number of DALYs cases increased from 6.39 million to 7.56 million. For females, the number of incidence cases increased from 11.71 million to 13.85 million, the number of prevalence cases increased from 138.25 million to 166.31 million, and the number of DALYs cases increased from 4.47 million to 5.36 million.

**Fig. 5:**
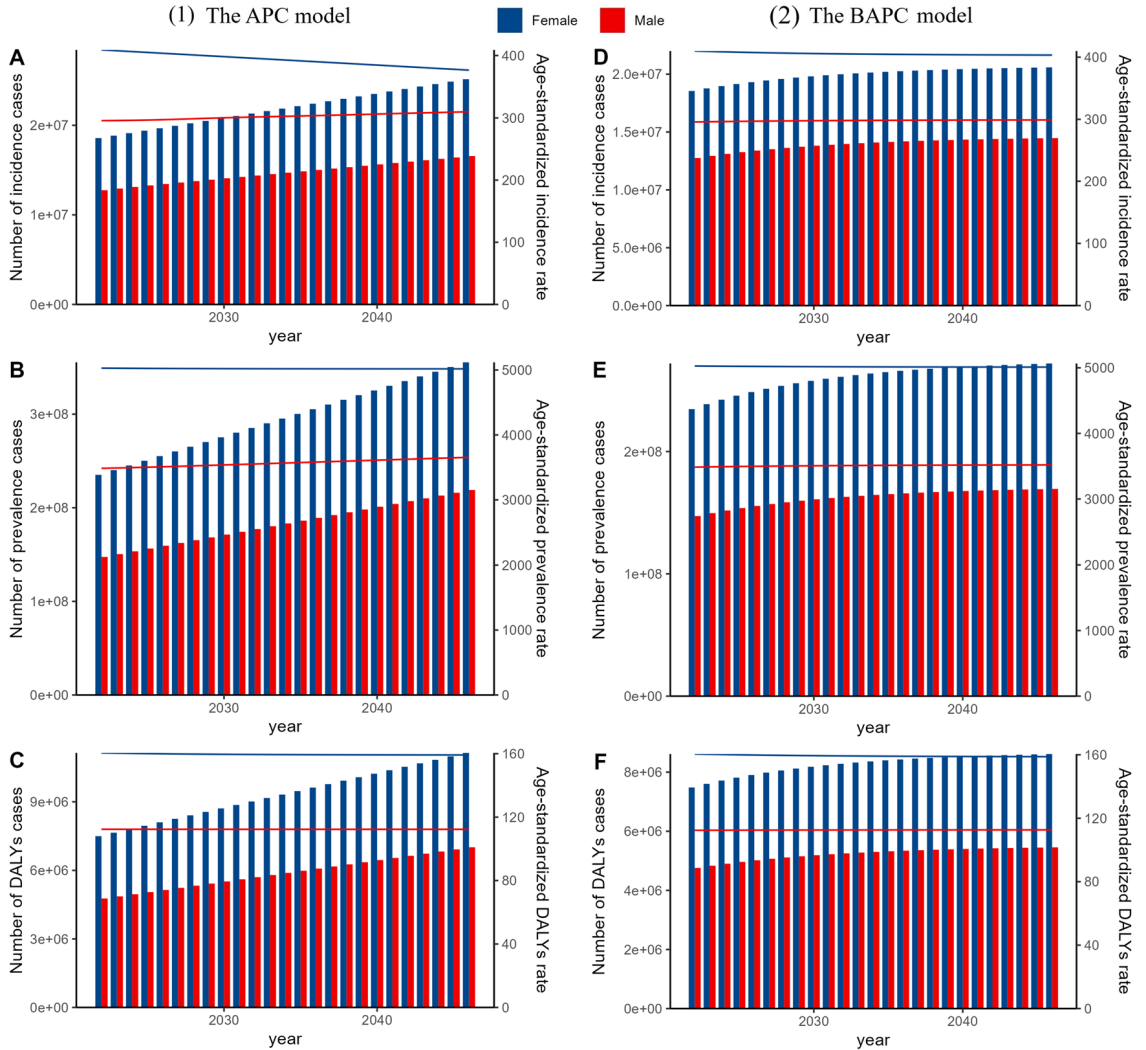
The predicted results in the osteoarthritis knee-related numbers and age-standardized rates of incidence, prevalence, and DALYs by sex globally from 2022 to 2046 of the APC model (1) and the BAPC model (2). Abbreviations: DALYs, disability-adjusted-life-year; APC, age-period-cohort; BAPC, Bayesian age-period-cohort

For ASRs, the prediction results showed a very slight change. The APC model showed that it showed a decreasing trend for females but exhibited a trend of first increase and then decrease for males. However, for the BAPC model, the ASRs for both genders exhibits a trend of first increase and then decrease (Figs. 5, Tables 4 and 5).

**Table 4:** The predicted results in the osteoarthritis knee-related numbers and age-standardized rates of incidence, prevalence, and DALYs by sex globally from 2022 to 2046 of the APC model

Year	Sex	Age-standardized incidence rate	Numer of incidence cases (million)	Age-standardized prevalence rate	Numer of prevalence cases (million)	Age-standardized DALYs rate	Numer of DALYs cases (million)
2022	Female	409.11	18.80	5013.61	236.39	160.17	7.55
2023	Female	408.54	19.14	5007.59	241.88	159.92	7.72
2024	Female	407.96	19.48	5001.57	247.50	159.68	7.89
2025	Female	407.35	19.82	4995.09	253.18	159.43	8.07
2026	Female	406.73	20.16	4988.60	258.89	159.18	8.25
2027	Female	406.12	20.50	4982.12	264.58	158.93	8.42
2028	Female	405.50	20.83	4975.64	270.31	158.68	8.60
2029	Female	404.88	21.15	4969.16	276.12	158.43	8.78
2030	Female	404.30	21.48	4961.59	281.90	158.16	8.96
2031	Female	403.71	21.80	4954.02	287.66	157.89	9.14
2032	Female	403.12	22.11	4946.45	293.35	157.62	9.31
2033	Female	402.53	22.41	4938.87	299.02	157.35	9.49
2034	Female	401.94	22.70	4931.30	304.72	157.08	9.66
2035	Female	401.43	22.99	4923.23	310.36	156.82	9.84
2036	Female	400.91	23.27	4915.16	315.93	156.55	10.01
2037	Female	400.40	23.55	4907.09	321.37	156.29	10.18
2038	Female	399.88	23.81	4899.02	326.74	156.02	10.34
2039	Female	399.37	24.06	4890.95	332.09	155.75	10.51
2040	Female	398.85	24.30	4881.94	337.26	155.46	10.67
2041	Female	398.34	24.54	4872.92	342.33	155.17	10.82
2042	Female	397.83	24.77	4863.90	347.24	154.88	10.97
2043	Female	397.31	24.99	4854.89	352.03	154.58	11.12
2044	Female	396.80	25.20	4845.87	356.78	154.29	11.27
2045	Female	396.29	25.40	4836.85	361.44	154.00	11.41
2046	Female	395.78	25.60	4827.83	365.97	153.70	11.55
2022	Male	295.72	12.94	3487.11	149.08	112.42	4.82
2023	Male	296.30	13.22	3491.24	152.81	112.53	4.94
2024	Male	296.87	13.49	3495.36	156.64	112.63	5.06
2025	Male	297.22	13.76	3498.24	160.45	112.70	5.18
2026	Male	297.57	14.02	3501.13	164.27	112.77	5.30
2027	Male	297.92	14.29	3504.01	168.08	112.84	5.42
2028	Male	298.27	14.55	3506.89	171.90	112.90	5.54
2029	Male	298.62	14.81	3509.78	175.79	112.97	5.66
2030	Male	298.73	15.06	3510.29	179.58	112.97	5.78
2031	Male	298.85	15.31	3510.80	183.36	112.97	5.90
2032	Male	298.96	15.55	3511.31	187.09	112.97	6.02
2033	Male	299.08	15.79	3511.82	190.82	112.96	6.13
2034	Male	299.19	16.03	3512.34	194.58	112.96	6.25
2035	Male	299.11	16.26	3510.66	198.22	112.90	6.37
2036	Male	299.02	16.48	3508.98	201.83	112.83	6.48
2037	Male	298.94	16.69	3507.30	205.36	112.77	6.59
2038	Male	298.85	16.90	3505.62	208.87	112.70	6.70
2039	Male	298.77	17.11	3503.94	212.39	112.64	6.81
2040	Male	298.64	17.31	3501.30	215.82	112.54	6.92
2041	Male	298.52	17.51	3498.65	219.21	112.45	7.02
2042	Male	298.39	17.70	3496.00	222.51	112.36	7.12
2043	Male	298.27	17.89	3493.35	225.76	112.26	7.23
2044	Male	298.14	18.08	3490.70	229.02	112.17	7.33
2045	Male	298.02	18.27	3488.05	232.24	112.07	7.43
2046	Male	297.89	18.45	3485.40	235.41	111.98	7.52

**Table 5:** The predicted results in the osteoarthritis knee-related numbers and age-standardized rates of incidence, prevalence, and DALYs by sex globally from 2022 to 2046 of the BAPC model

Year	Sex	Age-standardized incidence rate	Numer of incidence cases (million)	Age-standardized prevalence rate	Numer of prevalence cases (million)	Age-standardized DALYs rate	Numer of DALYs cases (million)
2022	Female	417.08	11.71	5073.79	138.25	162.04	4.47
2023	Female	418.51	11.85	5091.81	139.89	162.47	4.52
2024	Female	419.87	11.98	5109.41	141.52	162.89	4.57
2025	Female	421.14	12.11	5126.29	143.12	163.28	4.62
2026	Female	422.32	12.24	5142.57	144.67	163.65	4.67
2027	Female	423.46	12.36	5158.78	146.18	164.02	4.72
2028	Female	424.49	12.48	5174.41	147.66	164.36	4.77
2029	Female	425.43	12.60	5189.25	149.12	164.68	4.81
2030	Female	426.28	12.71	5203.21	150.53	164.97	4.86
2031	Female	427.02	12.82	5216.31	151.89	165.24	4.90
2032	Female	427.69	12.92	5228.99	153.20	165.48	4.94
2033	Female	428.24	13.02	5240.57	154.47	165.70	4.98
2034	Female	428.67	13.11	5251.09	155.69	165.88	5.02
2035	Female	429.02	13.20	5260.69	156.88	166.03	5.06
2036	Female	429.26	13.28	5269.27	158.00	166.15	5.09
2037	Female	429.43	13.36	5277.12	159.07	166.25	5.13
2038	Female	429.46	13.43	5283.44	160.09	166.30	5.16
2039	Female	429.38	13.50	5288.56	161.05	166.32	5.19
2040	Female	429.23	13.57	5292.82	161.98	166.31	5.22
2041	Female	429.00	13.63	5296.09	162.84	166.28	5.25
2042	Female	428.69	13.68	5298.54	163.65	166.22	5.27
2043	Female	428.26	13.73	5299.38	164.39	166.12	5.30
2044	Female	427.75	13.78	5299.11	165.08	165.98	5.32
2045	Female	427.18	13.82	5298.11	165.73	165.83	5.34
2046	Female	426.54	13.85	5296.20	166.31	165.65	5.36
2022	Male	295.19	16.45	3484.96	200.10	112.56	6.39
2023	Male	295.77	16.67	3492.33	202.81	112.79	6.47
2024	Male	296.33	16.89	3499.81	205.48	113.02	6.55
2025	Male	296.86	17.10	3507.09	208.10	113.24	6.63
2026	Male	297.34	17.30	3513.89	210.66	113.45	6.70
2027	Male	297.77	17.50	3520.41	213.20	113.64	6.78
2028	Male	298.17	17.69	3526.89	215.68	113.84	6.85
2029	Male	298.54	17.88	3533.31	218.09	114.03	6.92
2030	Male	298.87	18.06	3539.47	220.44	114.21	6.99
2031	Male	299.14	18.23	3545.02	222.71	114.37	7.05
2032	Male	299.36	18.40	3550.09	224.93	114.52	7.12
2033	Male	299.53	18.55	3554.81	227.06	114.65	7.18
2034	Male	299.66	18.70	3559.34	229.10	114.78	7.24
2035	Male	299.76	18.84	3563.57	231.07	114.90	7.29
2036	Male	299.80	18.98	3567.11	232.94	115.00	7.35
2037	Male	299.79	19.10	3570.02	234.75	115.08	7.40
2038	Male	299.72	19.22	3572.35	236.43	115.14	7.44
2039	Male	299.62	19.33	3574.35	238.03	115.19	7.49
2040	Male	299.49	19.43	3576.07	239.53	115.24	7.53
2041	Male	299.30	19.52	3577.12	240.94	115.26	7.56
2042	Male	299.07	19.60	3577.51	242.26	115.27	7.60
2043	Male	298.79	19.68	3577.22	243.46	115.26	7.63
2044	Male	298.48	19.74	3576.59	244.56	115.24	7.66
2045	Male	298.14	19.80	3575.72	245.57	115.21	7.69
2046	Male	297.76	19.85	3574.23	246.48	115.17	7.71

Collectively, these findings underscore the persistence of a relatively substantial future disease burden, which is further corroborated by the results obtained from the ARIMA model and the ES model (Figures S5, Tables S1-S2).

## Discussion

As far as we know, this was the first study to comprehensively assess and quantify osteoarthritis knee-related disease burden globally. Globally, it caused a great disease burden in 2021, and significant differences in the disease burden existed between sexes and across ages, SDI regions, GBD regions, and countries. From 1990 to 2021, there was an decreasing trend for the disease burden globally. Furthermore, our predicted results showed that the disease burden would increase in the next 25 years.

The current findings highlight a notable increase in the global burden of osteoarthritis knee from 1990 to 2021, with substantial rises in both incidence and prevalence cases. Our estimates of the age-standardized incidence and prevalence rates align with previous reports, underscoring the escalating trend in this condition worldwide [26–28]. Notably, the marked escalation in DALYs further emphasizes the substantial impact of osteoarthritis knee on population health and wellbeing.

Our gender-specific analysis underscores a higher burden of osteoarthritis knee among females, both in terms of incidence, prevalence, and DALYs, as compared to males. This disparity aligns with previous research reporting similar trends, reinforcing the notion that osteoarthritis knee disproportionately affects women [29–31]. The higher burden of osteoarthritis knee among females can be attributed to several social causes. Firstly, women are more likely to experience hormonal changes, particularly during menopause, which may contribute to the development and progression of osteoarthritis due to alterations in joint tissue metabolism and inflammation. Additionally, women are more prone to having specific anatomic features, such as wider hip bones and narrower femoral canals, which may increase the risk of knee osteoarthritis. Furthermore, societal norms often result in gender-specific roles and responsibilities, with women more frequently engaging in activities that place repetitive stress on their knees, such as childcare, household chores, and certain professions [32, 33]. These factors, combined with the global trend of increasing obesity among women [34], which is a significant risk factor for osteoarthritis, contribute to the higher incidence, prevalence, and DALYs associated with knee osteoarthritis among females compared to males. The congruency in trends observed between genders further highlights the uniformity in disease dynamics across these demographic subgroups, emphasizing the need for gender-sensitive strategies in managing and preventing osteoarthritis knee. As the disease burden in women continues to escalate, it underscores the urgency for tailored interventions that address the unique risk factors and healthcare needs of this population, aiming to mitigate the disproportionate impact of osteoarthritis knee on women's health and quality of life.

The age-stratified analysis of osteoarthritis knee incidence, prevalence, and DALYs in 2021 reveals a characteristic age-related pattern, with an initial rise followed by a peak in older age groups and subsequent decline. This trend mirrors previous findings, underscoring the disproportionate impact of osteoarthritis knee on the elderly population [35–37]. This trend can be attributed to several factors, including the increased utilization of prosthesis treatment among older individuals with severe osteoarthritis, which can effectively manage symptoms and reduce the disease burden in this age group. Additionally, changes in physical activity levels with age, such as a reduction in vigorous activities among the elderly, may also contribute to the observed decline in cases. However, it is important to note that the complex interplay between genetic, environmental, and behavioral factors likely plays a role in this age-related pattern. To support this explanation, we refer to the study by Cross, which similarly observed an age-related increase in osteoarthritis cases, peaking in older age groups [38]. Overall, these findings underscore the need for further research to fully understand the underlying causes of this age-related trend and to develop targeted interventions to mitigate the burden of osteoarthritis knee globally.

The observed disease burden across most age groups follows a similar trajectory, highlighting the importance of age-specific interventions and healthcare planning to address the unique needs of different age cohorts. As the global population ages, the need for targeted strategies to mitigate the burden of osteoarthritis knee, particularly among the elderly, becomes increasingly pressing. Our findings emphasize the significance of considering age as a critical factor in developing comprehensive prevention and management plans for this prevalent and disabling condition.

Our analysis of osteoarthritis knee burden across SDI regions reveals an interesting juxtaposition: while high SDI regions exhibited the highest age-standardized incidence, prevalence, and DALY rates, the middle SDI regions bore the greatest absolute number of cases. This paradoxical finding is consistent with prior studies highlighting the complexity of healthcare disparities across different socioeconomic strata [39–41]. The observed "J" relationship between SDI and ASRs underscores the dual challenge faced by low-SDI regions, grappling with limited healthcare resources, and high-SDI regions, grappling with aging populations and lifestyle-related factors. Importantly, the consistent trends across SDI regions for both ASRs and absolute case numbers, mirrored in the overall population, highlight the universality of osteoarthritis knee as a significant global health issue. This reinforces the need for globally coordinated efforts to address its rising burden, tailored to the unique socioeconomic contexts of different regions. As we strive to reduce the disease burden across all SDI levels, a comprehensive approach that encompasses preventive measures, access to timely and affordable treatment, and improvements in healthcare infrastructure, becomes paramount.

Our findings highlight substantial disparities in the burden of osteoarthritis knee across GBD regions and countries. High-income Asia Pacific emerges as a hotspot, particularly in terms of ASIR, ASPR, and ASDAR, underscoring the need for targeted interventions in this region. The dominance of Asia in overall case numbers, particularly China, India, and the USA, reflects the scale of the challenge globally. Conversely, Oceania and small island nations like Tokelau, Niue, and Nauru exhibit the lowest burdens, likely due to their small populations and differing healthcare systems. The hierarchical clustering analysis reveals distinct patterns of burden change, with notable increases in ASRs across several middle-income regions, suggesting a growing impact of osteoarthritis knee in these areas. Conversely, decreases in ASRs in some regions, such as Australasia and the Middle East, may reflect improvements in healthcare or lifestyle factors. However, the exception of the USA, where ASRs showed no overall increase, underscores the complexity of disease trends and the need for nuanced approaches to prevention and management. The pronounced increases in ASIR, ASPR, and ASDAR in Equatorial Guinea and Oman are particularly noteworthy, indicating rapid changes in disease burden that warrant urgent attention. These findings align with previous studies reporting increasing osteoarthritis prevalence in developing countries [42, 43], likely driven by factors such as aging populations, obesity, and changes in physical activity patterns.

The observed correlation between EAPCs and ASRs of osteoarthritis knee in 1990, while not statistically significant, suggests a potential historical impact on disease progression. In contrast, the negative correlation between EAPCs and HDIs in 2021 indicates that higher healthcare accessibility, as reflected by HDIs, does not necessarily lead to reduced osteoarthritis knee burden. This finding contrasts with previous studies reporting positive health outcomes associated with improved healthcare indices [44, 45]. The disparity may stem from osteoarthritis's multifactorial etiology, including non-modifiable risk factors, limiting the impact of healthcare accessibility alone. Additionally, the observed trends highlight the need for a nuanced approach to osteoarthritis management, incorporating both health system strengthening and targeted interventions addressing modifiable risk factors.

Our findings align with recent projections from various models, consistently indicating a looming increase in osteoarthritis incidence, prevalence, and DALYs across genders over the next two decades [46, 47]. While ASRs exhibit minimal variation under the APC model, the BAPC model reveals a nuanced trend of initial decline followed by an increase, highlighting the complexities of disease dynamics. This discrepancy underscores the importance of considering multiple modeling approaches to comprehensively assess future trends. Our results resonate with other studies utilizing ARIMA and ES models, reinforcing the robustness of the projected burden. Collectively, these findings emphasize the urgency for targeted interventions and strategies to mitigate the substantial future osteoarthritis burden.

The present study is constrained by its reliance on the GBD database, which presents several notable limitations. A primary limitation stems from the scarcity of detailed, subnational data at the county, province, and state levels, particularly in vast countries such as China [48]. This constraint necessitated a focus on broader, national-level indicators, thereby limiting the ability to provide more nuanced, region-specific insights into the burden of osteoarthritis knee. Moreover, the GBD database was an aggregated source that does not provide individual-level data, so detailed participant information such as demographics, lifestyles, or specific health metrics was not typically available at an individual level. This prevented us from conducting more in-depth studies, such as risk factor studies. Additionally, the accessibility of primary data within the GBD database poses a substantial challenge, as highlighted in previous studies [49, 50]. The reliance on estimated disease burden figures derived using the DisMod-MR, a standardized Bayesian regression framework, introduces another layer of complexity. While this approach facilitates the generation of estimates in data-sparse regions, it also necessitates the "borrowing" of information from comparable nations. This process, while valuable in filling data gaps, may introduce an element of uncertainty into the results, emphasizing the importance of cautious interpretation of the findings. Future research endeavors should strive to incorporate more comprehensive, subnational data and explore alternative methodologies to enhance the accuracy and precision of disease burden estimates, particularly in regions with limited data availability.

## Conclusion

In conclusion, osteoarthritis knee constitutes a substantial and growing global health burden. Our research underscores the vulnerability of females and older adults as distinct high-risk groups, emphasizing the need for targeted interventions. Furthermore, our analysis identifies regions with higher SDI as high-risk areas, highlighting disparities in disease burden across different populations. Notably, the predictive models indicate a projected increase in the number of cases for both genders from 2022 to 2046, underscoring the urgency for proactive measures. Consequently, the development and implementation of rigorous mitigation and adaptation strategies are imperative to protect individuals and effectively manage the burden of osteoarthritis knee, thereby reducing its impact on global health and wellbeing.

## Supplementary Information

Below is the link to the electronic supplementary material.

Supplementary file1 (DOCX 1579 KB)

## Data Availability

Please get in touch with the corresponding author for more information.
