# Supplementary material for: The global burden of osteoarthritis knee: a secondary data analysis of a population-based study
Source: Clin Rheumatol. 2025 Feb 12;44(4):1769–810. doi: 10.1007/s10067-025-07347-6 (PMC11993502; doi:10.1007/s10067-025-07347-6)

**The global burden of osteoarthritis knee: a secondary data analysis of a population-based study**

**Table legend**

**Table S1.** The predicted results in the osteoarthritis knee-related numbers and age-standardized rates of incidence, prevalence, and DALYs by sex globally from 2022 to 2046 of the ARIMA model.

**Table S2.** The predicted results in the osteoarthritis knee-related numbers and age-standardized rates of incidence, prevalence, and DALYs by sex globally from 2022 to 2046 of the ES model.

**Figure legend**

**Figure S1.** Numbers and age-standardized rates of osteoarthritis knee-related incidence, prevalence, and DALYs for both sexes (1), different age groups (2), different SDI regions (3), and different GBD regions (4) in 2021. Abbreviations: DALYs, disability-adjusted life years; SDI, socio-demographic index; GBD, Global Burden of Disease.

**Figure S2.** Trends in the numbers and age-standardized rates of osteoarthritis knee-related incidence, prevalence, and DALYs globally by both sexes (1), different age groups (2), and different SDI regions (3) from 1990 to 2021. Abbreviations: DALYs, disability-adjusted life years; SDI, socio-demographic index; GBD, Global Burden of Disease.

**Figure S3.** The EAPC of the osteoarthritis knee-related ASR of incidence, prevalence, and DALYs and the relative change in the numbers of incidence, prevalence, and DALYs between 1990 and 2021. Abbreviations: EAPC, estimated annual percentage change; ASR, age-standardized rate; DALYs, disability-adjusted-life-years.

**Figure S4.** The association between EAPCs and osteoarthritis knee-related ASRs in 1990 and HDIs in 2021. The circles represent countries that were available on HDI data. The size of the circle is increased with the cases of osteoarthritis knee. The ρ indices and p values presented were derived from Spearman correlation analysis. Abbreviations: EAPC, estimated annual percentage change; ASR, age-standardized rate; HDI, human development index.

**Figure S5.** The predicted results in the osteoarthritis knee-related numbers and age-standardized rates of incidence, prevalence, and DALYs by sex globally from 2022 to 2046 of the ARIMA model (1) and the ES model (2). Abbreviations: DALYs, disability-adjusted-life-year; ARIMA, Autoregressive Integrated Moving Average; ES, Exponential Smoothing.

**Table S1.** The predicted results in the osteoarthritis knee-related numbers and age-standardized rates of incidence, prevalence, and DALYs by sex globally from 2022 to 2046 of the ARIMA model.

| Year | Sex | Age-standardized incidence rate | Numer of incidence cases (million) | Age-standardized prevalence rate | Numer of prevalence cases (million) | Age-standardized DALYs rate | Numer of DALYs cases (million) |
| --- | --- | --- | --- | --- | --- | --- | --- |
| 2022 | Male | 295.40 | 12.75 | 3486.78 | 147.53 | 112.29 | 4.77 |
| 2023 | Male | 295.62 | 12.94 | 3491.58 | 150.51 | 112.28 | 4.86 |
| 2024 | Male | 295.91 | 13.12 | 3497.29 | 153.49 | 112.28 | 4.96 |
| 2025 | Male | 296.36 | 13.29 | 3503.58 | 156.47 | 112.27 | 5.05 |
| 2026 | Male | 296.96 | 13.45 | 3510.24 | 159.45 | 112.27 | 5.14 |
| 2027 | Male | 297.69 | 13.61 | 3517.15 | 162.43 | 112.26 | 5.24 |
| 2028 | Male | 298.48 | 13.77 | 3524.21 | 165.40 | 112.26 | 5.33 |
| 2029 | Male | 299.27 | 13.93 | 3531.38 | 168.38 | 112.26 | 5.42 |
| 2030 | Male | 300.01 | 14.09 | 3538.61 | 171.36 | 112.26 | 5.52 |
| 2031 | Male | 300.68 | 14.24 | 3545.88 | 174.34 | 112.25 | 5.61 |
| 2032 | Male | 301.29 | 14.40 | 3553.18 | 177.32 | 112.25 | 5.70 |
| 2033 | Male | 301.84 | 14.55 | 3560.49 | 180.30 | 112.25 | 5.80 |
| 2034 | Male | 302.38 | 14.71 | 3567.82 | 183.28 | 112.25 | 5.89 |
| 2035 | Male | 302.92 | 14.86 | 3575.15 | 186.26 | 112.25 | 5.98 |
| 2036 | Male | 303.49 | 15.02 | 3582.49 | 189.24 | 112.25 | 6.08 |
| 2037 | Male | 304.09 | 15.17 | 3589.83 | 192.22 | 112.25 | 6.17 |
| 2038 | Male | 304.72 | 15.33 | 3597.18 | 195.20 | 112.25 | 6.26 |
| 2039 | Male | 305.37 | 15.48 | 3604.52 | 198.18 | 112.25 | 6.36 |
| 2040 | Male | 306.02 | 15.64 | 3611.87 | 201.16 | 112.25 | 6.45 |
| 2041 | Male | 306.67 | 15.79 | 3619.21 | 204.13 | 112.25 | 6.54 |
| 2042 | Male | 307.30 | 15.95 | 3626.56 | 207.11 | 112.25 | 6.64 |
| 2043 | Male | 307.92 | 16.10 | 3633.91 | 210.09 | 112.25 | 6.73 |
| 2044 | Male | 308.53 | 16.26 | 3641.25 | 213.07 | 112.25 | 6.82 |
| 2045 | Male | 309.13 | 16.41 | 3648.60 | 216.05 | 112.25 | 6.92 |
| 2046 | Male | 309.73 | 16.57 | 3655.95 | 219.03 | 112.25 | 7.01 |
| 2022 | Female | 409.15 | 18.57 | 5027.56 | 235.20 | 160.41 | 7.50 |
| 2023 | Female | 407.79 | 18.85 | 5025.89 | 240.20 | 160.24 | 7.65 |
| 2024 | Female | 406.44 | 19.12 | 5024.46 | 245.21 | 160.09 | 7.80 |
| 2025 | Female | 405.08 | 19.40 | 5023.23 | 250.22 | 159.96 | 7.95 |
| 2026 | Female | 403.73 | 19.67 | 5022.17 | 255.23 | 159.85 | 8.10 |
| 2027 | Female | 402.37 | 19.94 | 5021.27 | 260.23 | 159.75 | 8.26 |
| 2028 | Female | 401.02 | 20.22 | 5020.49 | 265.24 | 159.66 | 8.41 |
| 2029 | Female | 399.66 | 20.49 | 5019.83 | 270.25 | 159.59 | 8.56 |
| 2030 | Female | 398.30 | 20.77 | 5019.26 | 275.25 | 159.52 | 8.71 |
| 2031 | Female | 396.95 | 21.04 | 5018.77 | 280.26 | 159.46 | 8.86 |
| 2032 | Female | 395.59 | 21.31 | 5018.35 | 285.27 | 159.41 | 9.02 |
| 2033 | Female | 394.24 | 21.59 | 5017.99 | 290.28 | 159.37 | 9.17 |
| 2034 | Female | 392.88 | 21.86 | 5017.68 | 295.28 | 159.33 | 9.32 |
| 2035 | Female | 391.53 | 22.14 | 5017.42 | 300.29 | 159.30 | 9.47 |
| 2036 | Female | 390.17 | 22.41 | 5017.19 | 305.30 | 159.27 | 9.62 |
| 2037 | Female | 388.81 | 22.68 | 5016.99 | 310.30 | 159.24 | 9.77 |
| 2038 | Female | 387.46 | 22.96 | 5016.83 | 315.31 | 159.22 | 9.93 |
| 2039 | Female | 386.10 | 23.23 | 5016.68 | 320.32 | 159.20 | 10.08 |
| 2040 | Female | 384.75 | 23.50 | 5016.56 | 325.33 | 159.18 | 10.23 |
| 2041 | Female | 383.39 | 23.78 | 5016.46 | 330.33 | 159.17 | 10.38 |
| 2042 | Female | 382.03 | 24.05 | 5016.37 | 335.34 | 159.16 | 10.53 |
| 2043 | Female | 380.68 | 24.33 | 5016.29 | 340.35 | 159.14 | 10.69 |
| 2044 | Female | 379.32 | 24.60 | 5016.22 | 345.36 | 159.13 | 10.84 |
| 2045 | Female | 377.97 | 24.87 | 5016.16 | 350.36 | 159.13 | 10.99 |
| 2046 | Female | 376.61 | 25.15 | 5016.12 | 355.37 | 159.12 | 11.14 |

**Table S2.** The predicted results in the osteoarthritis knee-related numbers and age-standardized rates of incidence, prevalence, and DALYs by sex globally from 2022 to 2046 of the ES model.

| Year | Sex | Age-standardized incidence rate | Numer of incidence cases (million) | Age-standardized prevalence rate | Numer of prevalence cases (million) | Age-standardized DALYs rate | Numer of DALYs cases (million) |
| --- | --- | --- | --- | --- | --- | --- | --- |
| 2022 | Male | 295.54 | 12.75 | 3487.42 | 147.23 | 112.36 | 4.76 |
| 2023 | Male | 295.90 | 12.94 | 3491.07 | 149.64 | 112.38 | 4.83 |
| 2024 | Male | 296.22 | 13.11 | 3494.36 | 151.82 | 112.41 | 4.90 |
| 2025 | Male | 296.51 | 13.26 | 3497.31 | 153.77 | 112.43 | 4.96 |
| 2026 | Male | 296.77 | 13.40 | 3499.97 | 155.53 | 112.45 | 5.02 |
| 2027 | Male | 297.00 | 13.52 | 3502.36 | 157.11 | 112.47 | 5.07 |
| 2028 | Male | 297.21 | 13.63 | 3504.52 | 158.54 | 112.49 | 5.11 |
| 2029 | Male | 297.40 | 13.73 | 3506.46 | 159.82 | 112.50 | 5.15 |
| 2030 | Male | 297.57 | 13.82 | 3508.20 | 160.98 | 112.51 | 5.19 |
| 2031 | Male | 297.72 | 13.90 | 3509.77 | 162.01 | 112.53 | 5.22 |
| 2032 | Male | 297.86 | 13.97 | 3511.18 | 162.95 | 112.54 | 5.25 |
| 2033 | Male | 297.99 | 14.04 | 3512.46 | 163.79 | 112.55 | 5.28 |
| 2034 | Male | 298.10 | 14.10 | 3513.60 | 164.55 | 112.56 | 5.30 |
| 2035 | Male | 298.20 | 14.15 | 3514.63 | 165.23 | 112.56 | 5.32 |
| 2036 | Male | 298.29 | 14.20 | 3515.56 | 165.84 | 112.57 | 5.34 |
| 2037 | Male | 298.37 | 14.24 | 3516.39 | 166.40 | 112.58 | 5.36 |
| 2038 | Male | 298.44 | 14.28 | 3517.14 | 166.89 | 112.58 | 5.38 |
| 2039 | Male | 298.51 | 14.31 | 3517.82 | 167.34 | 112.59 | 5.39 |
| 2040 | Male | 298.57 | 14.34 | 3518.43 | 167.74 | 112.59 | 5.40 |
| 2041 | Male | 298.62 | 14.37 | 3518.98 | 168.10 | 112.60 | 5.41 |
| 2042 | Male | 298.67 | 14.40 | 3519.47 | 168.43 | 112.60 | 5.42 |
| 2043 | Male | 298.71 | 14.42 | 3519.91 | 168.72 | 112.60 | 5.43 |
| 2044 | Male | 298.75 | 14.44 | 3520.31 | 168.99 | 112.61 | 5.44 |
| 2045 | Male | 298.79 | 14.46 | 3520.67 | 169.23 | 112.61 | 5.45 |
| 2046 | Male | 298.82 | 14.47 | 3520.99 | 169.44 | 112.61 | 5.46 |
| 2022 | Female | 409.72 | 18.55 | 5027.47 | 234.70 | 160.41 | 7.48 |
| 2023 | Female | 409.01 | 18.77 | 5025.64 | 238.75 | 160.23 | 7.60 |
| 2024 | Female | 408.38 | 18.97 | 5023.99 | 242.40 | 160.06 | 7.71 |
| 2025 | Female | 407.80 | 19.15 | 5022.50 | 245.69 | 159.92 | 7.81 |
| 2026 | Female | 407.29 | 19.31 | 5021.16 | 248.64 | 159.78 | 7.90 |
| 2027 | Female | 406.82 | 19.46 | 5019.96 | 251.31 | 159.66 | 7.98 |
| 2028 | Female | 406.40 | 19.59 | 5018.87 | 253.70 | 159.56 | 8.06 |
| 2029 | Female | 406.03 | 19.70 | 5017.90 | 255.86 | 159.46 | 8.12 |
| 2030 | Female | 405.69 | 19.81 | 5017.02 | 257.80 | 159.37 | 8.18 |
| 2031 | Female | 405.38 | 19.91 | 5016.23 | 259.54 | 159.29 | 8.23 |
| 2032 | Female | 405.11 | 19.99 | 5015.52 | 261.11 | 159.22 | 8.28 |
| 2033 | Female | 404.86 | 20.07 | 5014.88 | 262.53 | 159.16 | 8.33 |
| 2034 | Female | 404.64 | 20.14 | 5014.31 | 263.80 | 159.10 | 8.36 |
| 2035 | Female | 404.44 | 20.20 | 5013.79 | 264.95 | 159.05 | 8.40 |
| 2036 | Female | 404.26 | 20.26 | 5013.32 | 265.98 | 159.01 | 8.43 |
| 2037 | Female | 404.10 | 20.31 | 5012.90 | 266.91 | 158.96 | 8.46 |
| 2038 | Female | 403.95 | 20.35 | 5012.52 | 267.74 | 158.93 | 8.48 |
| 2039 | Female | 403.82 | 20.40 | 5012.18 | 268.49 | 158.89 | 8.51 |
| 2040 | Female | 403.70 | 20.43 | 5011.88 | 269.17 | 158.86 | 8.53 |
| 2041 | Female | 403.60 | 20.47 | 5011.60 | 269.78 | 158.84 | 8.55 |
| 2042 | Female | 403.50 | 20.50 | 5011.35 | 270.33 | 158.81 | 8.56 |
| 2043 | Female | 403.42 | 20.52 | 5011.13 | 270.82 | 158.79 | 8.58 |
| 2044 | Female | 403.34 | 20.55 | 5010.93 | 271.26 | 158.77 | 8.59 |
| 2045 | Female | 403.27 | 20.57 | 5010.75 | 271.66 | 158.75 | 8.60 |
| 2046 | Female | 403.21 | 20.59 | 5010.59 | 272.02 | 158.74 | 8.61 |

**Figure S1.** Numbers and age-standardized rates of osteoarthritis knee-related incidence, prevalence, and DALYs for both sexes (1), different age groups (2), different SDI regions (3), and different GBD regions (4) in 2021. Abbreviations: DALYs, disability-adjusted life years; SDI, socio-demographic index; GBD, Global Burden of Disease.

**
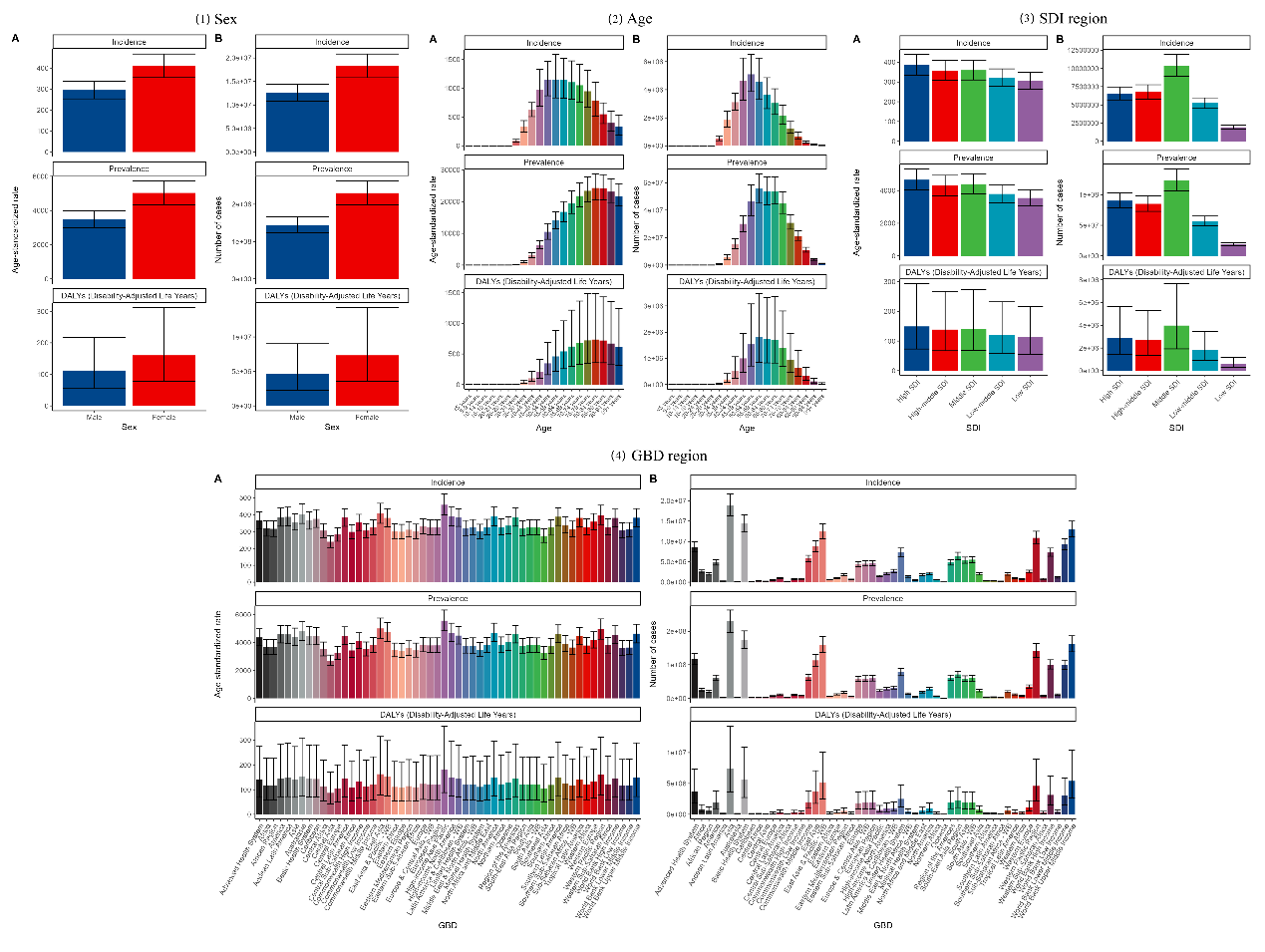
**

**Figure S2.** Trends in the numbers and age-standardized rates of osteoarthritis knee-related incidence, prevalence, and DALYs globally by both sexes (1), different age groups (2), and different SDI regions (3) from 1990 to 2021. Abbreviations: DALYs, disability-adjusted life years; SDI, socio-demographic index; GBD, Global Burden of Disease.

**
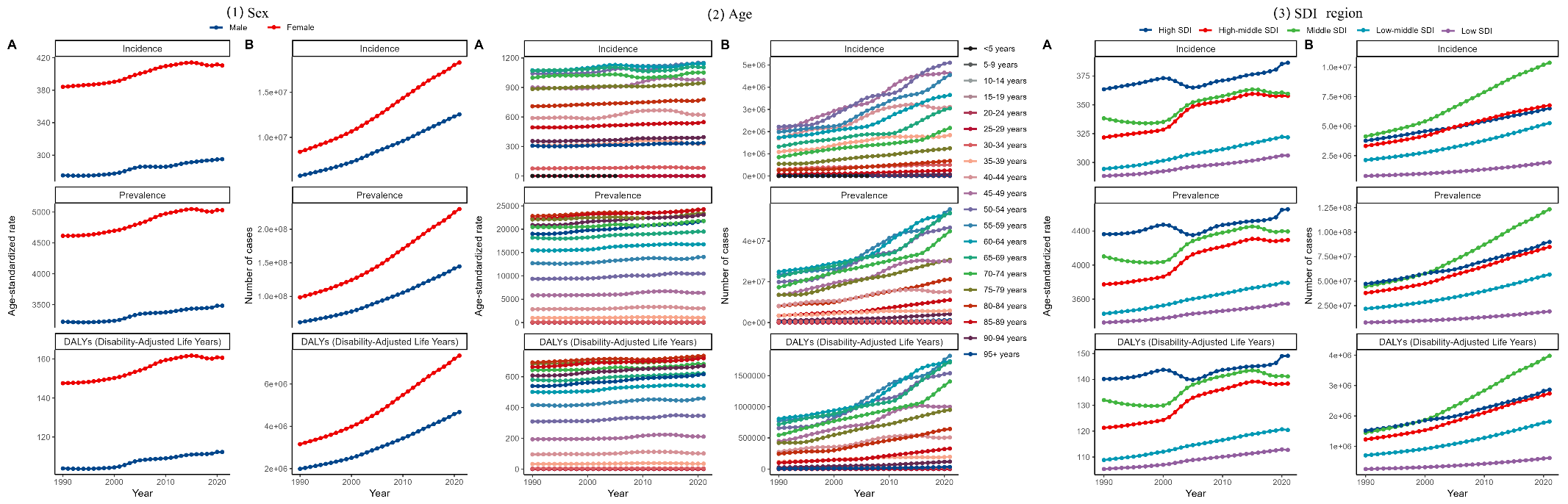
**

**Figure S3.** The EAPC of the osteoarthritis knee-related ASR of incidence, prevalence, and DALYs and the relative change in the numbers of incidence, prevalence, and DALYs between 1990 and 2021. Abbreviations: EAPC, estimated annual percentage change; ASR, age-standardized rate; DALYs, disability-adjusted-life-years.


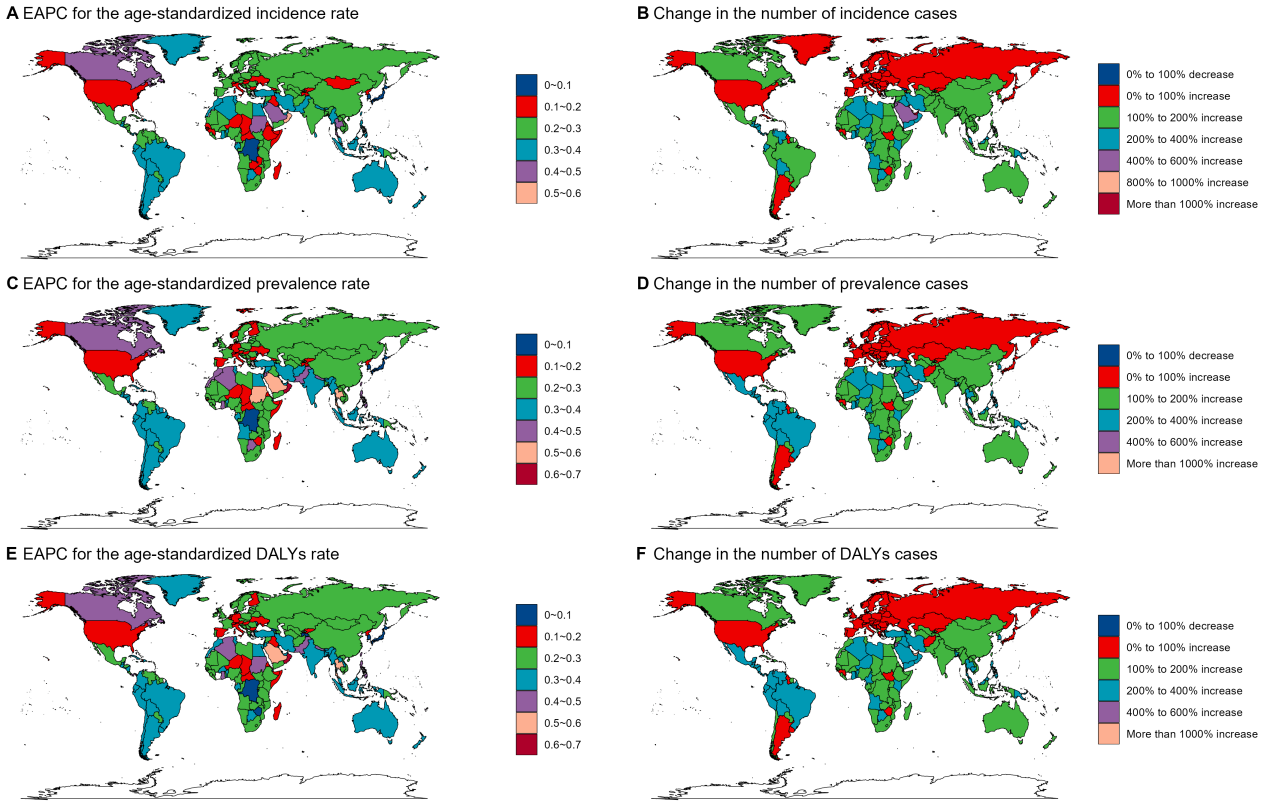


**Figure S4.** The association between EAPCs and osteoarthritis knee-related ASRs in 1990 and HDIs in 2021. The circles represent countries that were available on HDI data. The size of the circle is increased with the cases of osteoarthritis knee. The ρ indices and p values presented were derived from Spearman correlation analysis. Abbreviations: EAPC, estimated annual percentage change; ASR, age-standardized rate; HDI, human development index.


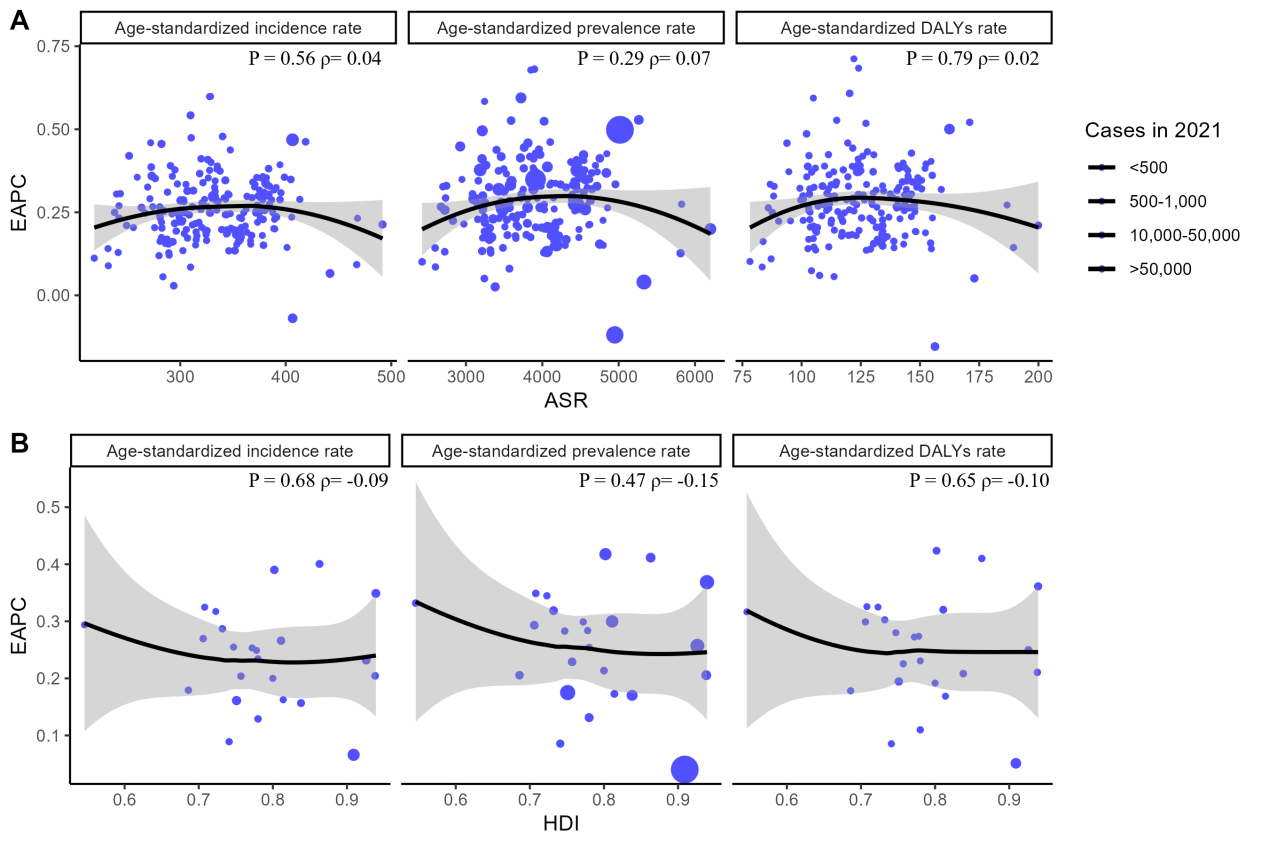


**Figure S5.** The predicted results in the osteoarthritis knee-related numbers and age-standardized rates of incidence, prevalence, and DALYs by sex globally from 2022 to 2046 of the ARIMA model (1) and the ES model (2). Abbreviations: DALYs, disability-adjusted-life-year; ARIMA, Autoregressive Integrated Moving Average; ES, Exponential Smoothing.


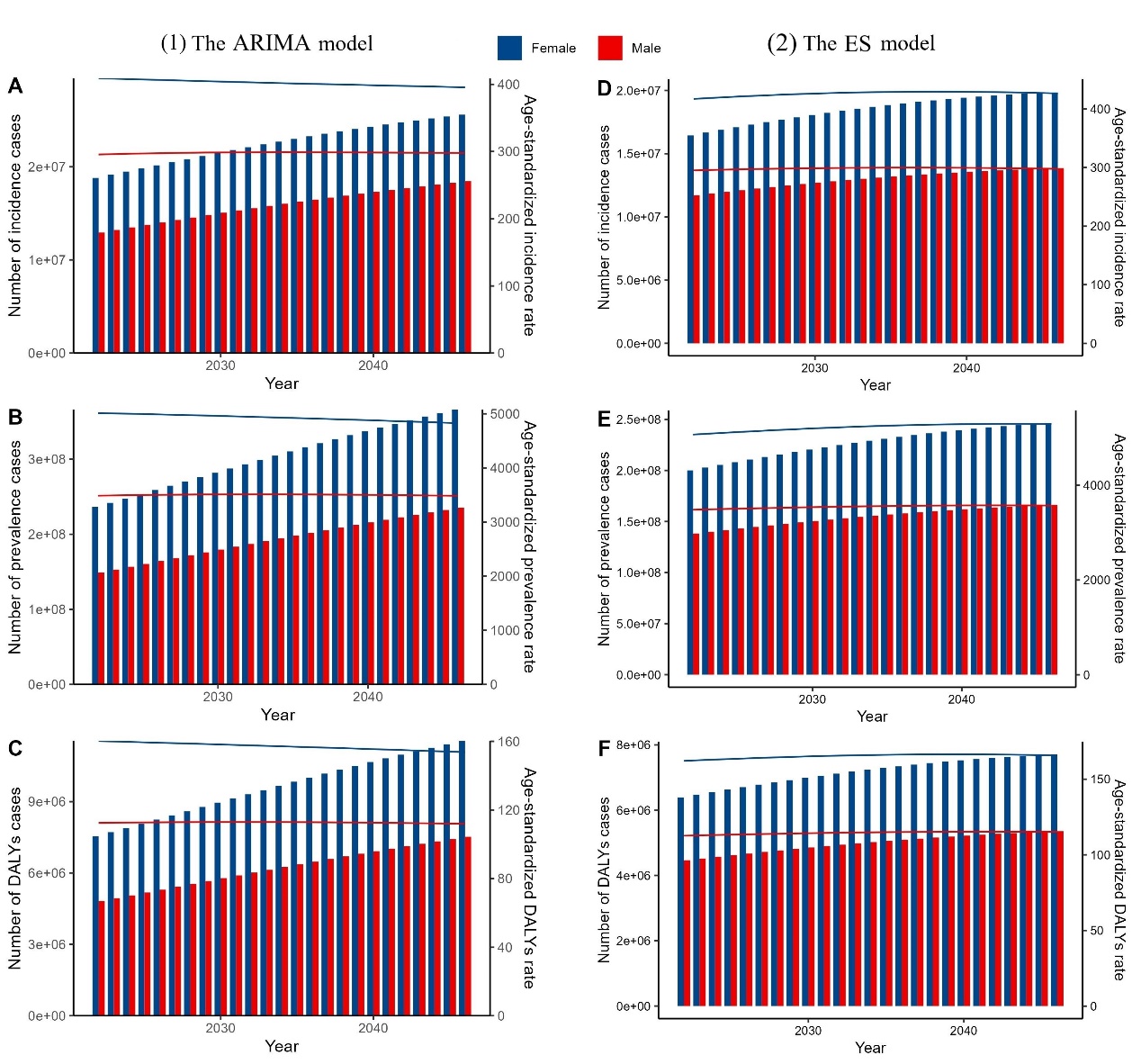

Supplement: Supplementary file 1 — Supplementary file1 (DOCX 1579 KB) [file 10067_2025_7347_MOESM1_ESM.docx]
